# Loop extrusion by cohesin plays a role in enhancer-activated gene expression early in differentiation

**DOI:** 10.1038/s41467-026-73049-5

**Published:** 2026-05-26

**Authors:** Rosa J. Stolper, Felice H. Tsang, Emily Georgiades, Lars L. P. Hanssen, Edward A. J. Tunnacliffe, Damien J. Downes, Caroline L. Harrold, Jim R. Hughes, Robert A. Beagrie, Benjamin Davies, Mira T. Kassouf, Douglas R. Higgs

**Affiliations:** 1 MRC Weatherall Institute of Molecular Medicine, Radcliffe Department of Medicine, University of Oxford, John Radcliffe Hospital, Oxford, UK; 2 Chinese Academy of Medical Sciences Oxford Institute, Nuffield Department of Medicine, University of Oxford, Oxford, UK; 3 Present Address: Wellcome Centre for Human Genetics, Nuffield Department of Medicine, University of Oxford, Oxford, UK; 4 Present Address: Francis Crick Institute, London, UK

**Keywords:** Transcriptional regulatory elements, Chromatin structure

## Abstract

Enhancers and their target promoters often come into close physical proximity when activated. This may be explained by a variety of mechanisms, including cohesin-mediated chromatin loop extrusion. However, acute depletion of cohesin does not cause widespread changes in gene expression. We have tested the role of cohesin-mediated loop extrusion in gene expression at the mouse alpha-globin locus during erythropoiesis. Acute depletion of cohesin disrupts alpha-globin expression at early but not late stages of differentiation. Furthermore, when single or multiple CTCF sites, known to block cohesin, are placed between the alpha-globin enhancers and promoters, alpha-gene expression is disrupted. Importantly, the CTCF site’s orientation is critical, suggesting that within this activated domain, in definitive erythroid cells, cohesin predominantly but not exclusively, translocates from the enhancers to the promoters. We find that loop extrusion does play an important role in establishing enhancer-promoter proximity and consequent expression of inducible genes during differentiation.

## Abstract (web summary)

Cohesin depletion shows that loop extrusion drives enhancer-promoter contact and expression during early differentiation at the alpha-globin locus. CTCF barriers reveal predominantly unidirectional tracking of cohesin from enhancers toward promoters.

## Introduction

It is now widely accepted that the process of loop extrusion, mediated by the cohesin complex and delimited by convergently-orientated CTCF binding sites, plays a key role in packaging chromatin into large (100s-1000s kb) topologically associating domains (TADs)^1,2^. It has been noted that TADs often include both the enhancers and promoters of specific, independent regulatory domains and are separate from other similarly organised regulatory units^3^. Structural variants disrupting TADs often lead to abnormal patterns of gene expression^4–7^. Throughout lineage-specification and differentiation, sub-TADs, containing enhancer-promoter interactions, appear, and it has been proposed that translocation of cohesin may juxtapose these linearly separated regulatory elements thus playing a role in initiating transcriptional activation. Contrary to what would be expected if this was the case, in most current reports, although TADs largely disappear when cohesin is downregulated, gene expression is not affected to the extent predicted^8–12^.

Here, we have set out to further explore the role of cohesin and loop extrusion in the regulation of mouse alpha-globin gene expression. The alpha-globin genes lie in a 165 kb TAD that is present in several cell types^13^, but during differentiation of erythroid cells a 65 kb sub-TAD appears, containing the five alpha-globin enhancers and their cognate promoters lying 30-50 kb downstream. During differentiation, largely convergently orientated CTCF sites flanking the sub-TAD come into close proximity based on chromosome conformation capture and super-resolution microscopy^14–16^. The onset and continuation of gene expression and transcriptional bursting correlates with the increased physical proximity of the enhancers and promoters. These findings are consistent with the loop extrusion hypothesis and the alpha-globin model thus provides an opportunity to test this experimentally.

We have tested the role of loop extrusion in enhancer-activated alpha-globin expression in two ways. First, we depleted cohesin during erythroid differentiation and assessed its effect on alpha-globin expression. Then, we took advantage of an alternative way of compromising loop extrusion, by placing an ectopic CTCF site at the alpha-globin locus. Once cohesin is loaded, it continues to translocate until it reaches a boundary element that blocks further translocation, or the loop extrusion complex dissociates from the chromatin fibre. Although this process may be stalled by a variety of protein complexes and nuclear processes such as transcription^17–21^, the 11-zinc finger protein CTCF is thought to be the most important block to loop extrusion. Many CTCF binding sites and transcriptionally active genes are found at TAD boundaries^22^. Interestingly, the CTCF binding sites flanking TADs and sub-TADs are usually convergently-oriented^23^. This can be explained by a specific interaction between the N-terminus of CTCF and the RAD21-STAG subunits of cohesin which is thought to occur when translocation is stalled^24,25^. Using the alpha-globin model, we have investigated the effect of inserting one or multiple well characterised CTCF binding sites between the alpha-globin enhancers and promoters in either orientation. This has allowed us to test the influence of CTCF sites, in either orientation, in blocking translocation and the impact of this on gene expression.

Although cohesin-mediated loop extrusion is a popular model to explain enhancer-promoter interactions, experimental evidence addressing this is not yet conclusive, particularly for interactions of less than 100kb^26^. In addition, the relatively small effects of removing cohesin on gene expression is unexpected^12^. Although loop extrusion may explain how enhancers and promoters come into close proximity, other mechanisms have also been proposed^27–29^. Indeed, in certain instances cohesin seems to interrupt cis-interactions, thereby disrupting transcription and gene expression^26^. Here, by analysing a single locus that is progressively induced during differentiation, we show that cohesin-mediated loop extrusion provides a plausible mechanism by which enhancers and promoters come into proximity early in differentiation and that interrupting this process leads to significant changes in gene expression.

## Results

### Alpha-globin expression is sensitive to cohesin depletion at the earlier stages of erythroid differentiation

**Fig. 1:**
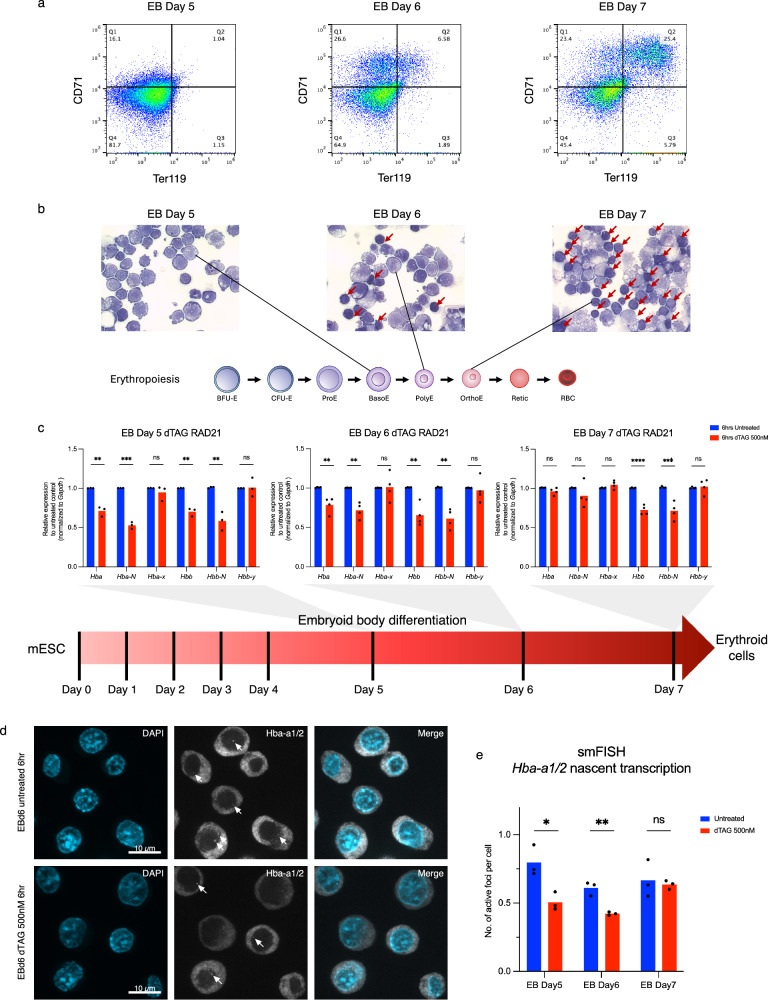
Alpha-globin expression is sensitive to cohesin depletion at the earlier stages of erythroid differentiation. **a** FACS profiles showing differentiation of mESCs along the erythroid lineage in disaggregated embryoid bodies (EBs) at days 5, 6 and 7 of culture as marked by the increased enrichment in erythroid-specific cell surface markers (CD71 and Ter119). **b** Stained (Wright-Giemsa) cells sampled from the populations above EBs at days 5, 6 and 7 reveal cells at varying stages of erythroid differentiation. Maroon arrows indicate the orthochromatic erythroblasts**. c** Gene expression by qPCR in EB-derived CD71+ erythroid cells with or without dTAG treatment at day 5 (*n* = 3), day 6 (*n* = 4) and day 7 (*n* = 4) of differentiation. Below, a schematic representing the days of EB differentiation. **d** Representative images of EB-derived CD71+ erythroid cells stained for smFISH at day 6 of differentiation, with or without dTAG treatment. Foci are visible inside the nuclei of actively transcribing cells (white arrows). **e** Number of active foci of alpha-globin expression per cell at day 5, 6 and 7 EB-derived CD71+ erythroid cells, with or without dTAG treatment (*n* = 3, 200–300 cells analysed for each). *P*-values were obtained using an unpaired two-tailed Student’s *t*-test on log-transformed fold change: Non-significant (ns), *P* > 0.05, *P ≤ 0.05, **P < 0.01, ***P < 0.001, ****P < 0.0001. Exact *P*-values are provided in the Source Data file.

To test the importance of cohesin in the regulation of alpha-globin expression, we created a mouse embryonic stem cell (mESC) line in which both copies of the cohesin subunit *Rad21* gene are tagged with the FKBP protein. This allows for rapid degradation of RAD21 using the dTAG system^30^ and thereby depletion of the cohesin complex. We differentiated these cells along the erythroid lineage, using a well-established in vitro embryoid body (EB) differentiation method^31^. During the EB differentiation, cells start to express the globin genes at around day 5 and their expression increases on subsequent days; previously, erythroid cells isolated from day 7 EBs were established as an optimal population to study alpha-globin expression^31^ (Fig. 1a and b).

Initially, we purified erythroid Rad21-FKBP cells from day 7 EBs and treated this population with 500 nM dTAG for 6 hours, which led to rapid and complete degradation of RAD21 as judged by western blot and ChIP-seq analysis (Supplementary Figs. 1 and 2). We determined expression levels of alpha- and beta-globin (*Hba-a* and *Hbb-b*) using RT-qPCR and observed no change in alpha-globin expression. However, we noted a 28% reduction of beta-globin expression (Fig. 1c). We also used primers that amplify the intron-exon junctions of the alpha- and beta-globin genes to evaluate nascent transcription (*Hba-N, Hbb-N*), which showed similar patterns to those of mature transcripts (Fig. 1c and Supplementary Fig. 3c). The changes in expression were recapitulated using digital droplet PCR (ddPCR) (Supplementary Fig. 4c). Finally, we looked at the embryonic globin genes (*Hba-x* and *Hbb-y*) and observed no change in their levels of expression following depletion of cohesin (Fig. 1c and Supplementary Fig. 3c).

Expression of beta-globin is normally delayed relative to alpha-globin in the EB differentiation system^31^. We wondered if this played a role in the sensitivity of beta- but not alpha-globin expression to cohesin degradation at day 7. Furthermore, of interest, it has previously been shown that cohesin may be especially important during changes in gene expression rather than in steady-state expression^32^. We therefore targeted earlier populations of erythroid cells, isolated from day 5 and 6 EBs, when alpha-globin expression is changing from very low to very high levels. Depleting RAD21 for 6 hours in these earlier populations had a clear and significant effect on both alpha- and beta-globin RNA expression (Fig. 1c, Supplementary Figs. 3a, 3b, 4a, and 4b), with the less mature erythroid cells derived from day 5 EBs showing the most severe effect on alpha-globin expression upon cohesin depletion (29%). However, the expression of other non-erythroid genes around the alpha-globin locus did not change throughout the EB differentiation (Supplementary Fig. 5).

To further investigate the effect of cohesin depletion on alpha-globin expression, we employed single-molecule RNA fluorescent in-situ hybridisation (smFISH). smFISH reveals sites of active transcription inside nuclei (Fig. 1d) and can thus be used to measure nascent transcription. We quantified nascent transcription by counting the number of foci present in nuclei at different stages during EB differentiation. In general, a slight decrease of nascent transcription was observed at the later stages of differentiation (Fig. 1e), which is consistent with our previous study of nascent transcription at the alpha-globin locus^33^. Upon RAD21 degradation, the number of actively transcribing cells were substantially lower in the early populations of EBs (Fig. 1e, Supplementary Fig. 6), and the number of active transcription foci per cell was also significantly reduced in the day 5 EBs (36%, *p* = *0.019*) and day 6 EBs (30%, *p* = *0.003*) (Fig. 1e). However, consistent with the RT-qPCR and ddPCR data, nascent transcription of the alpha-globin gene was not affected by RAD21 degradation in day 7 EBs (Fig. 1e).

Next, we studied the 3D chromatin organisation of the alpha-globin locus by Capture-C^34^ to interrogate the effect of cohesin depletion on the enhancer-promoter interaction. Capture-C from the *Hba-a* promoters and the R2 alpha-globin enhancer have revealed a consistent trend of reduced enhancer-promoter interaction upon cohesin depletion in day 5 EBs (Supplementary Fig. 7). However, the differences are not statistically significant. We also observed that the effect of cohesin depletion on enhancer-promoter interaction in day 7 EBs is less profound, which is consistent with the gene expression data.

In summary, expression of alpha-globin is sensitive to acute depletion of cohesin while it is being highly induced during the early stages of erythroid differentiation.

### Insertion of a CTCF insulator element in either orientation between the alpha-globin enhancers and the alpha-genes

Translocation of cohesin can be blocked by CTCF, and we therefore reasoned that placing a CTCF binding site ectopically between the alpha-globin enhancers and the alpha-globin genes would be an orthogonal way of testing the importance of loop extrusion for alpha-globin gene activation. The insertion of an ectopic binding site would offer the benefit of blocking loop extrusion throughout differentiation in a locus-specific manner. Additionally, since CTCF predominantly but not exclusively blocks loop extrusion from a specific direction in relation to its non-palindromic binding site, inserting it in either orientation could offer insight into the direction of loop extrusion. We therefore aimed to create two models: one with an ectopic CTCF binding site oriented towards the alpha-globin enhancers, blocking loop extrusion from that direction, and one in which the binding site is oriented to block loop extrusion from the direction of the promoters.

**Fig. 2:**
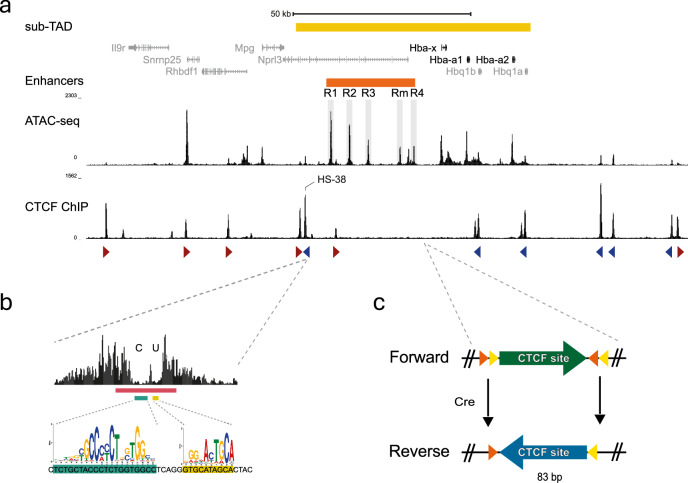
Insertion of a CTCF insulator element in between the alpha-globin enhancer cluster and the alpha-globin genes. **a** The mouse alpha-globin locus lies in a 65 kb sub-TAD (yellow bar) and contains two adult alpha-globin genes (*Hba-a1 and Hba-a2*) and an embryonic gene, zeta-globin (*Hba-x*). Open chromatin peaks (ATAC-seq track^31^) mark five upstream enhancer-like elements, four of which are located in the introns of the *Nprl3* gene. CTCF binding sites (CTCF-seq track^31^) are marked by red (forward-orientated) and blue (reverse-oriented) arrows. **b** The 5’ boundary element^36^ CTCF binding site (HS-38) is depicted with its 50 bp DNaseI footprint showing a clear orientation with a core (marked C, green bar) and upstream (marked U, yellow bar) motif. The red bar indicates the 83 bp sequence used for CTCF sequence insertions in this study. **c** The 83 bp sequence indicated in b is inserted between the alpha-globin genes and their enhancer-like elements, 26 Kb and 2.5 Kb downstream from R1 and R4, respectively, 5 kb and 11.7 Kb upstream from of *Hba-x* and *Hba-a1,* respectively. Heterospecific lox sites (orange triangles: lox2272, yellow triangles: loxP) flank the new inserted CTCF site and enable the inversion of the site orientation (from Forward to Reverse) by Cre recombinase.

To select a CTCF binding site sequence for ectopical insertion, we considered the CTCF binding sites within the alpha-globin locus that have been mutated and characterised in detail in previous studies^35,21^. Although deletion of these CTCF sites led to changes in chromatin interactions, deletion of nearly all single CTCF sites had little or no effect on gene expression. The one exception was the deletion of a CTCF site (HS-38) located at the 5’ boundary of the alpha-globin sub-TAD^13^ (Fig. 2a–b). This CTCF binding site normally restricts the influence of the alpha-globin enhancers on genes lying 5’ (upstream) of the globin cluster. Deletion of HS-38 leads to upregulation of these genes^35^. DNaseI hypersensitivity data reveals two footprints for this binding site: one overlapping with the core motif for CTCF and one corresponding to a previously described upstream motif (Fig. 2b)^36^. This means it is possible to confidently determine the orientation of this binding site. Therefore, this binding site appeared a good candidate to block loop extrusion between the alpha-globin enhancers and the alpha-genes. We selected an 83-bp sequence, encompassing the entire HS-38 DNaseI footprint, and placed this sequence between the most 3’ enhancer (R4) and the most 5’ globin gene, *Hba-x* (Fig. 2c).

The CTCF site was initially oriented convergent with downstream CTCF binding sites and towards the alpha-globin genes, and we termed this the “Forward” model. The ectopic CTCF binding site was flanked by heterospecific lox sites, to enable its inversion to create the “Reverse” model, in which the CTCF site is convergent with upstream CTCF binding sites and oriented towards the enhancer cluster (Fig. 2a, c). Initially, we used CRISPR/Cas9-mediated homology-directed repair to introduce this sequence into mESC, and followed by blastocyst injection to obtain the Forward and Reverse mouse models. Again, we also used in vitro EB differentiation system to produce erythroid cells from the mESC models, harvesting at the day 7 timepoint. From the engineered mouse models, we analysed spleen and fetal liver-derived erythroid cells as well as E10.5 embryonic blood.

**Fig. 3:**
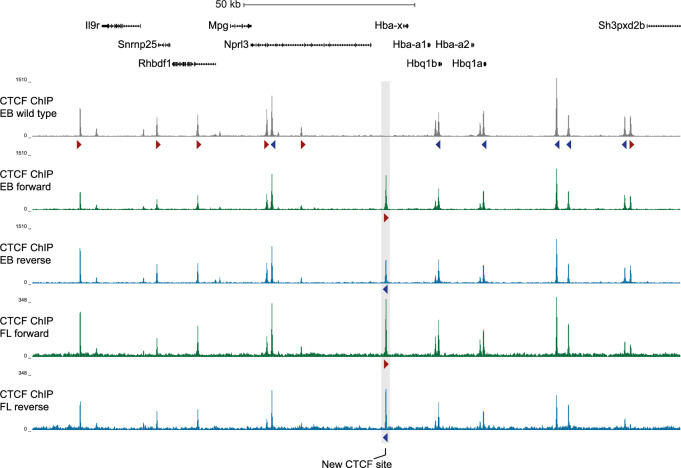
CTCF ChIP-seq confirms recruitment of CTCF to the newly introduced CTCF binding site. RPKM-normalised CTCF ChIP-seq tracks in wild-type^31^ (grey), Forward (green) and Reverse (blue) from day 7 EB-derived erythroid cells (EB, *n* = 2 for each) and fetal liver-derived erythroid cells (FL, *n* = 3 for each). CTCF binding site orientation is indicated by forward arrows (red) and reverse arrows (blue). The insertion site is highlighted in grey.

To confirm that CTCF binds to the newly introduced ectopic site, we generated CTCF ChIP-seq in both the Forward and Reverse models in EB- and fetal liver-derived erythroid cells (Fig. 3). This verified CTCF binding to the newly introduced ectopic site; there were little, if any, changes at existing CTCF binding sites. Importantly, the ectopic ChIP-seq peak had a similar level of binding regardless of its orientation, showing efficient CTCF recruitment to this site in both models. We also assessed the open chromatin landscape by generating ATAC-seq datasets in both wild-type cells and those with the ectopic insertion. Again, insertion of the ectopic CTCF binding site did not result in significant changes to surrounding hypersensitive sites compared to wild-type cells, establishing that the insertion does not disturb the overall integrity of the locus and the enhancers are still accessible (Supplementary Fig. 8). The new CTCF site is marked by a small accessible chromatin peak, similar to other CTCF binding sites at the locus.

### Cohesin is present primarily at the reverse oriented CTCF site in definitive erythroid cells

Cohesin is usually present at most of the CTCF sites at the alpha-globin locus, and at a lower level at some other sites, such as the enhancers^35^. To probe any changes in cohesin binding upon the introduction of the ectopic binding site, we performed ChIP-seq for the cohesin subunit RAD21 and STAG2 in the Forward and Reverse models.

**Fig. 4:**
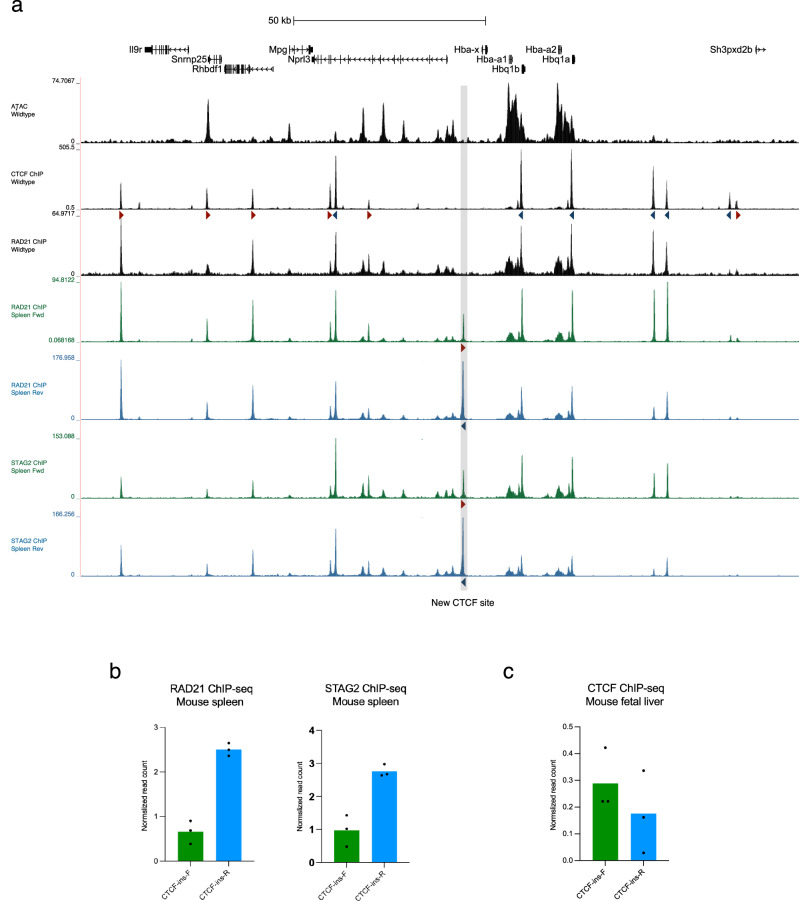
RAD21 ChIP-seq at the CTCF insertion site. **a** Top three tracks: ATAC-seq, CTCF ChIP-seq, and RAD21 ChIP-seq tracks in wild-type erythroid cells. Fourth and fifth tracks: RPKM-normalised RAD21 ChIP-seq tracks in Forward (Fwd, green, *n* = 3) and Reverse (Rev, blue, *n* = 3) CTCF insertion models in each of the mouse spleen-derived erythroid cells. Sixth and seventh tracks: RPKM-normalised STAG2 ChIP-seq tracks in Forward (Fwd, green, *n* = 3) and Reverse (Rev, blue, *n* = 3) CTCF insertion models in each of the mouse spleen-derived erythroid cells. CTCF binding site orientation is indicated by forward arrows (red) and reverse arrows (blue). The insertion site is highlighted in grey. **b** RAD21 and STAG2 ChIP read counts at the CTCF insertion site normalised to the average number of reads at all the other peaks in the genome from ChIP-seq data, in both Forward (CTCF-ins-F) and Reverse (CTCF-ins-R) CTCF insertion models. **c** Same as in b for CTCF ChIP read counts. Black dots represent single datapoints. Due to the small number of biological replicates (*n* = 3), statistical power was limited (Mann–Whitney *U*-test, two-side *p* = 0.1).

We and others have shown that the recruitment, translocation and stalling of cohesin may be affected by transcriptionally active genes^20,21,37^. In EB-derived erythroid cells the embryonic *Hba-x* gene, which lies between the enhancers and the adult alpha-globin genes, is active and may play a role in both recruiting and/or stalling translocation of cohesin^31^ (discussed in Supplementary Fig. 9). Therefore, we chose to examine the distribution of cohesin in primary definitive erythroid cells from the spleens of the engineered Forward and Reverse mouse models to examine the distribution of cohesin at the alpha-globin cluster in which only the adult alpha-globin genes are transcribed. This showed prominent peaks of RAD21 and STAG2 in the Reverse model and a smaller peak in the Forward model (Fig. 4a) despite very little difference in CTCF binding between the Forward and Reverse models (Fig. 3).

We quantified the number of reads in a 1.5 kb window surrounding the ectopic CTCF binding site and normalised this to the average number of reads over genome-wide CTCF peaks (Fig. 4b). This revealed that there is enrichment of RAD21 and STAG2 at the ectopic site in the Forward model, but there are more reads in the Reverse model. In contrast, we observed that although the CTCF levels are variable, it is consistently present in both the Forward and Reverse models (Fig. 4c).

Interestingly, in the Forward model the RAD21 ChIP peak summit is skewed to the right when CTCF binding site is oriented towards the promoters’ side, whereas in the Reverse model it is skewed to the left when the CTCF binding site is oriented towards the enhancers’ side (Supplementary Fig. 10a–b). This suggested that the CTCF bound in the Forward orientation stalls cohesin translocated from the promoter side, and CTCF bound in the Reverse orientation stalls cohesin translocated from the enhancer side. This is indeed in line with previous findings that the N-terminus of CTCF protein directly interacts with the conserved essential surface (CES) of the RAD21-STAG2 subunits of the cohesin complex^24^.

Together, these findings suggest that while CTCF sites in either orientation affect the translocation of cohesin, the reverse orientation causes a more significant barrier to translocation than the forward orientation in definitive (fetal liver- and spleen-derived) erythroid cells.

### CTCF sites oriented towards the enhancers cause the strongest reduction in alpha-globin expression

**Fig. 5:**
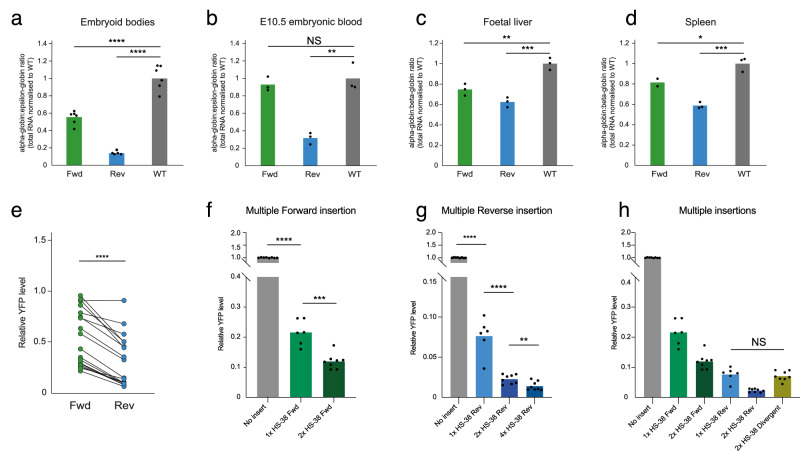
Expression of alpha-globin in CTCF insertion models in different erythroid tissues. Expression of alpha-globin normalised to embryonic beta-like globin, epsilon-globin (*Hbb-y*), **a** in EB-derived erythroid cells (WT and Fwd *n* = 6, Rev *n* = 5) and **b** E10.5 embryonic (*n* = 3 for all), and **c** alpha-globin expression normalised to adult beta-globin in cultured E12.5 fetal liver-derived Ter119+ erythroid cells (*n* = 3 for all), **d** and in spleen-derived Ter119+ erythroid cells (WT and Rev *n* = 3, Fwd *n* = 2). Bar plots show the mean ratio with individual data points marked by black dots. *P*-values were obtained using an unpaired two-tailed Student’s *t*-test. **e** Relative YFP levels normalised to neutral insert control in clones with different CTCF binding sequences, either in forward (Fwd) or reverse (Rev) orientations. *P*-value was obtained using a paired two-tailed *t*-test. **f** Relative YFP levels normalised to ‘No insert’ control in clones with multiple Forward CTCF insertions, **g** multiple Reverse CTCF insertions, and **h** multiple orientations of CTCF insertions as indicated on the plot. *P*-values were obtained using an unpaired two-sided Student’s *t*-test. Not significant (NS) *P* > 0.05, *P ≤ 0.05, **P < 0.01, ***P < 0.001, ****P < 0.0001. Exact *P*-values are provided in the Source Data file.

To assess if there is a difference in functional outcome between the Forward and Reverse models, we determined alpha-globin expression levels in various erythroid tissues using RT-qPCR. In EB-derived erythroid cells, alpha-globin expression was strongly reduced; expression was 44% lower in the Forward model compared to wild-type, and 86% lower in the Reverse model (Fig. 5a). We also determined levels of alpha-globin expression in E10.5 primitive embryonic erythroid cells, as these are the primary-tissue equivalents of EB-derived erythroid cells^31^. In this tissue, alpha-globin expression is not significantly altered in the Forward model, but in the Reverse model expression is reduced by 68% (Fig. 5b).

In two definitive erythroid tissues, namely fetal liver-derived and spleen-derived erythroid cells, the alpha-globin expression is significantly reduced both in the Forward and Reverse models, but again, the reverse orientation had the strongest effect on alpha-globin expression, with a 38% and 41% reduction respectively (Fig. 5c and d).

Furthermore, we noted that the ectopic site did not change the expression of zeta-globin (*Hba-x*; Supplementary Fig. 11), much like RAD21 depletion had not (Fig. 1c). This is in line with our previous observation that the expression of zeta-globin is less dependent on the alpha-globin enhancers^38^. Although expressed at low level, several non-erythroid genes that lie outside of the sub-TAD but still within the larger (165 kb) TAD show changes in expression level (Supplementary Fig. 12). Interestingly, the genes that increased in expression upon CTCF insertion, *Rhbdf1* and *Mpg*, were also affected when 5’ boundary CTCF sites were previously deleted^35^. Thus, the ectopic HS-38 CTCF seems to not only affect alpha-globin expression, but also has other, potentially secondary effects. It is possible that there is an element of enhancer-promoter competition between alpha-globin and the upstream genes revealed when the sub-TAD structure is disrupted.

Having tested alpha-globin expression in multiple CTCF insertion models and several different erythroid tissues, a striking difference between the Forward and Reverse models emerged. In all erythroid tissues tested, the Reverse model, in which the CTCF site predominantly blocks cohesin translocating from the enhancer side of the locus, had a stronger effect on alpha-globin expression. We wondered if this was a feature of this specific CTCF binding site, or of its position within the alpha-globin locus.

In a separate study^39^, we have developed a reporter assay based on the observation made here: that alpha-globin expression is highly sensitive to disruption by a single CTCF binding site (Fig. 5a–d). The reporter assay uses a YFP-tag at the alpha-gene to facilitate efficient evaluation of expression levels by FACS^31^ (Supplementary Fig. 13a). This assay showed a range of alpha-globin expression when different CTCF sites were inserted^39^.

As the additional 18 CTCF sites were introduced both in the forward and reverse orientations, the data allowed us to study the contribution of binding site orientation further. We observed the same trend as in the Forward and Reverse models described here: a CTCF site orientated towards the enhancers forms a stronger insulator than when it is orientated towards the genes (Fig. 5e). This shows that the orientation effect is not specific to the HS-38 binding site but is a general characteristic of the locus and the orientation of the CTCF sites relative to the enhancers and promoters.

### Insertion of multiple CTCF sites between the alpha-globin enhancers and the alpha-genes causes a further reduction in alpha-globin expression

Introduction of a single ectopic CTCF binding site, which blocks loop extrusion between the alpha-globin enhancers and genes, causes a strong reduction in alpha-globin expression, especially in cells derived from day 7 EBs. However, about 14% of alpha-globin expression remains in the Reverse model, and this is a substantial level of expression as alpha-globin is very highly expressed in wild-type erythroid cells. We therefore wondered if this reflected the limit of the contribution of loop extrusion to alpha-globin expression, or if it would be possible to reduce expression even further. It has been shown that a single CTCF binding site does not even block half of the translocating cohesin complexes^40^ and that additional CTCF sites can form a stronger boundary^41^.

We again employed the recently developed reporter assay^39^ to test if we could disrupt alpha-globin expression even further (Supplementary Fig. 13a). We designed several constructs, all inserting multiple copies of the same HS-38 CTCF binding site, spaced 500 bp apart (Supplementary Fig. 13b and 14a). When two CTCF binding sites in the forward orientation were inserted, the insulation was strengthened, illustrated by a lower YFP level compared to one forward CTCF binding site (Fig. 5f). Similarly, two reverse-oriented binding sites further reduced the level of expression. Although expression levels are already low in cells with two reverse-oriented binding sites, adding two more sites resulting in four reverse CTCF binding sites lowers alpha-globin expression further still (Fig. 5g). Together, the data suggests that insertion of multiple CTCF binding sites further reduced the alpha-globin gene expression by imposing further resistance to the extruding cohesin. We also assessed RAD21 binding in the multiple CTCF insertion model. ChIP-seq analysis revealed clear RAD21 enrichment at each inserted CTCF site, indicating that these sites act as independent cohesin accumulation points (Supplementary Fig. 14b). To enable precise assignment of reads, the inserted sequences were designed to include distinguishing SNPs, allowing us to resolve RAD21 peaks at individual insertion sites (Supplementary Fig. 14a). These data support a correlation between the number of inserted CTCF sites and local RAD21 accumulation, consistent with the model that additional CTCF sites impose increased resistance to cohesin-mediated loop extrusion.

Interestingly, introducing two CTCF binding sites in a divergent orientation (Fig. 5h) did not further increase the insulation when compared to a single CTCF site in the reverse orientation. This observation again suggested that CTCF site orientated towards the enhancers act as a stronger insulator than the CTCF site orientated towards the genes and is the major contributor of the insulation.

### 3D chromatin architecture changes upon CTCF insertion in an orientation-dependent manner

The presence of RAD21 and STAG2 at the ectopic CTCF binding site seems to indicate that this newly inserted site alters loop extrusion and thus 3D genome organisation. However, it has also been shown that CTCF and cohesin localisation is very similar at CTCF binding sites between the inactive alpha-globin locus in undifferentiated cells, and the active alpha-globin locus in erythroid cells^16,35^. Therefore, the 3D chromatin structure cannot be inferred from just the localisation of cohesin subunits and CTCF, and probing the 3D chromatin organisation can provide more information on how altering the translocation of cohesin may alter gene regulation at this locus.

We have previously shown by Capture-C^13^, MCC^16^ and by super-resolution imaging^15^ that the alpha-globin enhancers and promoters come into close juxtaposition within the erythroid specific sub-TAD both in primitive and definitive erythroid cells. As the sub-TAD forms, the convergent, flanking CTCF-elements are drawn into close proximity to each other. However, the flanking CTCF sites interact less frequently with the centre of the sub-TAD, thereby forming a loop structure encompassing the sub-TAD^35^.

**Fig. 6:**
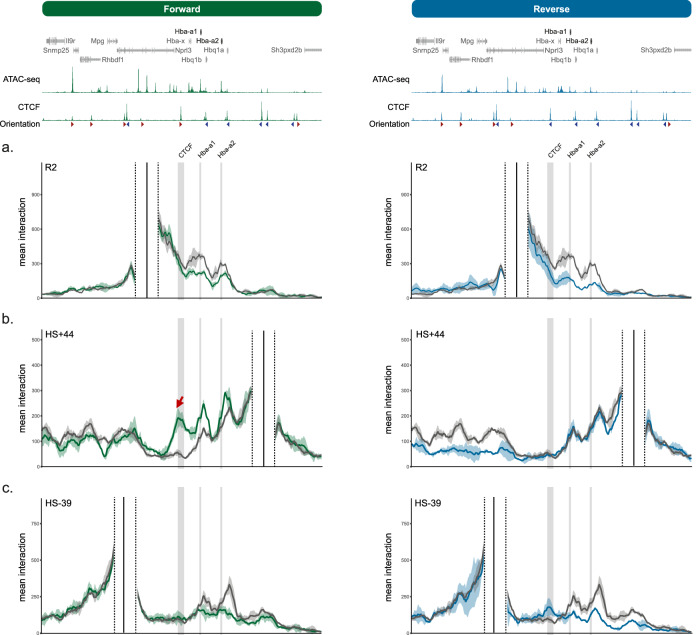
Changes in the three-dimensional genome organisation in the Forward and Reverse CTCF insertion models. Top tracks show ATAC-seq and ChIP-seq in the Forward and Reverse CTCF insertion EB models. Capture profiles from the viewpoint of **a** the alpha-globin enhancer R2, **b** CTCF binding sites HS + 44, and **c** HS-39. The profiles show normalised and averaged interaction frequencies across biological replicates, with shaded regions representing ±1 standard deviation (SD), for WT (grey, *n* = 3) Forward (green, *n* = 3) and Reverse (blue, *n* = 2) EB-derived erythroid cells. The position of the alpha-globin genes and the ectopic CTCF site are marked in grey. The exclusion zones around capture probes are marked by sashed black lines and the position of the viewpoint by a solid black line. The new peak of interaction between HS + 44 and the ectopic CTCF site is  indicated with a red arrow.

Using Capture-C, we investigated changes in the chromatin organisation upon introduction of the ectopic CTCF site in EB-derived erythroid cells. When the ectopic site was placed between the enhancers and promoters, in either orientation, interactions between these elements were reduced, when viewed from the main alpha-globin enhancer element R2 (Fig. 6a)^42^. Of particular interest, there was a stronger reduction in the frequency of enhancer-promoter interactions when the ectopic CTCF site was orientated towards the enhancers (Reverse model; Fig. 6a), where it should predominantly stall cohesin translocating from the direction of the enhancers to the alpha-globin genes. This is consistent with the changes we see in gene expression.

We next asked if the ectopic insertion altered the looped structure of the flanking CTCF sites, by viewing interactions from the flanking regions. In wild-type cells, the downstream CTCF binding site HS + 44 is found in close proximity to nearby CTCF binding sites at the promoters of the theta-globin pseudogenes (*Hbq1a* and *Hbq1b*) and to the upstream boundary elements (HS-38, HS-39, HS-59 and HS-71; Fig. 6b). When inserted in the forward orientation, the ectopic CTCF element is convergent with the downstream CTCF sites and is found in close proximity to them: when capturing interactions from HS + 44 we observed a new peak of interaction with the ectopic site (indicated with a red arrow, Fig. 6b). In addition, the interactions between the ectopic site and HS + 44 increase in the Forward model. In the Reverse model, there is no new peak with the ectopic CTCF binding site, nor is there an increase in other interactions. However, interactions across the locus with upstream CTCF binding sites are reduced (Fig. 6b).

Next, we examined interactions from one of these upstream binding sites, HS-39, which marks the 5’ boundary of the alpha-globin sub-TAD. In the Reverse model, there is a modest interaction peak between HS-39 and the ectopic CTCF binding site (Fig. 6c). At the same time, interactions between HS-39 site and CTCF sites downstream of the new site are reduced. In the Forward model, interactions with CTCF sites around the alpha-globin genes are reduced, potentially because these are now more frequently interacting with downstream binding sites, but otherwise most interactions remain intact (Fig. 6c).

Additionally, we have quantified and compared the RAD21 ChIP-seq peaks at  the CTCF binding sites in Forward and Reverse models (Supplementary Fig. 15a). RAD21 enrichment was significantly increased at the CTCF binding sites upstream of the ectopic CTCF binding site in the Reverse model over that in the Forward model. Alternately, increased RAD21 enrichment on HS + 44, the CTCF binding site downstream of the ectopic CTCF binding site, was observed in the Forward model over that in the Reverse model (Supplementary Fig. 15b). These observations support the Capture-C data, showing that ectopic insertion of the CTCF binding site altered cohesin-mediated loop extrusion and 3D genome organisation.

In summary, the ectopic CTCF site is found in close proximity in the Forward and Reverse models with flanking CTCF binding sites that are in convergent orientation, consistent with many previous observations^23,43,44^. It reduces enhancer-promoter interactions in both models and is associated with changes in the three-dimensional genome organisation in an orientation-dependent manner. Overall, these experiments fully support loop-extrusion as a molecular mechanism underlying enhancer-promoter interactions and cohesin as the effector, translocating primarily from the enhancer side of the locus, and intercepted by boundaries, in a position- and orientation-dependent manner.

## Discussion

Since long-range regulation was first reported, there have been many attempts to explain how enhancers communicate with promoters lying 10s-1000s kb away in the genome^29,45,46^. The first models included: tracking, in which a protein such as RNA Polymerase II might be loaded at enhancers and translocate along chromatin to reach and activate their associated promoters^47^; linking, in which chromatin associated factors arranged along the chromatin fibre might connect the enhancer and promoter^48,49^; relocation, in which enhancers and promoters migrate to pre-formed transcription factories^50^; and passive looping in which linked enhancers and promoters meet frequently by diffusion because they are tethered to each other. Once in contact, by whatever means, the enhancer and promoter might be found in a sub-nuclear structure with a high concentration of transcription factors and co-factors creating a favourable environment for transcription (as reviewed in refs. ^29, 51, 45, 46^).

All of these models have been tested experimentally, refined and elaborated upon. Most importantly, in the past decade it has been proposed that regulatory elements within TADs and sub-TADs may be brought into close proximity by the process of cohesin-mediated chromatin loop extrusion^1,2^. This process is delimited by specifically orientated CTCF binding sites that demarcate the active domain^23,44^. Although this is a very compelling and elegant model, experiments in which cohesin has been acutely degraded have been reported to show remarkably few changes in gene expression^8–11^ and consequently there remains an unresolved discussion about the role of loop extrusion in gene regulation^12,52,53^.

Here, we have investigated the role of cohesin and loop extrusion in the enhancer-mediated gene activation at the well-studied mouse alpha-globin locus. We found that the expression of alpha-globin is sensitive to cohesin degradation specifically at earlier stages of differentiation. We further explored the role of translocating cohesin by introducing an ectopic CTCF binding site between the alpha-globin enhancers and genes, which blocks translocating cohesin throughout erythroid differentiation. By introducing the binding site in two orientations, either posed to block cohesin coming from the direction of the enhancers or the promoters, we observed lower levels of alpha-globin expression in both models, but a stronger reduction when it predominantly blocks cohesin translocating from the direction of the enhancer. This coincides with higher levels of the cohesin subunits RAD21 and STAG2 at the ectopic site and a larger reduction in enhancer-promoter interactions.

Our observations that cohesin depletion does not affect alpha-globin expression late in erythroid differentiation resembles similar experiments in which few changes in gene expression were observed^9,11^. However, there are potentially confounding factors in the previous experiments that have not been fully explored and which have been further investigated here. First, most previous experiments have been conducted in steady-state conditions^3,12,54,55^. However, of interest, in experiments analysing the effect of cohesin depletion in primary macrophages in response to inflammatory stimuli, there was a large effect on inducible genes whereas expression of most constitutive genes was unchanged^32,56^.

During erythropoiesis, many genes are activated through enhancer-mediated mechanisms, with the globin loci providing a well-established example. In this study, cohesin depletion leads to reduced alpha- and beta-globin transcription specifically in early erythroid cells (day 5–6 EB-derived cells), while neighbouring widely expressed genes remain largely unaffected, indicating a selective requirement for cohesin in enhancer-dependent gene activation. Capture-C analysis reveals only modest changes in chromatin architecture at the alpha-globin locus, consistent with a partial disruption of enhancer-promoter interactions, particularly at early stages of differentiation. These observations suggest that cohesin plays a more prominent role in the establishment of regulatory interactions and not subsequent events required for regulatory architecture or expression. Also, in addition to its established role in loop extrusion, cohesin may contribute to gene regulation through effects on transcription factor occupancy or chromatin accessibility^57^ aspects we have not investigated in this work. Moreover, recent work where NIPBL, the cohesin loader, was acutely depleted, has shown that synergy between regulatory elements can buffer the loss of cohesin-mediated looping, allowing transcription to be maintained despite reduced chromatin interactions^58^. This provides a potential explanation for the relatively modest architectural changes and limited transcriptional effects observed upon cohsin depletion and especially in later stages of differentiation. At these later stages, enhancer-promoter communication may be re-established after cell division by cooperative interactions between regulatory elements or by alternative mechanisms such as protein–protein contacts^59,60^, or transcription may be maintained through re-initiation independently of continuous enhancer input. Together, these findings support a model in which cohesin is particularly important during early stages of lineage-specific gene activation, while later transcriptional maintenance becomes less dependent on cohesin-mediated chromatin architecture.

Another consideration is the degree to which cohesin is downregulated in these experiments. In some experiments, in contrast to the global loss of TADs and CTCF loops, in the absence of cohesin^9–11^, enhancer–promoter interactions are not completely abolished^61–64^. In line with these observations, it is of interest that two recent reports with Micro-C^57^ and a targeted MNase-based 3C^65^ analyses did not detect clear changes in enhancer–promoter interactions upon cohesin depletion in mESCs. It is possible that these different reports reflect the different degrees to which cohesin may have been depleted, which has previously been observed with similar chromatin-bound proteins such as CTCF^8,66^.

Based on a model whereby loop extrusion is halted by CTCF sites to create distinct interaction domains (TADs, sub-TADs, loops), we placed an ectopic CTCF binding site between the enhancers and target genes thereby blocking loop extrusion by an orthogonal approach. Similar to studies at the beta-globin locus^67,68^, we established that a single CTCF binding site inserted between the alpha-globin enhancer cluster and promoters was sufficient to significantly disrupt enhancer function. We harnessed this observation to examine how CTCF intercepted enhancer-promoter interactions, with specific focus on its impact on loop extrusion. Previous studies showed the necessity of inserting multiple CTCF sites in tandem to reach significant levels of insulation^41^. We therefore considered increasing the number of inserted CTCF sites and demonstrated an ability to modulate the interception of enhancer-promoter interaction by further intercepting cohesin progression and loop extrusion. It is worth noting that in our model, the insulation function partly reflects the insulator choice, with the alpha-globin HS-38 being a strong insulator. However, binding site sequence alone cannot adequately explain this. Several of the same CTCF binding site sequences that did not perform as strong insulators when inserted at the *Sox2* locus, did act as strong boundaries in our assay at the alpha-globin locus^39^. Other studies, such as that at the *Sox2* locus^69^, very little effect on gene expression when CTCF sites were inserted between the gene and its enhancers or found enhancer-promoter interactions and expression decoupled from CTCF-cohesin architectural role^58,69–72^. We therefore speculate that locus-specific characteristics, such as the dependence of a gene on its enhancer for a particular spatio-temporal expression, the concordance between enhancer-promoter interaction and expression, the distance between the enhancer and promoter, as well as the strength of the enhancers^73,74^ are some of the variables to consider when evaluating the effect of a boundary element or the requirement for loop extrusion for enhancer-promoter pairing and transcription^75^. Others have suggested that the role of loop extrusion is limited to very long-range enhancer promoter interactions^26,61,46^. We detect here a requirement for cohesin despite the relatively short ( ~ 30–50 kb) enhancer–promoter distances at the alpha- and beta-globin loci, demonstrating that loop extrusion can operate over modest genomic scales. Nonetheless, distance may influence the magnitude of this dependency, with very short-range interactions, such as *Hba-x* (<20 kb), showing reduced sensitivity to cohesin loss, which may account for the weaker phenotypic effects observed at this gene.

It is worth noting that an apparent discrepancy arises from the observation that RAD21 degradation at EB day 7 (late in differentiation) does not affect alpha-globin expression, whereas insertion of CTCF sites at the locus leads to a clear reduction in transcription. This raises the question of whether CTCF can function as an insulator independently of cohesin in this context. Based on differences in the timing and duration of cohesin perturbation between the experimental systems and all the literature in support of CTCF’s primary role in orchestrating loop-extrusion, we favour an alternative explanation. Acute RAD21 degradation at EB day 7 is transient (6 h) and occurs after key regulatory interactions and/or other mechanisms have been established, thereby limiting its impact on gene expression. In contrast, insertion of a CTCF site introduces a constitutive barrier to cohesin-mediated loop extrusion from the onset of differentiation, resulting in a more sustained disruption of enhancer–promoter communication. It is worth highlighting here that ChIP-seq analysis demonstrates RAD21 accumulation at each inserted CTCF site, with multiple insertions creating additional cohesin occupancy points, supporting the idea that increasing numbers of CTCF sites impose progressively greater resistance to loop extrusion. These observations are therefore consistent with a model in which CTCF acts by positioning and constraining cohesin, and in which the timing of perturbation is critical for its impact on gene regulation.

We also observed a striking difference between models with a forward or reverse oriented ectopic CTCF binding sites. The difference is most easily explained by a mechanism that involves tracking along the chromatin fibre and is therefore in accord with loop extrusion. Any mechanism not involving tracking along the chromatin fibre would be agnostic to the binding site orientation. Fundamentally, the Forward and Reverse models test the effect of blocking loop extrusion from different directions. Viewed in this light, the unequal decrease in alpha-globin expression between the Forward and Reverse CTCF sites indicates that loop extrusion progressing from the direction of the enhancers plays a more significant role in setting up enhancer-promoter interactions. It is possible that cohesin originating from the direction of the enhancers somehow plays a role in enhancer function, and therefore blocking extrusion from this direction has an especially strong effect on gene expression. It is also possible that cohesin extrusion is not equal in both directions along the chromatin fibre.

The data presented here are consistent with previous work suggesting that active enhancers may provide a loading site for cohesin which then translocates and thereby juxtaposes the enhancers to flanking promoters. Previous evidence of enhancers as sites of cohesin loading was based on the observations that more cohesin is present when an enhancer cluster is active^16^, or on the fact that cohesin levels decrease when a superenhancer is deleted^76^. However, these models cannot distinguish between cohesin loading at active loci, which include enhancers, promoters and gene bodies, and loading at enhancers specifically. Indeed, NIPBL, which is necessary for the loading of cohesin onto the chromatin fibre, is present at both active enhancers and active genes^16^, although there is some evidence that its presence at active genes could be a technical artefact^77^. There have also been reports of interactions between NIPBL and mediator^78,79^, which is present at the alpha-globin enhancers. Cohesin predominantly translocating from the enhancers also fits with our observations of higher levels of RAD21 at the reverse oriented binding site in definitive erythroid cells.

From current observations, it seems that the probability of an active distal enhancer interacting with the surrounding promoters will depend on many dynamic factors which will differ for different loci^29^. These currently include: location within a shared TAD and particularly within a shared sub-TAD^3^; the distance between the enhancer and promoter^73^; the “strength” of the enhancer^73,80,81^; the “compatibility” between enhancer and promoter^70,82,83^; competition between surrounding promoters^84^; and the homotypic and heterotypic interactions between proteins which contribute to the interacting elements and the transcriptional hub^48^. Factors with architectural roles other than cohesin have also been studied and shown to impact enhancer-promoter interactions and gene expression^37,57,85–90^. The data presented here and elsewhere, are consistent with a model in which the degree of cohesin-mediated loop extrusion and the direction of translocation also play a significant role in establishing enhancer-promoter interactions especially during differentiation or in response to stimuli rather than in the steady state^32^.

We conclude that the data presented here are consistent with a model in which the alpha-globin enhancers and promoters are brought into proximity by a tracking mechanism involving cohesin-mediated loop extrusion and will contribute to the hypothesis that enhancers specifically play a role in directing loop extrusion and gene expression.

## Methods

### Cell and genome editing

E14Tg2a mouse embryonic stem cells (mESCs) were used for all mESC experiments in this study. The cells were maintained in KnockOut™ DMEM or Glasgow’s Minimal Essential Medium supplemented with 10% fetal bovine serum, 1 mM sodium pyruvate, 2 mM L-Glutamine, 1x non-essential amino acids (all Gibco), beta-mercaptoethanol, and Leukemia Inhibiting Factor (LIF). During gene targeting, the media was supplemented with penicillin and streptomycin (Gibco) depending on required selection markers. Cells were usually grown on 0.1% (v/v) gelatine (Sigma-Aldrich: G1393), except during gene targeting for the CTCF insertion mouse model and prior to blastocyst injections, when they were grown on mitotically inactivated mouse embryonic fibroblasts (MEFs).

For the CTCF insertion cells (Forward/Reverse model) and mouse model, a double nickase CRISPR approach was used. Single guide-RNA plasmids were generated by cloning oligonucleotides to the target site (see Supplementary Table 1) into pX335 (Addgene plasmid no. 42335^91^). pX335 had been modified to contain a puromycin selection cassette. The homology-directed repair (HDR) plasmid was prepared by amplifying 1.4 and 1.2 kb homology arms from genomic DNA, which were then cloned into the pJET vector (CloneJET PCR cloning kit, Thermo Scientific). A synthetic DNA fragment (Genewiz) containing the 82 bp CTCF binding site, flanked by heterospecific lox sites (loxP and lox2272^92^), two FRT sites and flanking Rox sites was cloned between the homology arms (see Supplementary Table 2). A neomycin selection cassette was cloned between the FRT sites and the sequence of the final plasmid was confirmed by Sanger sequencing.

Wild-type mESCs were electroporated using the Neon transfection system (Invitrogen) using 3 × 1400 V for 10 ms with 2.5 μg of the two sgRNA nickase-Cas9 plasmids and 5 μg of the targeting vector DNA. The transfected mESCs were plated at low density and selected with 600 ng/ml puromycin for two days to select for the gRNA transfected cells, followed by 350 ng/ml G418 selection for 5 days to select for mESCs with successful integration events. Individual targeted clones were picked, expanded and initially screened for correct integration using homology-arm spanning PCRs (Immolase DNA polymerase, Bioline, supplemented with Q solution, Qiagen). Genotypes of positive clones were confirmed by Sanger sequencing. Three clones with the correct insertion were transiently transfected with either 5 μg pFlpO (Addgene #13793) to remove the neomycin selection cassette or both 5 μg pFlpO (Addgene #13792) and 5 µg of pCrePac^93^ to simultaneously remove the selection cassette and invert the CTCF binding site. This provided clones with the forward and reverse orientated CTCF site in triplicate.

For the CTCF activity reporter system, genetically modified mESC models were generated using the double nickase HDR strategy. In brief, the two single guide-RNAs targeting the insertion site were individually cloned into the pX335 vector containing puromycin selection cassette as described above. To generate the HDR donor plasmid containing the inserted CTCF elements, oligonucleotides corresponding to each CTCF element were cloned into the pROSA-TV2 vector (created by Prof. Benjamin Davies group) between the pair of BsaI sites with the NEB Golden Gate Assembly Kit (NEB #E1601). The pROSA-TV2 was designed to contain the 1.4 and 1.2 homology arms of the inserted site and a hygromycin selection cassette that is flanked by a pair of lox sites (loxP).

Synthetic cassettes containing two to four copies of the 68 bp HS-38R CTCF-binding sequence in various orientation were designed for inserting into the CTCF activity reporter system. To preserve spacing between adjacent CTCF motifs and minimise interference with CTCF binding, the individual HS-38R binding sites were separated by a 500 bp spacer fragments derived from the endogenous alpha-globin locus, using sequences from either the interval between HS-38 and HS-39 or the region upstream of HS-39. To enable unambiguous assignment of sequencing reads to inserted sequence rather than the endogenous locus, nine SPRET-specific SNPs were incorporated into the spacer regions. In addition, four point mutations disrupting BsaI recognition sites were introduced to facilitate Golden Gate assembly and to further distinguish the synthetic cassette from the endogenous sequence. Sequences of the CTCF element oligonucleotides are listed in the Supplementary Table 3.

The E14TG2a mESCs used for the CTCF activity reporter assays were modified by hemizygously deleted one wild-type copy of the alpha-globin locus to facilitate further genetical modifications. In addition, a yellow fluorescent protein (YFP) was tagged to the *Hba-a1* gene, so that the YFP level is used as a readout for the *Hba-a1* expression level, as reported previously^31^. The mESCs were co-transfected with 1.66 μg of the two sgRNA nickase-Cas9 plasmids and 0.83 μg of the HDR plasmids using the Lipofectamine LTX and Plus Reagent (Invitrogen #15338-100). Transfected cells were first selected by 1 μg/ml puromycin for 2 days and subsequently selected by 250 μg/ml hygromycin for 6 days. The drug-selected cells were then transfected with the Cre-recombinase vector to remove the integrated hygromycin-resistance cassette. Genetically modified mESCs were FACS sorted into 96-well plates and grown into individual colonies. The colonies were screened for the correct integration by homology-arm spanning PCRs and the genotypes were further confirmed by Sanger sequencing.

For the RAD21 acute depletion experiments, the native *Rad21* gene was tagged with FKBP12^F36V^^30^ by CRISPR-Cas9 mediated HDR in wild-type mESCs. To achieve this, the sgRNA (see Supplementary Table 1) was cloned into pX458 (Addgene plasmid no 48138^94^), which encodes Cas9 and enhanced green fluorescent protein (EGFP). HDR templates containing the FKPB12^F36V^ tag were synthesised (see Supplementary Table 2) and cloned by GeneArt (Invitrogen) into double-stranded plasmid vectors (pMX).

Mouse ESCs were transfected using Lipofectamine LTX Reagent with PLUS Reagent (Invitrogen), performed according to manufacturer’s instructions: 5 μl LTX reagent, a total of 2.5 μg purified plasmid DNA (1:3 ratio of sgRNA/Cas9 plasmid DNA:donor plasmid DNA), 2 μl PLUS reagent, and 250 μl Opti-MEM (Gibco). After 24 h, transfected cells were rinsed with PBS and given fresh media. Transfected cells were selected based on the presence of EGFP by FACS after 48 h and positive cells were single cell sorted into gelatine coated 96 well tissue culture plates. Following 10 days of growth, surviving colonies were split across replica plates and screened for positive clones by PCR.

### Animal procedure

The mutant mouse strains reported in this study were backcrossed and maintained on a C57BL/6 J background. Protocols were approved through the Oxford University Local Ethical Review process. Experimental procedures were in accordance with the European Union Directive 2010/63/EU and/or the UK Animals (Scientific Procedures) Act (1986), and reviewed by the clinical medicine Animal Welfare and Ethical Review Body (AWERB). Experimental procedures were conducted under project licences PPL 30/2966 and PPL 30/3339.

Mouse ESCs with the original insertion (as described above), prior to Flp and Cre recombinase treatment, were used for blastocyst injection. After germline transmission, these were crossed with a Flp-expressing mouse line (B6.Cg-Tg(ACTFLPe)9205Dym/J; Jackson Laboratory stock #005703), and following positive genotyping, with a Cre-expressing line^95^ to produce both a forward and reverse CTCF insertion strains.

### Fetal liver culture

The in vitro mouse fetal liver culturing system is based on previous protocol^96,97^. Briefly, E12.5 mouse fetal livers were dissected and cultured in StemPro medium (Invitrogen) supplemented with Epo (1 U/mL; Janssen, PL 00242/029), SCF (50 ng/mL; Peprotech, 250-03), dexamethasone (1 μM; Hameln, DEXA3.3) and 1× L-Glutamine (Invitrogen) for 6–8 days to expand the erythroid progenitor population. During culture, the erythroblast cell count was maintained at 1 × 10^6^ cells/ml or under. If necessary cells were frozen at day 4 and after thawing, expanded for a further 2–4 days.

### In vitro embryoid body formation and differentiation

An in vitro haematopoietic differentiation protocol^31^ was used to differentiate mESCs into embryoid bodies (EBs), based on a previously published protocol^98^. In brief, proliferating mESCs were cultured in IMDM-ES media (Iscove’s Modified Dulbecco Medium (IMDM) (Gibco) supplemented with 15% fetal bovine serum (FBS), 100 U/ml penicillin/streptomycin, 1000 U/ml LIF, and 1.5 × 10^−4 ^M monothioglycerol (MTG; Sigma-Aldrich: M6145)) for 24 to 48 h prior to primary plating. Subsequently, cells were plated in 10 ml differentiation media in bacterial petri dishes (15,000–50,000 cells/plate) and cultured for 7 days. Differentiation medium consisted of IMDM media supplemented with 15% heat-inactivated FBS (1 h at 56 °C), 2 mM L-Glutamine, 300 μg/ml transferrin (Roche), 50 μg/ml ascorbic acid, 3 × 10^-4 ^M MTG and 5% Protein-Free Hybridoma Media II (Gibco).

### Cellular phenotyping the cells upon EB disaggregation

After 5, 6 or 7 days of differentiation, EBs were disaggregated using 0.25% trypsin (Gibco), washed with 10% FBS in PBS, and resuspended in 100 μl of 10% FBS in PBS with erythroid-specific surface markers: 2.5 ug/ml working concentration of FITC Rat anti-mouse CD71 antibody (BD Pharmingen or eBioscience) and 1ug/ml working concentration of PE anti-mouse Ter119 (BD Pharmingen) per 10^6^ cells for 20 min at 4 °C. After washing in 10% FBS in PBS, cells were analysed by fluorescent-activated cell sorting (FACS; Attune Nxt Flow Cytometer, Thermo Fisher). Same cell populations were assessed for general red blood cell morphology after automated Wright–Giemsa staining (Bayer HealthCare, Mematek®).

### Isolation of erythroid cells

After 5, 6 or 7 days of differentiation, EBs were disaggregated using 0.25% trypsin (Gibco) and stained with 0.5 μg FITC Rat anti-mouse CD71 antibody (BD Pharmingen or eBioscience) per 10^6^ cells for 20 minutes at 4 °C, followed by washing and incubation with anti-FITC MACS microbeads (Miltenyi Biotec) for 15 min at 4 °C. Stained cells were then purified using MACS lineage selection columns (Miltenyi Biotec) and immediately processed for various assays or treated with dTAG. For dTAG treatment, CD71+ cells were plated in differentiation media, as described above, supplemented with 1U/ml Epo (Janssen, PL 00242/029), and 500 nM dTAG-13 for up to 6 h for the treated cells. Control cells were incubated for the same amount of time as the treated cells. The purity of the isolated erythroid population was verified by fluorescent-activated cell sorting (FACS; Attune Nxt Flow Cytometer (Thermo Fisher)).

After 6 to 8 days of expansion, fetal liver cells were stained with 1 μg anti-mouse Ter119-PE antibody (BD Pharmingen) per 10^6^ cells for 20 min at 4 °C. Cells were washed with 10% FBS in PBS and resuspended in 135 μl cold PBE buffer (PBS with 0.5%BSA and 2 mM EDTA) with 15 μl Ter119-PE microbeads (Miltenyi Biotec) per 10^6^ cells, and incubated for 15 min at 4 °C. Erythroid cells were then isolated using MACS lineage selection columns (Miltenyi Biotec) and processed for various assays. Purity of isolated populations was assessed as described above.

Primary erythroid cells were obtained from the spleen of adult mice treated with phenylhydrazine, as described previously^99^. Spleens were mechanically disrupted in 10% FBS in PBS to produce a single cell suspension. Cells were resuspended in 1 ml 10% FBS in PBS and stained with 6 μg anti-mouse Ter119-PE antibody (Miltenyi Biotec) per 10^7^ cells for 20 minutes at 4 °C. After washing with 10% FBS in PBS, cells were resuspended in 80 μl cold PBE buffer (PBS with 0.5% BSA and 2 mM EDTA) and 20 μl anti-PE microbeads (Miltenyi Biotec) per 10^6^ cells and incubated for 15 minutes at 4 °C. Ter119+ cells were selected with MACS lineage specification columns (Miltenyi Biotec), typically using one column for up to 5 × 10^7^ cells, and processed for various assays Purity of isolated populations was assessed as described above.

Replicates were obtained via separate EB differentiations of different clones or from separate animals.

### Western blot

Total protein from 500,000 EB-derived CD71+ cells were harvested for western blotting. Cells were washed in ice-cold PBS twice and lysed in RIPA buffer (Sigma #SLCG2240) supplemented with 10% protease inhibitor cocktail (Roche, 11873580001) for 30 min at 4 °C. Protein lysates were quantified using Qubit Protein Assay Kit (Invitrogen #Q33211) and mixed with the western blot denaturing loading dye (125 mM Tris-HCl, 4% SDS, 50% Glycerol, and 0.4 g Orange G) at a 1:10 ratio. DTT (Sigma #43816) was added at a final concentration of 100 mM and the samples were denatured at 95 °C for 10 min. 40 μg of protein were loaded and run on pre-casted 4–12% NuPAGE Bis-Tris gels (Invitrogen #NP0335) for 90 min at 120 V. Samples were transferred on nitrocellulose membrane (Amersham GE Healthcare #10600008) for 90 min at 30 V. The membrane was blocked in 5% milk in PBS for 1 hour at room temperature with constant agitation. The membrane was incubated with primary antibodies at 4 °C overnight. The dilution for the mouse anti-FLAG antibody (Sigma, #F1804) is 1:1000 and the dilution for the rabbit anti-Beta actin antibody (Abcam #ab115777) is 1:200 in blocking buffer with 0.2% Tween-20. The membrane was washed three times with PBS with 0.1% Tween-20 and was incubated with secondary antibodies for 1 h in dark. The dilutions of the near-infrared coupled secondary antibodies IRDye 800CW goat anti-rabbit IgG (Abcam, #ab216773) and IRDye 680RD goat anti-mouse IgG (Abcam #ab216776) are 1:1000 in blocking buffer with 0.2% Tween-20. The membrane was washed three times with PBS-0.1% Tween-20 before detection with the iBright FL1000 scanner and software (Invitrogen) on the 680 nm and 800 nm channel. The quantification of the protein bands on the western blot was performed by using the Image J package and the result reflects the amount of the FLAG-RAD21 protein relative to the Beta-actin loading control as a ratio.

### Expression analysis

Total RNA was extracted from 5 × 10^5^ – 5 × 10^6^ cells. Cells were lysed in TRI Reagent (Sigma-Aldrich) according to manufacturer’s instruction and immediately stored at −80 °C. Total RNA was isolated either by using chloroform extraction or using a Direct-zol MiniPrep or MicroPrep kit (Zymo Research).

For chloroform extraction, chloroform was added to the sample in TRI reagent in a ratio 1:5 (v/v) and the sample was mixed vigorously, followed by room temperature incubation. Then the sample was centrifuged (20,000 × *g*, 4 °C) for 10 min and the aqueous layer was transferred and precipitated with isopropanol and glycoblue (Thermo Fisher) at room temperature for a few minutes. The samples were centrifuged (20,000 × *g*, 4 °C, 10 min) and the pellet containing the RNA was washed with 70% ethanol, air dried and resuspended in DNAse/RNase-free water. Samples were then treated with DNase using the TURBO DNA-free kit (Invitrogen) according to manufacturer’s instruction. Briefly, samples were treated with DNase enzyme for 30 min followed by inactivation for 5 min, after which the samples were centrifuged and the supernatant was used for cDNA synthesis after quality control steps. RNA isolation using the Direct-zol kits was performed according to manufacturer’s instructions, but the DNase I treatment was extended to 30 min at room temperature (rather than 15 min). The quality of all isolated RNA was assessed using RNA Analysis Screentape (Tapestation, Agilent Technologies) and only samples with high ( > 7.5) RNA integrity number (RIN) scores were used in subsequent steps.

Up to 1 μg RNA was used for cDNA synthesis using the Superscript III Reverse Transcriptase kit (Invitrogen) following manufacturer’s instructions. For each sample, a control without reverse transcriptase enzyme was included to account for any incomplete genomic DNA digestion. cDNA was diluted 5x before qPCR analysis to determine the relative changes in gene expression. qPCR analysis was performed in triplicate using Fast SYBR green master mix (Applied Biosystems) using primers listed in Supplementary Table 1. The ΔΔCt-method was used for relative quantification of RNA abundance.

### Digital Droplet PCR

In brief, cDNA was synthesized by using 200 ng of RNA with the Superscript III Reverse Transcriptase Kit (Invitrogen) according to manufacturer’s protocol. cDNA was diluted 100x for *Hba* expression analysis, or 10x for *Hba-N*, *Hbb*, and *Hbb-N* expression analysis. ddPCR reaction was set up in triplicate using the 2x ddPCR Supermix for Probes (No dUTP) (BioRAD), 20x TaqMan® target probe mix (FAM) (Applied Biosystems), and 20x TaqMan® Gapdh probe mix (VIC) (Applied Biosystems) to a total volume of 22ul. Droplet generation was performed using the automated droplet generator (AutoDG^TM^) (Bio-Rad). Thermal cycling of the droplets was performed using a C1000 Touch Thermal Cycler (Bio-Rad). The droplets were read on QX200 Droplet reader (Bio-Rad) and analysed with the QuantaSoft Analysis Pro software (Bio-Rad). Final ddPCR data were presented as log2(target gene concentration/Gapdh concentration).

### Single molecule RNA FISH

Single-molecule RNA FISH (smFISH) was performed as previously described^33^. A set of 34 oligonucleotide probes tiling the *Hba-a1/2* nascent transcript (Supplementary Table 4) was designed using Stellaris probe designer and conjugated with Quasar 570 dye (Biosearch Technologies). Mouse ESCs were differentiated into EBs and harvested at days 5, 6, and 7 of differentiation, and the erythroid populations were purified as described above. The cells were further treated with 500 nM dTAG or no treatment for 6 hours as described above. After the treatment, 2 × 10^5^ cells were adhered onto poly-L-Lysine-coated 12 mm #1.5H round precision coverglasses (Thorlabs), for 5 min at room temperature and then fixed in 3.7% (wt/vol) formaldehyde for 20 min at room temperature. The coverslips were subsequently washed in PBS twice and then permeabilized with 70% ethanol and stored at 4 °C for a maximum of 1 month. For the probe hybridization, the cells were first rehydrated in 0.2× SSC with 10% (vol/vol) formamide and were incubated with the labelled probes at a final concentration of 125 nM in hybridisation buffer (2× SSC, 25% Formamide (vol/vol), 5x Denhardt’s solution, 200 ng/µl Salmon sperm DNA, 1 mM EDTA, 50 mM NaPO4) in a humid chamber at 30 °C overnight. The cells were washed twice in wash buffer and once in wash buffer with DAPI 0.5 µg/mL for 30 min at 30 °C. Coverslips were then mounted in Prolong Diamond Antifade Mountant (ThermoFisher Scientific) overnight at room temperature, in the dark. The cells were imaged with a Zeiss Cell Observer inverted microscope with Yokogawa CSU-X1M 5000 spinning disk unit, Plan-Apochromat 63x/1.40 NA oil objective, and Hamamatsu Orca Flash 4.0 sCMOS detector. DAPI was excited with the 405 nm laser with 50 ms exposure, 10% laser power (50 mW) and collected with a 450/50 bandpass emission filter. *Hba-a1/2* Quasar 570 was excited with 561 nm laser with 300 ms exposure, 25% laser power (40 mW) and collected with a 629/60 bandpass emission filter. Z-stacks of 55 slices were collected, with a voxel size of 86 × 86 × 240 nm.

### YFP-reporter assay data acquisition

A detailed description of the reporter assay has been published^45^. Briefly, differentiated EB cells were disaggregated and subjected to staining using the anti-mouse CD71-APC antibody (eBioscience, 11-0711-80) at a concentration of 1:8000 in staining buffer, along with Hoechst (Invitrogen, 33258) at a concentration of 1:10000 in staining buffer. The cells were stained for 30 min at 4 °C in the dark. The YFP signal level of live CD71+ cells was quantified and utilized as a proxy measure for alpha-globin gene expression in CD71+ cells. FACS Analysis was conducted using the Attune NxT Flow cytometer (Thermo Fisher) and the Attune NxT software package V4.2.0.

### ATAC-seq

Assay for transposase-accessible chromatin (ATAC)-seq was performed as previously described^100^. Briefly, 60,000–75,000 cells were lysed in cold lysis buffer (10 mM Tris HCl, 10 mM NaCl, 3.4 mM MgCl_2_, 0.1% IGEPAL CA-630) to extract nuclei. Pelleted nuclei were incubated in transposase reaction mix (1 X TD reaction buffer, 2.5 µl Tn5 transposase (Nextera, Illumina)) for 30 min at 37 °C. Tagmented DNA was purified using a MinElute PCR purification kit (Qiagen) and amplified and indexed with NEBNext High-Fidelity 2X PCR Master Mix (NEB) and custom primers. Amplified libraries were purified with PCR cleanup kit (Qiagen) and library quality was assessed on a Tapestation (Agilent Technologies). Libraries were sequenced on the Illumina NextSeq platform using a 75-cycle paired end kit (NextSeq 500/550 High Output Kit).

### ChIP-seq

A Chromatin Immunoprecipitation (ChIP) Assay Kit (Merck Millipore 17-295) was used for all CTCF ChIP experiments following manufacturer’s instructions. First, chromatin of 5–10 × 10^6^ isolated erythroid cells was fixed in 1% formaldehyde for 10 min. The crosslinking reaction was quenched with 125 mM glycine for 5 min. Chromatin was sonicated using the Bioruptor or Bioruptor pico (Diagenode) to produce fragments of 200–500 bp. Sonicated chromatin was incubated with 10 µl anti-CTCF antibody per sample (Millipore 07-729) at 4 °C on a rotator overnight. Protein A agarose beads were added and incubated with the chromatin for 60 min at 4 °C with rotation, and beads were sequentially washed and eluted following manufacturer’s instructions.

For RAD21 and STAG2 ChIP, samples were either processed using the Millipore kit as described previously^35^ or using a magnetic-bead based protocol. Briefly, 10^7^ cells were fixed with 2 mM disuccinimidyl glutarate (DSG; Thermo Fisher) for 50 min, followed by 1% formaldehyde for 10 min. The crosslinking reaction was quenched with a final concentration of 125 mM glycine, for 5 min. After cell lysis, chromatin was sonicated using the Bioruptor (Diagenode), Bioruptor pico (Diagenode) or ME220 Focused-ultrasonicator (Covaris) to produce fragments of 200–500 bp. Sonicated chromatin was incubated with 10 µl RAD21 antibody (Abcam, #ab154769) or STAG2 antibody (Bethyl #A302-580A) per sample at 4 °C on a rotator overnight. Samples were then treated as CTCF ChIPs described above. Alternatively, using a magnetic bead-based protocol, samples were fixed with 1% formaldehyde for 10 min at room temperature and quenched with 125 mM glycine for 5 min. Cells were lysed in SDS lysis buffer (1% SDS, 10 mM EDTA, 50 mM Tris-HCl pH 8) supplemented with Proteinase Inhibitor Cocktail (Roche, 11697498001) and chromatin was sonicated using the Bioruptor pico (Diagenode) to produce fragments of 200–500 bp. Chromatin was isolated using a RAD21 antibody (ab154769) and Protein-G Dynabeads (Thermo Fisher) and washed three times with RIPA buffer (50 mM HEPES-KOH, pH 7.6, 500 mM LiCl, 1 mM EDTA, 1% Igepal CA-630, 0.7% sodium deoxycholate) and two times with Tris-EDTA (TE buffer with 50 mM NaCl). Precipitated chromatin was eluted in 1% SDS, 10 mM EDTA and 50 mM Tris-HCl. After decrosslinking and treatment with RNase A and proteinase K, DNA was purified using DNA Clean-up and Concentration kit (Zymo Research).

Quality checked and approved libraries, as determined by qPCR enrichment, were prepared for sequencing with an Ultra II Library Prep Kit (NEB) and sequenced on the Illumina NextSeq platform using a 75-cycle paired end kit (NextSeq 500/550 High Output Kit).

### Capture-C

Capture-C was performed as previously described, either following the next-generation Capture-C protocol or the NuCapture-C protocol^101^. A total of 3–10 × 10^6^ EB-derived CD71+ were processed per biological replicate and quality-checked and approved libraries were indexed using NEBNext Ultra II DNA Library Prep Kit for Illumina (New England Biolabs: E7645) according to the manufacturer’s instructions. We performed capture enrichment using biotinylated oligos previously published (HS-38)^35^ or listed below (*Hba-a1* promoter, R2, and HS44; Supplementary Table 5). Data was analysed using scripts available at https://github.com/Hughes-Genome-Group/CCseqBasicS. Data visualisation and statistical analysis was performed using CaptureSee^102^.

### ATAC-seq and ChIP-seq data analysis

Next-generation sequencing reads were trimmed using trimgalore (version 0.3.1 https://www.bioinformatics.babraham.ac.uk/projects/trim_galore/) with stringency 2 and aligned with bowtie2^103^ (version 2.1.0) to a custom genome based on mm9, edited to include CTCF site insertion as appropriate. Samtools^104^ (version 1.3) was used to remove PCR duplicates and select reads from chromosome 11, and bedgraphs were created using bamCoverage (part of deeptools version 2.2.2^105^), with normalisation set to RPKM. Replicates were merged with unionbedg (bedtools version 2.25.0^106^) and bigwig tracks were created with bedGraphToBigWig (deeptools). To compare bigwig files from different genotypes, tracks were shifted to align over genes and other genomic features. Finally, data was visualised using the UCSC genome browser^107^.

### Read count analysis and peak calling

Reads were counted over the ectopic CTCF site (chr11:32,171,303-32,172,502) for every sample (aligned to CTCFins custom genome). For normalisation, peaks were called on a subset of ChIP samples with Lanceotron^108^, using model wide-and-deep_jan-2021, on bigWig files generated using bamCoverage (part of deeptools 3.4.3^105^) with extendReads (default), binsize 1 and normalisation set to RPKM, after aligning to mm9. Peaks with a peak score between 0.8 and 1 were used for further analysis. From these peak calls, peaks present in multiple samples were used to create a peak list. Next, bedtools multicov (bedtools v2.29.2) was used to count reads over the peak call list for every sample and the average number of reads per peak was used to normalise read counts over the ectopic CTCF site.

### Statistics and reproducibility

For ChIP-seq, ATAC-seq, and NG Capture-C data, at least 2 independent replicates of each genotype were analysed. For RT-PCR, ddPCR, and smFISH experiments at least three independent replicates of each treatment group or genotype were analysed to allow statistical analysis (Student’s *t*-test).

Randomisation was not applicable to this study as comparison was between distinct genotypes or treatment groups. Blinding was applied to quantify the number of active foci in the smFISH analysis. Image file name was masked prior to analysis, counts were made by an assessor blinded to treatment group allocation. Overall, no data were excluded from the analyses.

### Reporting summary

Further information on research design is available in the Nature Portfolio Reporting Summary linked to this article.

## Supplementary information

Supplementary Information

Transparent Peer Review file

Reporting Summary

## Source data

Source Data

## Data Availability

The sequencing data generated in this study have been deposited in the NCBI Gene Expression Omnibus under accession number GSE163012. Wildtype CTCF ChIP-seq and ATAC-seq data in EBs^31^ and wildtype RAD21 ChIP-seq data in spleen^35^ have been previously published under accession number GSE184435 and GSE97871. Microscopy images of the smFISH experiment have been deposited in the BioImage Archive under accession number S-BIAD3197. The processed gene expression data generated in this study are provided in the Supplementary Information and Source Data file. Source data are provided with this paper.
