# Supplementary material for: Loop extrusion by cohesin plays a role in enhancer-activated gene expression early in differentiation
Source: Nat Commun. 2026 May 26;17:7297. doi: 10.1038/s41467-026-73049-5 (PMC13402345; doi:10.1038/s41467-026-73049-5)
Supplement: Supplementary file 1 — Supplementary Information [file 41467_2026_73049_MOESM1_ESM.pdf]

Supplementary Figure 1: Acute depletion of RAD21-FKBP-tagged CD71+ erythroid cells derived from day 6 EBs.

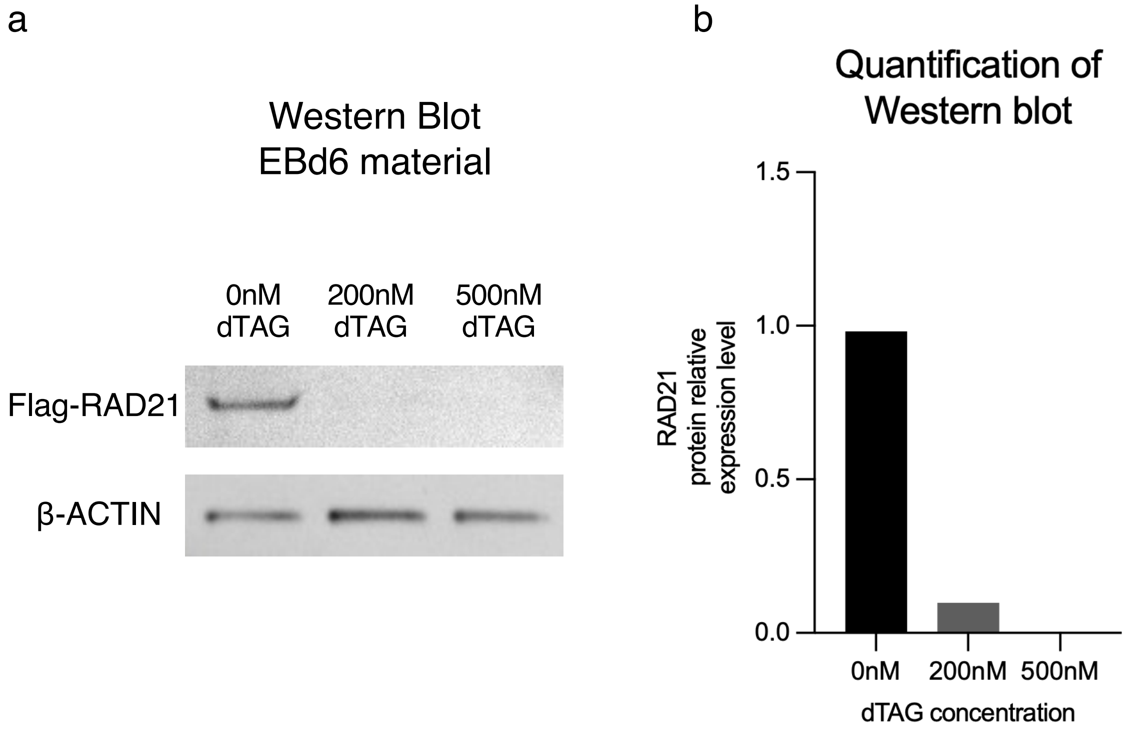

(a) Western blot showing the effect of 0nM, 200nM, and 500nM of dTAG-13 (dTAG) treatment for 6 hours on the Flag-tagged RAD21-FKBP (Flag-RAD21) protein and using β-ACTIN as a control. **(b)** A semi-quantitative measure of the Western blot signal by ImageJ shows the complete depletion of RAD21 protein when the cells were treated with 500nM dTAG for 6 hours.

Supplementary Figure 2: The genome-wide loss of RAD21 binding on chromatin upon acute depletion of RAD21.

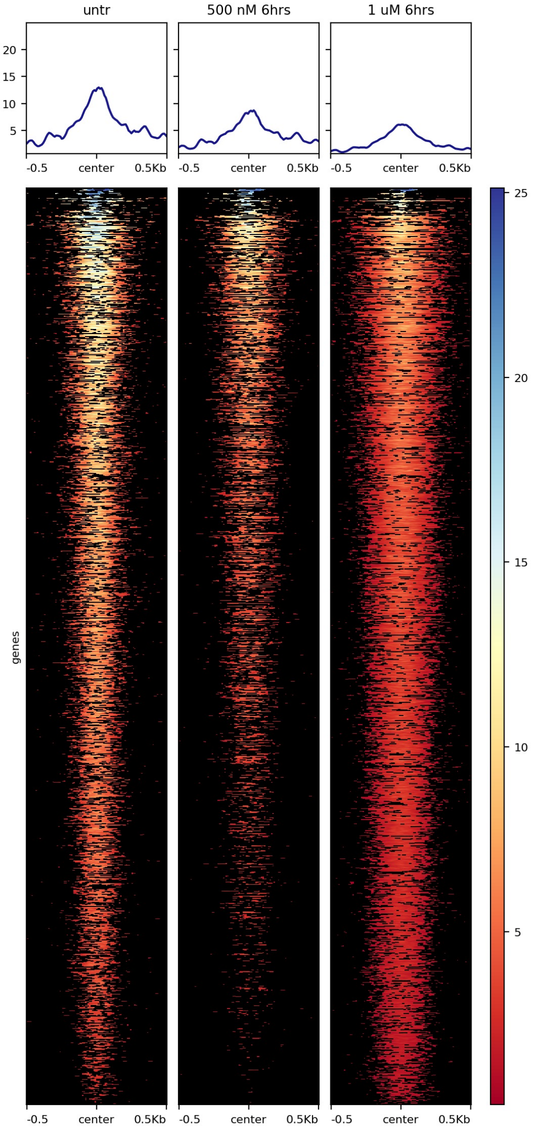

Meta-analysis of the RAD21 ChIP-seq showing the loss of RAD21 binding to chromatin genome-wide upon acute depletion of RAD21 in CD71+ erythroid cells derived from day 7 EBs.

Supplementary Figure 3: Gene expression in EB-derived CD71+ erythroid cells upon acute depletion of RAD21.

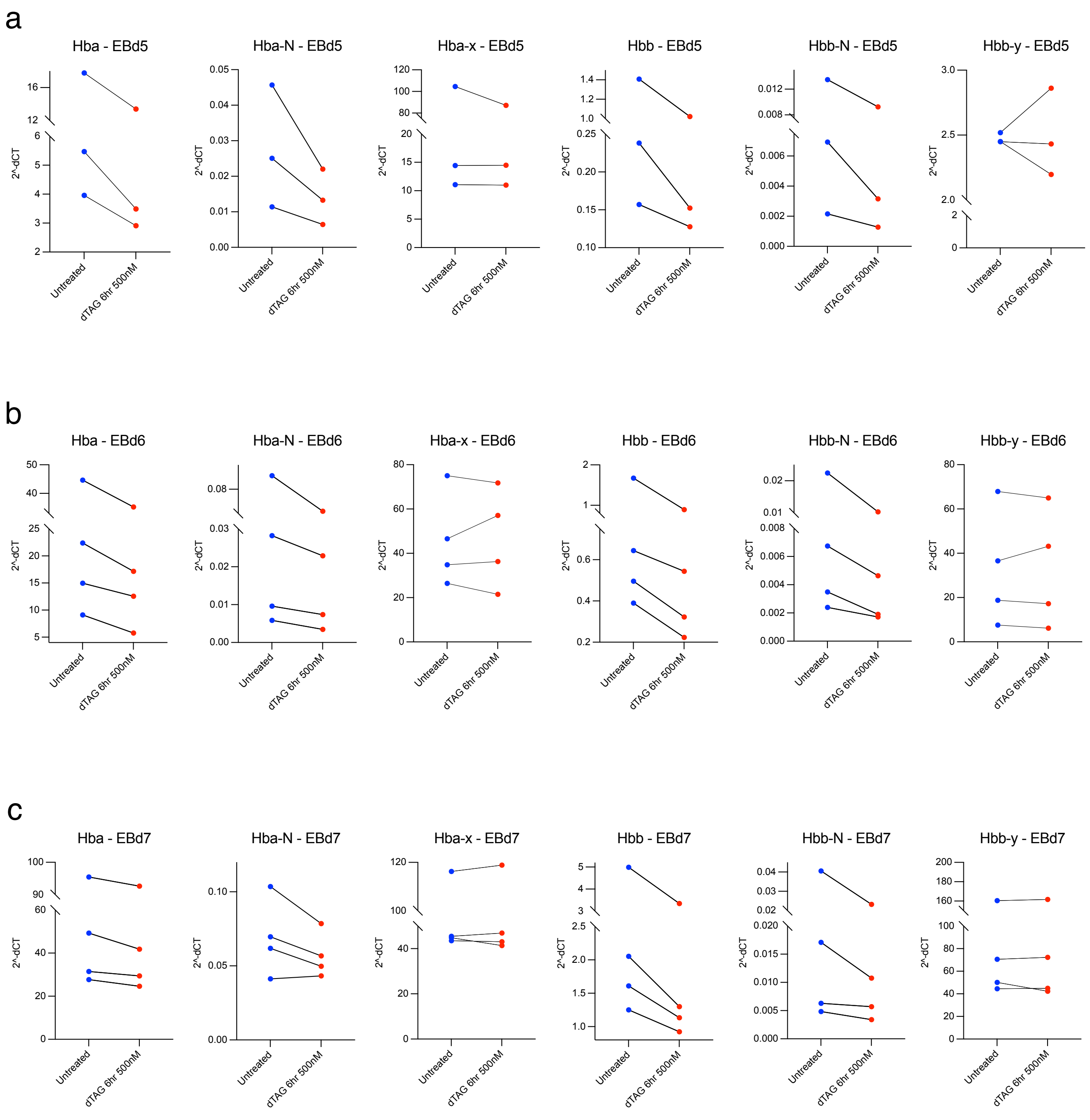

Gene expression by qPCR for adult alpha- and beta-globin genes in their mature and nascent forms respectively (*Hba, HbaN* and *Hbb, HbbN*) and their embryonic forms (*Hba-x, Hbb-y*), by qPCR in **(a)** EB day 5 CD71+ cells (n=3), **(b)** EB day 6 CD71+ cells (n=4), **(c)** EB day 7 CD71+ cells (n=4) with or without dTAG treatment, normalised to the housekeeping *Gapdh* gene and presented in 2^-dCT value. Each dot represents one biological replicate, DMSO (blue) and dTAG (red) samples. Lines indicate paired measurements from the same replicate before and after dTAG treatment.

Supplementary Figure 4: Gene expression in EB-derived CD71+ erythroid cells upon acute depletion of RAD21 measured by digital droplet PCR (ddPCR).

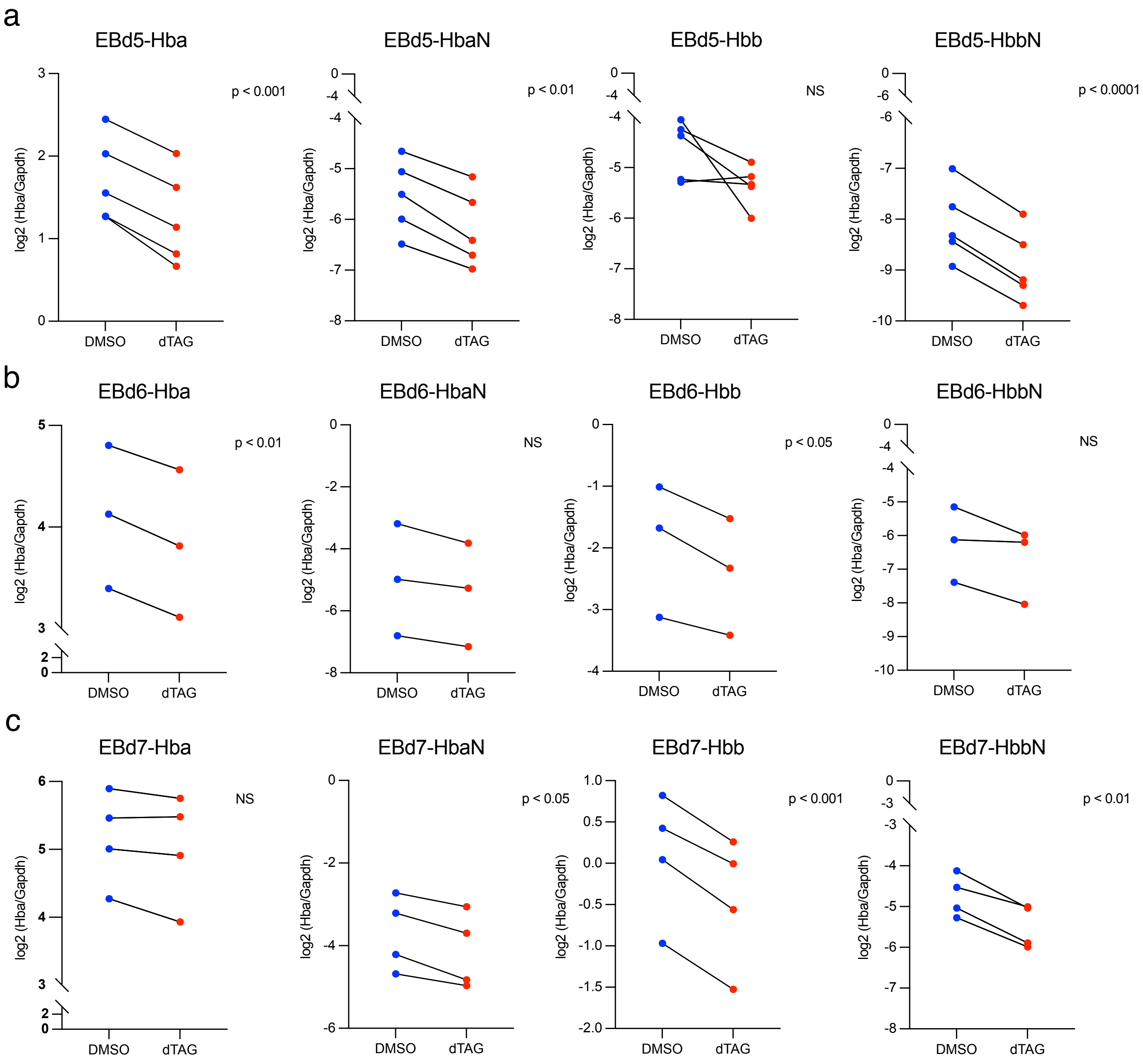

Gene expression for adult alpha- and beta-globin genes in their mature and nascent forms respectively (*Hba, HbaN* and *Hbb, HbbN*) in **(a)** EB day 5 CD71+ cells (n=5), **(b)** EB day 6 CD71+ cells (n=3), **(c)** EB day 7 CD71+ cells (n=4) with or without dTAG treatment were measured by ddPCR, normalised to the housekeeping *Gapdh* gene, and presented in log2 Fold change. Each dot represents one biological replicate, DMSO (blue) and dTAG (red) samples. Lines indicate paired measurements from the same replicate before and after dTAG treatment. P-values were determined by a two-tail paired t-test. Non-significant (NS) $p > 0.05$.

Supplementary Figure 5: Gene expression of non-erythroid genes in EB-derived CD71+ erythroid cells upon acute depletion of RAD21.

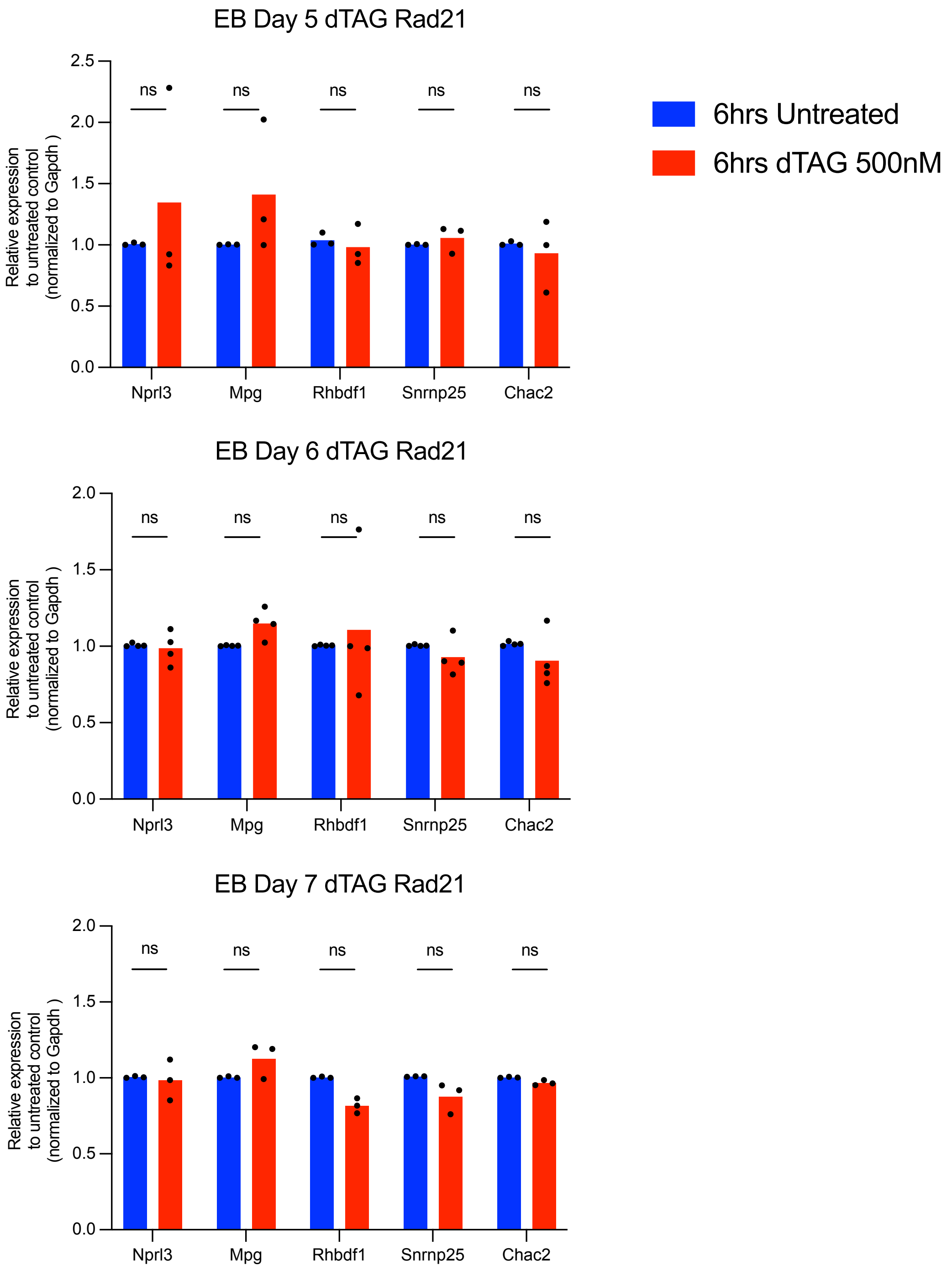

Gene expression by qPCR of the non-erythroid genes surrounding the alpha-globin locus in EB day 5 CD71+ cells (n=3), EB day 6 CD71+ cells (n=4), EB day 7 CD71+ cells (n=3) with or without dTAG, normalised to the housekeeping *Gapdh* gene, and presented as relative fold change to untreated samples. P-values were determined by unpaired two-tailed Student's t-test of the log transformed fold change. Non-significant (NS) $p > 0.05$. Black dots represent single data points.

Supplementary Figure 6: Quantification of the smFISH foci in the EB-derived CD71+ erythroid cells with or without dTAG treatment.

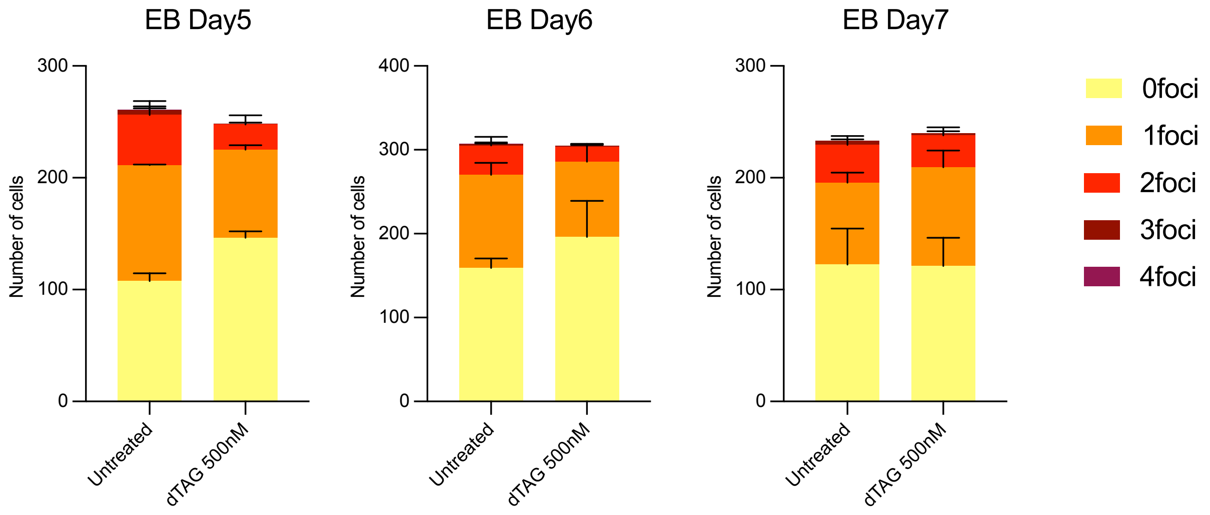

Number of active foci of alpha-globin expression per cell in day 5, day 6 and day 7 EB-derived CD71+ erythroid cells with or without dTAG treatment. Colour of the bar chart represents the number of foci per cell as indicated in the figure. 200-300 cells were analysed in each group. Error bars indicate standard deviation.

Supplementary Figure 7: Capture-C analysis of EB-derived CD71+ erythroid cells with or without dTAG treatment at the early and late differentiation stages.

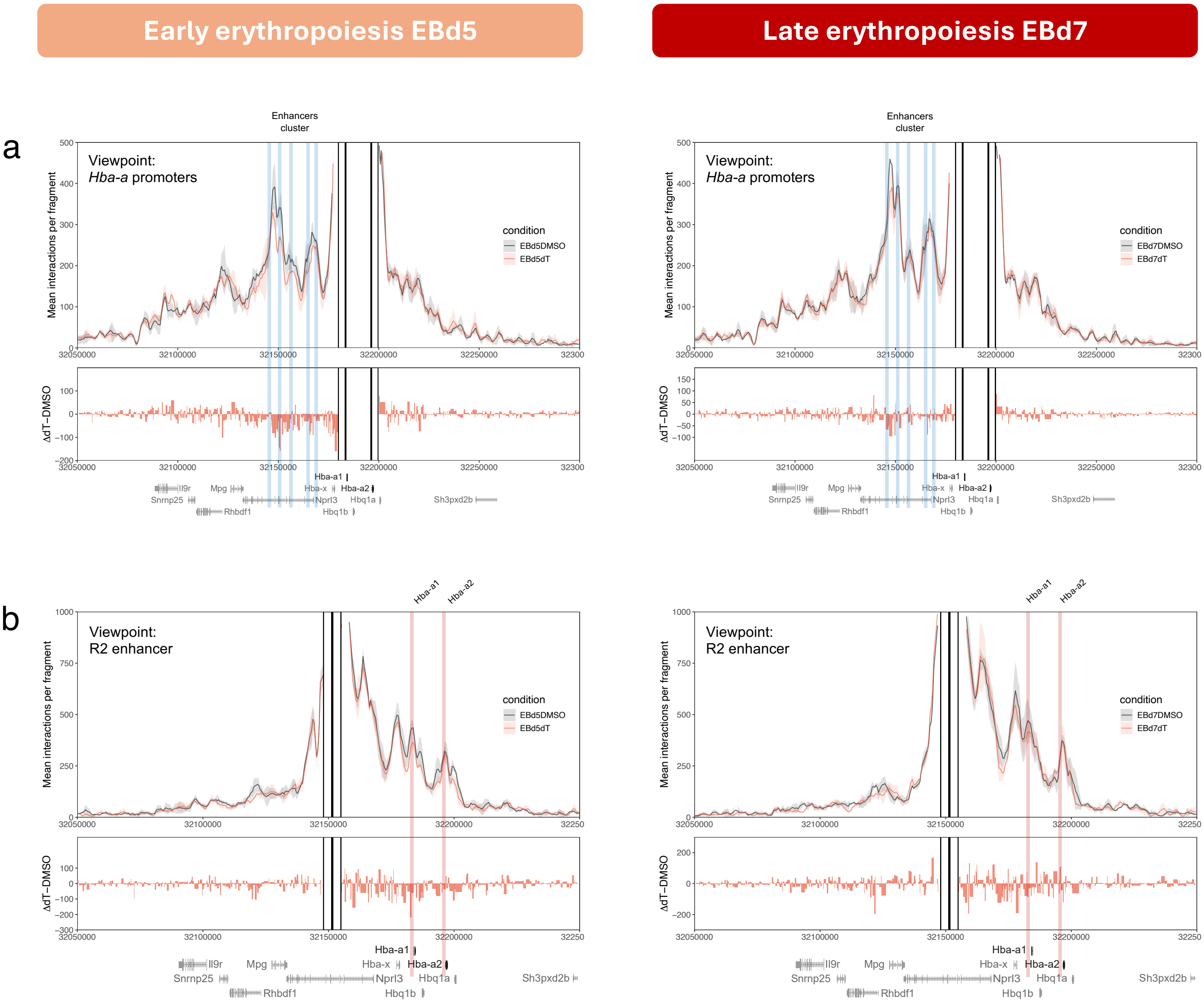

Capture profiles from viewpoints at **(a)** *Hba-a* promoters and **(b)** R2 enhancer. The top profiles show normalised and averaged interactions with a halo representing one standard deviation, for DMSO control treatment (grey, n=3) and dTAG treatment (red, n=3) in the early (EBd5) and late (EBd7) EB-derived erythroid cells. The bottom panels show the subtraction profile calculated as dTAG minus DMSO interaction signal. The positions of the two alpha-globin genes are marked in pink, and five enhancer-like elements in blue. The exclusion zones around capture probes are marked by grey lines and the position of the viewpoint by a black line.

Supplementary Figure 8: Chromatin accessibility profiles at the alpha-globin locus for the different CTCF insertion models.

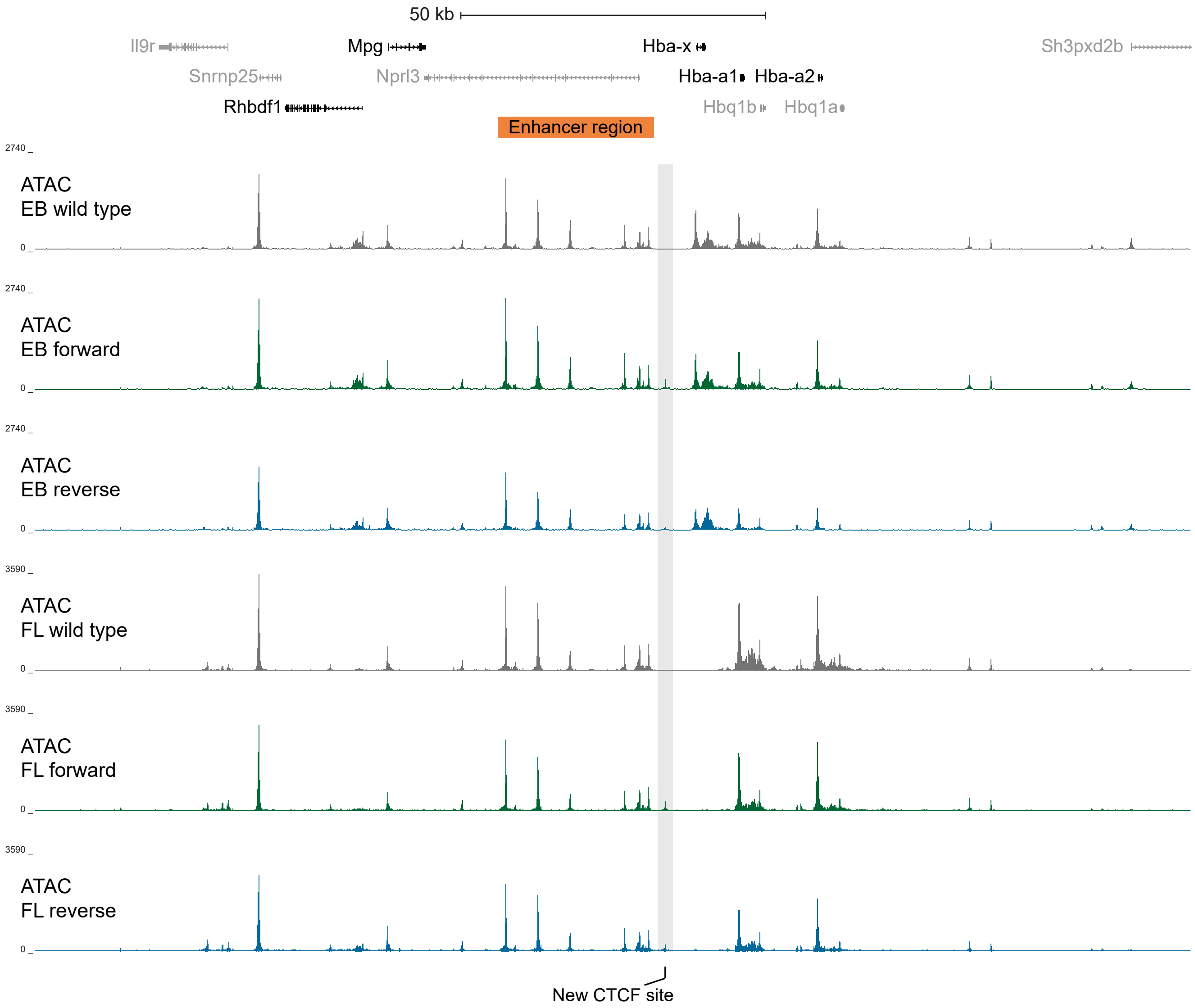

Top three tracks represent ATAC-seq profiles at the native locus (EB wildtype, grey), the locus where CTCF was inserted in the forward (EB Forward, green), and reverse orientations (EB Reverse, blue) in the EB-derived erythroid cells. Bottom three tracks represent the ATAC-seq profiles at the locus in the wildtype (grey), Forward (green), and Reverse (blue) models in the mouse fetal liver-derived erythroid cells. The position of the new inserted CTCF site is highlighted in grey. Orange bar at the top indicates the alpha-globin cluster of 5 enhancer-like elements.

Supplementary Figure 9: RAD21 ChIP-seq at the CTCF insertion site in the EB-derived primitive erythroid cells.

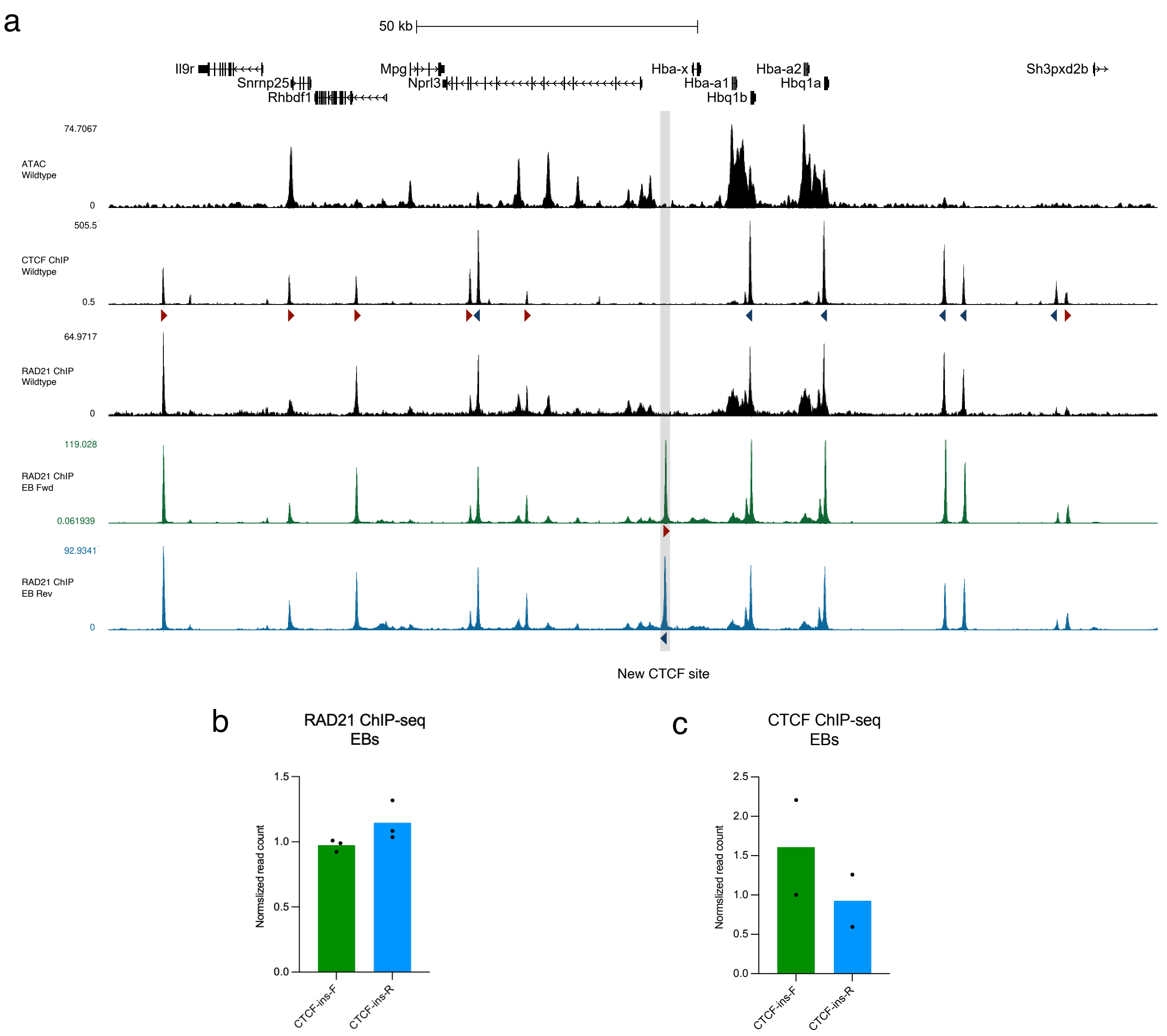

(a) Top three tracks: ATAC-seq, CTCF ChIP-seq, and RAD21 ChIP-seq tracks in the wild-type erythroid cells. Bottom two tracks: RPKM-normalised RAD21 ChIP-seq tracks in Forward (Fwd, green, n=3) and Reverse (Rev, blue, n=3) CTCF insertion models in three different EB clones. CTCF binding site orientation is indicated by forward arrows (red) and reverse arrows (blue). The insertion site is highlighted in grey. **(b)** RAD21 ChIP read counts and **(c)** CTCF ChIP read counts at the CTCF insertion site normalised to the average number of reads at all the other peaks in the genome from ChIP-seq data, in both Forward (CTCF-ins-F) and Reverse (CTCF-ins-R) CTCF insertion models. Black dots represent single datapoints. Due to the small number of biological replicates (n=3), statistical power was limited (Mann-Whitney U-test, two-side p=0.1). Erythroid cells derived from the EB system were previously shown to be predominantly in primitive nature. In which, the adult *Hba-a1* and *Hba-a2* genes; and the embryonic *Hba-x* gene are all actively transcribed[26]. Cohesin recruitment in all the active genes may contribute to increase accumulation cohesin at the CTCF-forward insertion site.

Supplementary Figure 10: RAD21 and STAG2 ChIP-seq at the inserted CTCF binding sites.

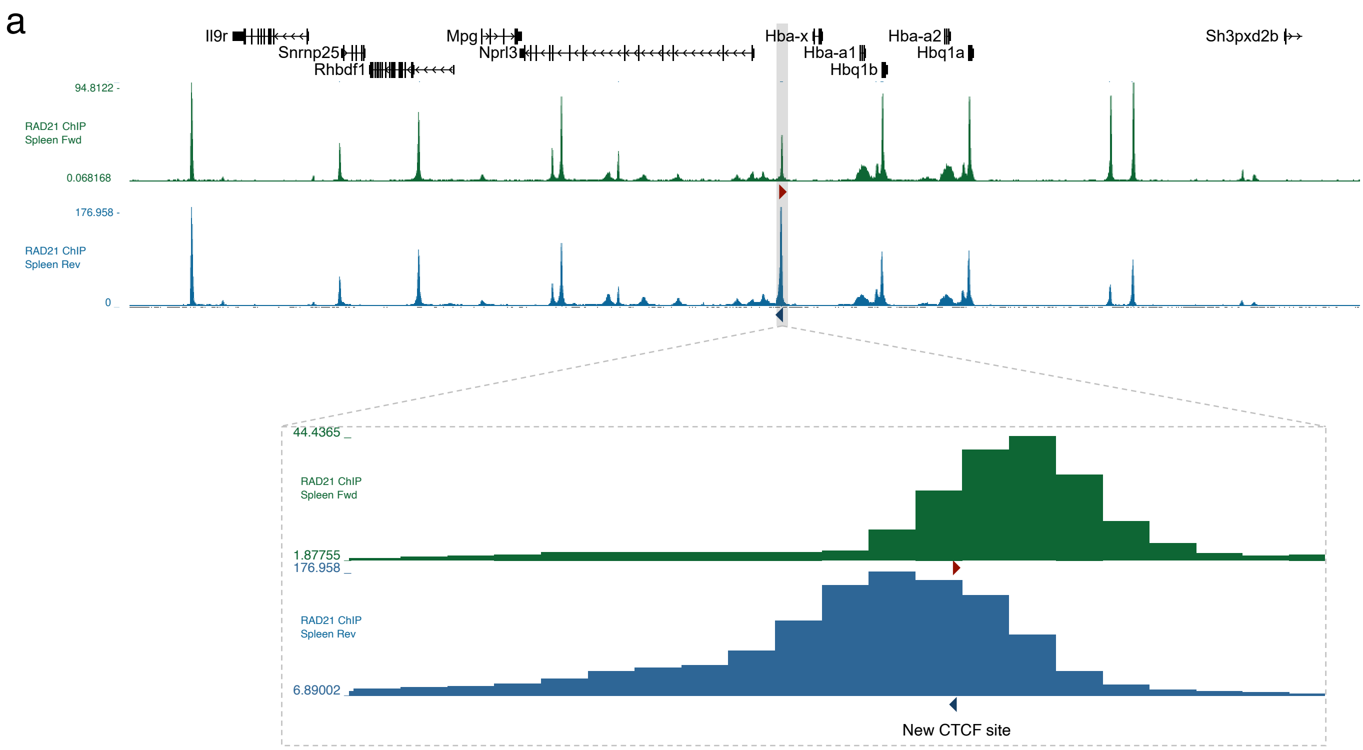

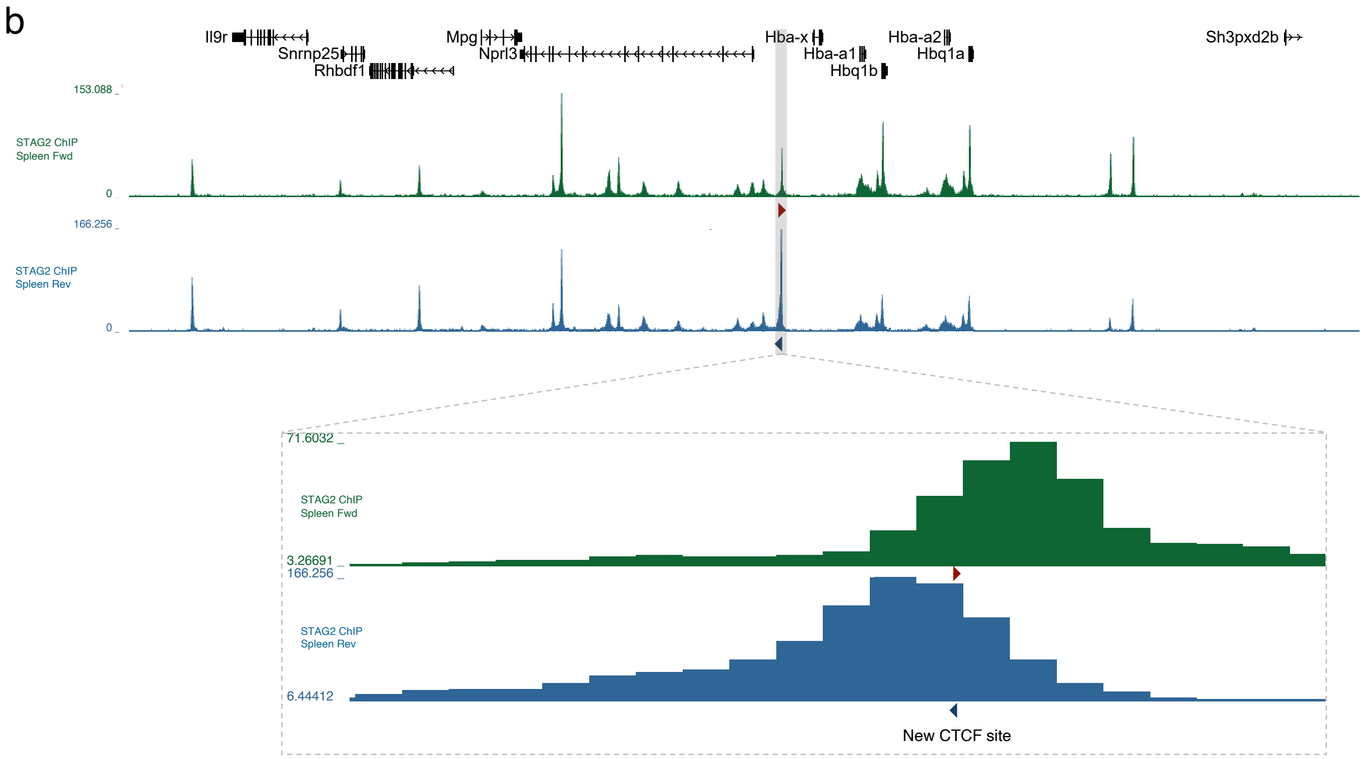

Supplementary Figure 10: RAD21 and STAG2 ChIP-seq at the inserted CTCF binding sites. [continued]

(a) Top, RAD21 ChIP-seq data showing two tracks spanning 180kb around the alpha-globin locus where CTCF was inserted in the forward (green track, red arrow), and reverse orientations (blue track, blue arrow). Below, a zoomed-in view of the RAD21 ChIP-seq peaks at the inserted CTCF binding sites (1kb) in the forward (green track) and reverse insertion models (blue track) from the mouse spleen-derived erythroid cells, **(b)** same as in a, with the STAG2 ChIP-seq data. CTCF insertion and cohesin-binding site is shaded in grey.

Supplementary Figure 11: Zeta-globin gene expression in embryonic and adult erythroid cells in the CTCF insertion models.

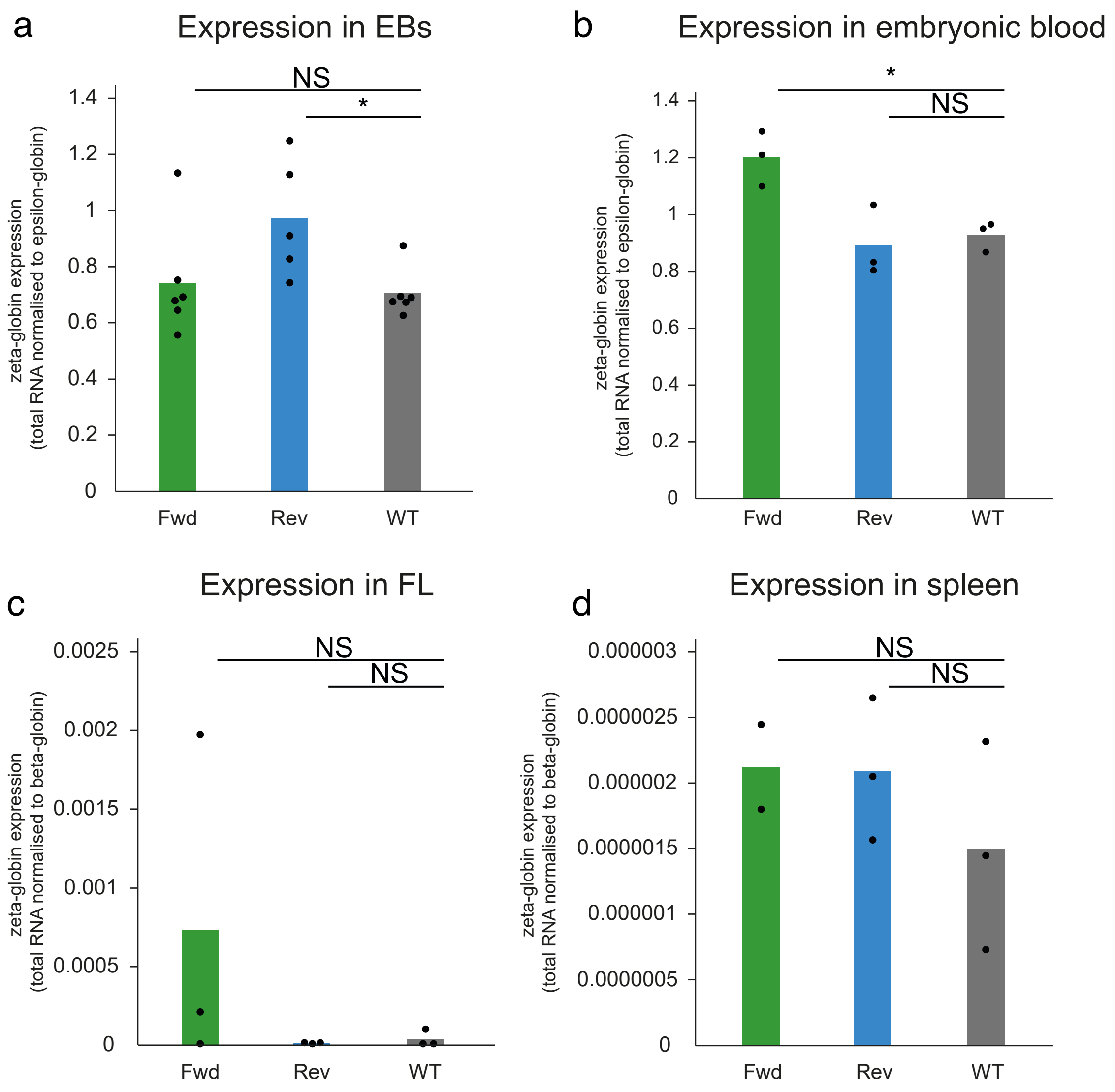

Expression of the embryonic zeta-globin in **(a)** EB-derived erythroid cells, normalised to embryonic epsilon-globin in wildtype (WT, n=6), Reverse (Rev, n=5), and Forward insertion models (Fwd, n=6), **(b)** in E10.5 embryonic blood normalised to epsilon-globin (n=3 for all models), **(c)** in cultured E12.5 fetal liver-derived Ter119+ erythroid cells normalised to adult beta-globin (n=3 for all models), and **(d)** in spleen-derived Ter119+ erythroid cells normalised to beta-globin (n=2 for Fwd, n=3 for Rev and WT). Bar plot shows the mean ratio with individual data points marked by black dots. P-values were obtained using an unpaired two-tailed Student's t-test. Non-significant (NS) $p > 0.05$, and * $p > 0.05$.

Supplementary Figure 12: Gene expression of the alpha-globin neighboring non-erythroid genes in the CTCF insertion models.

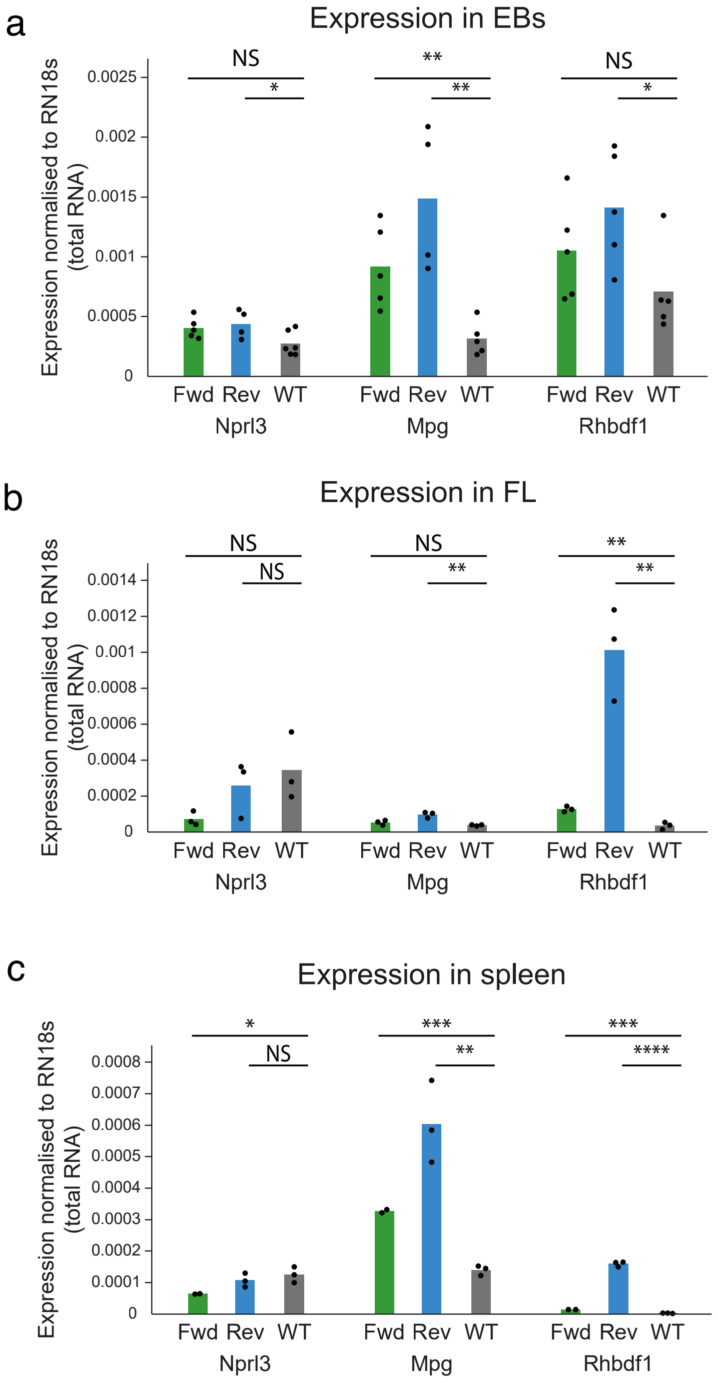

Expression of *Nprl3*, *Mpg*, and *Rhbdf1* genes normalised to *Rn18s* in **(a)** EB-derived erythroid cells in wildtype (WT, n=5), Reverse (Rev, n=4), and Forward insertion models (Fwd, n=5), **(b)** in cultured E12.5 fetal liver-derived Ter119+ erythroid cells (n=3 for all), and **(c)** in spleen-derived Ter119+ erythroid cells in Fwd (n=2), Rev (n=3), and WT (n=3). Bar plots show the mean ratio with individual data points marked by black dots. P-values were obtained using an unpaired two-tailed Student's t-test. Non-significant (NS) $p > 0.05$, * $p < 0.05$, ** $p < 0.01$, *** $p < 0.001$, **** $p < 0.0001$.

Supplementary Figure 13: A schematic summery of the CTCF binding site insertion models and reporter assay at the alpha-globin locus.

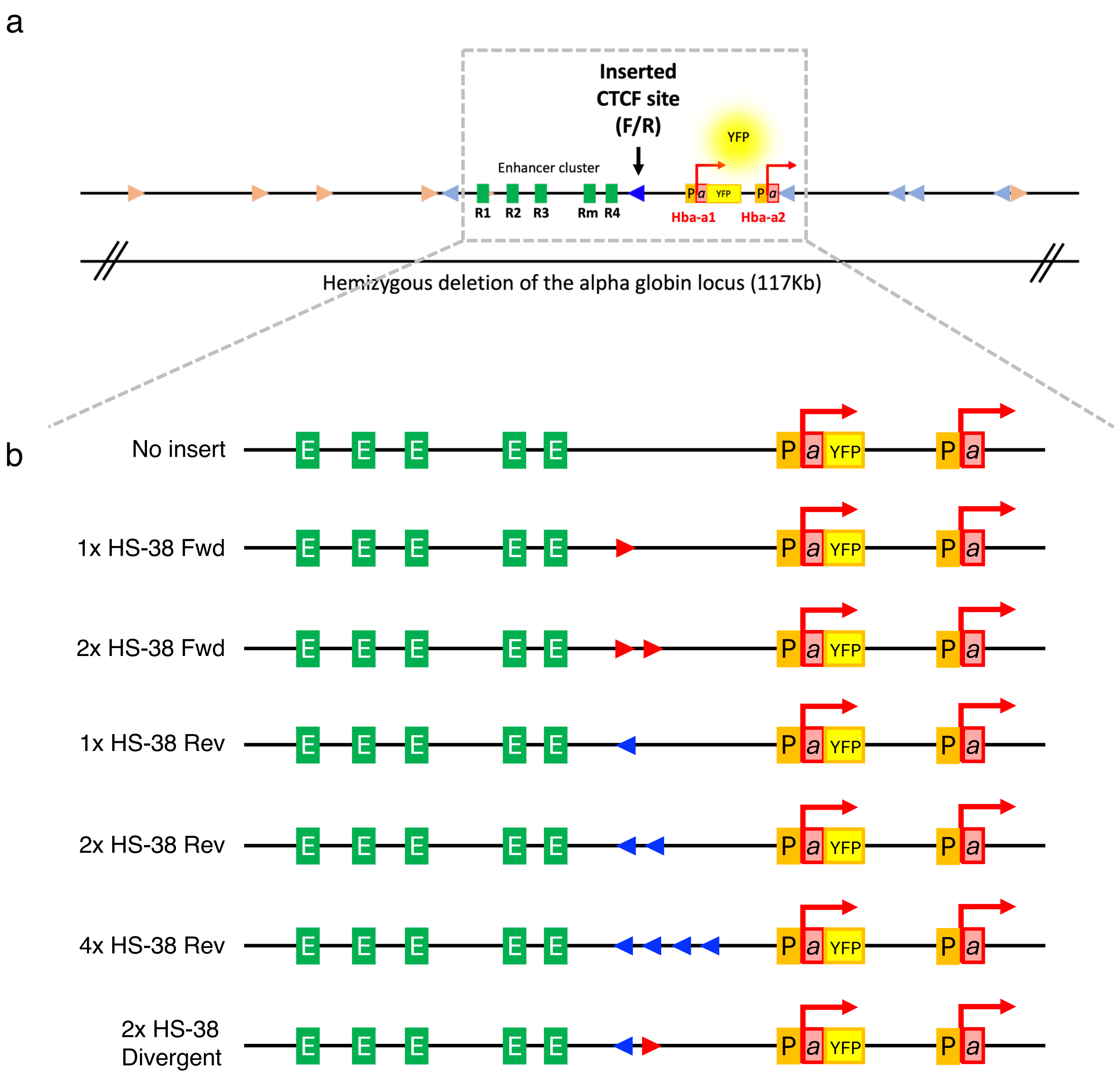

(a) The alpha-globin locus is depicted with the two alpha-globin adult genes *Hba-a1* (YFP-tagged) and *Hba-a2*, the cluster of 5 enhancer-like elements (green boxes), CTCF sites flanking the locus (light orange and blue arrows) and the inserted CTCF site (dark blue arrow). **(b)** Schematic representation of the locus representing models with no insert, single insert in forward (red arrow) and reverse (blue arrow), or variable copies and orientations of HS-38 CTCF binding sites, spaced by 500bp.

Supplementary Figure 14: CTCF and RAD21 ChIP-seq at the multiple CTCF insertion model.

a

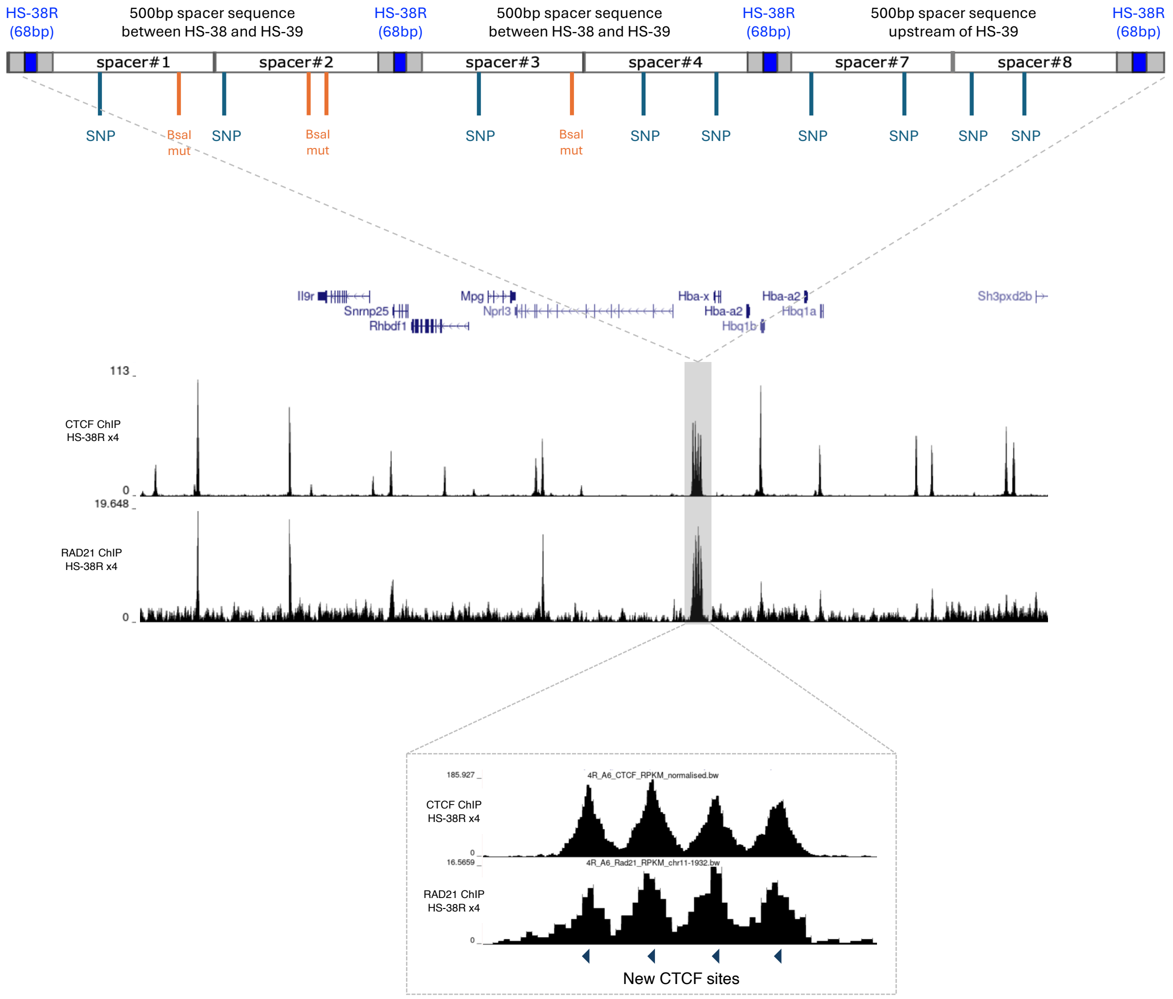

(a) Schematic representation of the engineered four CTCF site (4x HS-38) insertion cassette. The construct contains four copies of the 68bp HS-38R CTCF-binding sequence arranged in tandem, separated by 500 bp spacer fragments derived from the endogenous alpha-globin locus (between HS-38 and HS-39, or upstream of HS-39) to provide sufficient distance between adjacent motifs and avoid interference with CTCF binding. Spacer fragments also contain nine SPRET-specific SNPs and four BsaI-disrupting point mutations, enabling unambiguous mapping of sequencing reads to the inserted cassette rather than the endogenous locus and preventing unwanted cleavage during the cloning process. **(b)** Top, CTCF and RAD21 ChIP-seq tracks showing the region spanning 200kb around the alpha-globin locus where four CTCF binding sites HS-38 were inserted in the reverse orientation (CTCF insertion site is shaded in grey). Below, a zoomed-in view of the CTCF and RAD21 ChIP-seq peaks at the insertion site with four CTCF binding sites in the reverse orientation (blue arrows).

Supplementary Figure 15: RAD21 enrichment levels at all CTCF sites across the alpha-globin locus in the CTCF Forward and Reverse insertion models.

a

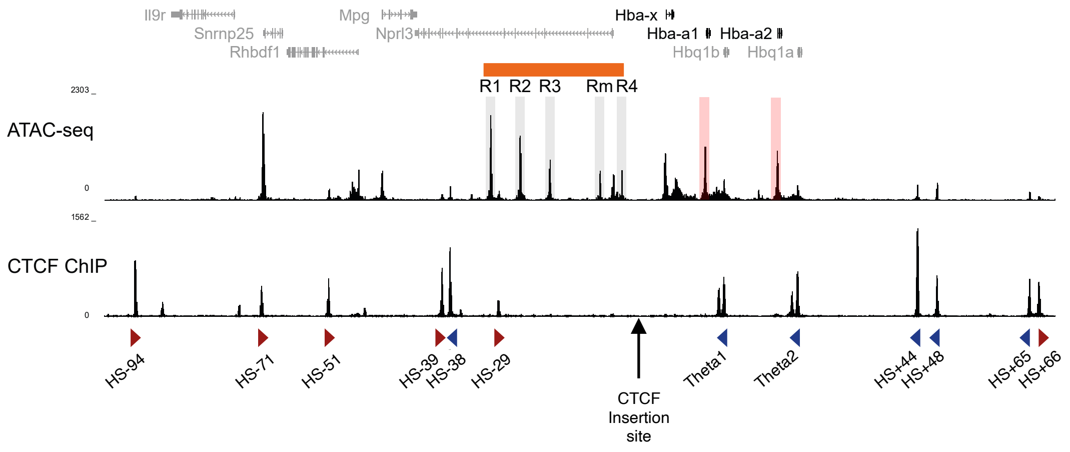

b

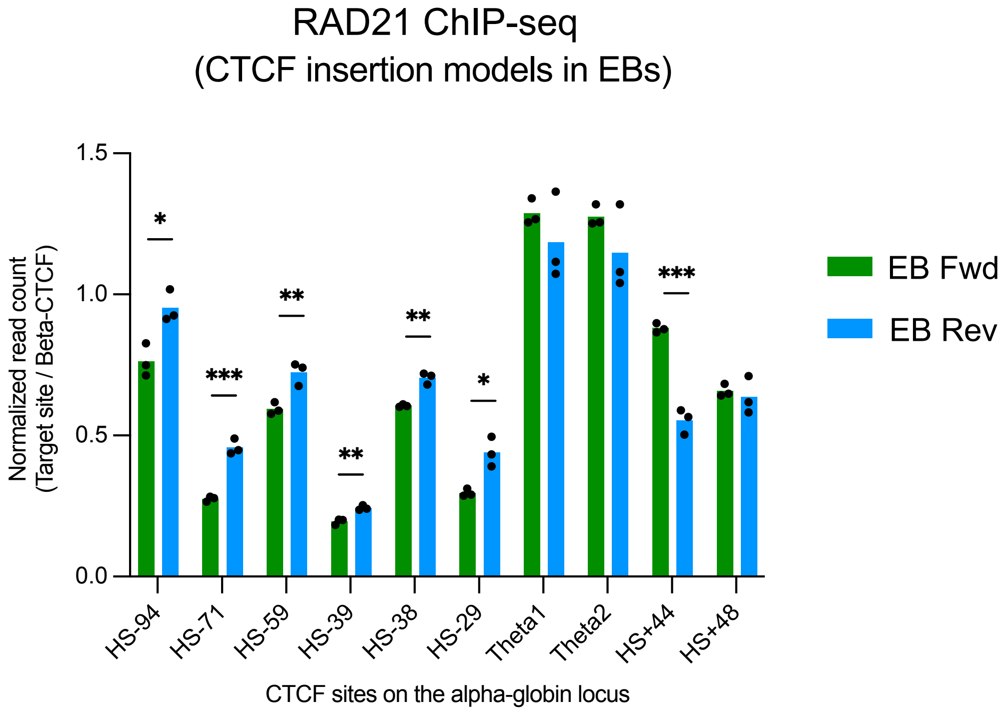

(a) ATAC-seq and CTCF ChIP-seq tracks indicate the position of the enhancers (grey bars), promoters (pink bar), and annotated CTCF sites (red and blue arrows for forward and reverse orientations respectively) at the alpha-globin locus. (blue). **(b)** Bar plots of the CTCF forward and reverse insertion model (Fwd, green, Rev, blue) show the mean RAD21 normalized read count with individual datapoints marked by black dots (n=3). P-values were obtained using an unpaired two-tailed Student's t-test. Non-significant (NS) p > 0.05, * p <0.05, ** p < 0.01, *** p <0.001, **** p <0.0001.

Supplementary Table 1: sgRNA and primer sequences used in this study

Name	Sequence (5' to 3')
pDN1 (CTCF insertion sgRNA target 1)	GCTGTAGTGTAACTAACTGC
p1 (CTCF insertion sgRNA target 2)	GCTTCAAGAACTGCCTTCCTG
Endogenous *Rad21* tagging (sg2)	CCTCAGATAATATGGAACCGTGG
Left homology arm forward	TCCTTGGCTATATATTGCACTTGA
Left homology arm reverse	TGCGAGAGCTCTACACCTTCATTCAGGGACATTGC
Right homology arm forward	TGTAGAGCTCTCGCAACAATTAAAAGAGAATAACCGCTGT
Right homology arm reverse	ATCCCTTGGAGTTCAGAGTTACAG
Screen 1 (CTCF side insert) forward	GAGTGGCTCTGAGGGTTGAG
Screen 1 (CTCF side insert) reverse	GATCCTGTCGTCCTGGGTTA
Screen 2 (Neo side insert) forward	TCGCGAGTCGAAGTTCCTAT
Screen 2 (Neo side insert) reverse	CCAGAGGATCTGGGTTCAAA
Screen 3 (across insert) forward	AGTTTGTGGTTGGGAGAGGAGG
Screen 3 (across insert) reverse	GTGCACACGGGGAAGAAGTTTG
qPCR expression *Hba-a1/2* forward	CTGGGGAAGACAAAAGCAAC
qPCR expression *Hba-a1/2* reverse	GCCGTGGCTTACATCAAAGT
qPCR expression *Hbb-b* forward	ATGGCCTGAATCACTTGGAC
qPCR expression *Hbb-b* reverse	ACGATCATATTGCCCAGGAG
qPCR expression *Nprl3* forward	GCTCTTCAGGTACCCCTTCC
qPCR expression *Nprl3* reverse	ATGTTCGCCAGTGTTGTTGA
qPCR expression *Mpg* forward	CTTCTCCAGCCCAGAGGAC
qPCR expression *Mpg* reverse	ATGCCTCAGTCTCCACAATG
qPCR expression *Rn18s* forward	GTAACCCGTTGAACCCCATT
qPCR expression *Rn18s* reverse	CCATCCAATCGGTAGTAGCG
qPCR expression *Rhbdf1* forward	ATCCTAGTGCCCCAGACCTT
qPCR expression *Rhbdf1* reverse	CGGCCACTTGGTGATATCTT
qPCR expression *Hba-x* forward	ATGCGGTTAAGAGCATCGAC
qPCR expression *Hba-x* reverse	GGGACAGGAGCTTGAAGTTG
qPCR expression *Hba-aN* forward	GTGTGGATCCCGTCAACTTC
qPCR expression *Hba-aN* reverse	CCACTATGTTCCCTGCCTTG
qPCR expression *Hbb-bN* forward	ATGGCCTGAATCACTTGGAC
qPCR expression *Hbb-bN* reverse	AGAATAGCCAGGGGAAGGAA
qPCR expression *Hbb-y* forward	TGGCCTGTGGAGTAAGGTCAA
qPCR expression *Hbb-y* reverse	GAAGCAGAGGACAAGTTCCCA
qPCR expression *Gapdh* forward	ACGGCAAATTCAACGGCA
qPCR expression *Gapdh* reverse	AGATGGTGATGGGCTTCCC

Supplementary Table 2: Donor sequences for CRISPR-cas9 editing

Name	Sequence (5’ to 3’)
CTCF insertion fragment containing CTCF binding site	CGCACGACGAGCTCGTGGCTAACTTTAAATAATGCCAATTATTTAAAGTTAAAACGCA<br>TAGCTCGCTCAATGGAAGTTCCTATTCCGAAGTTCCTATTCTCTAGAAAGTATAGGAA<br>CTTCGACTCGGTACCGAGCCGAATTCTTTCAGAAGTTCCTATTCCGAAGTTCCTATTC<br>TCTAGAAAGTATAGGAACTTCACTTCTCAATATGTGAGCACTATAACTTCGTATAATGT<br>ATGCTATACGAAGTTATGGTCATTGGCTCACGGAACTTATAACTTCGTATAAAGTATC<br>CTATACGAAGTTATGCAAACTGAGGTCCTGGGTAGGCCTCTGCTACCCTCTGGTGGC<br>CTCAGGGTGCATAGCACTACCAGGTGAGGATACTGGCATTATAACTTCGTATAGCAT<br>ACATTATACGAAGTTATGAGATCCTGTCGTCCTGGGTTATAACTTCGTATAGGATACT<br>TTATACGAAGTTATTATGAATTTCGTATTTGCAGCTAACTTTAAATAATGCCAATTATTT<br>AAAGTTACTGCAGAGCTCTCGGTCCTGG
Donor Sequence (including homology arms) (5'-3') RAD21-FKBPF36V-3xFLAG	GGGTTGGAAGGTTATCAGGGGCCGGTTTGATTTTGGTTTTGTTTTCACTTTAAAATCT<br>GCTGAGTGTTTGTTTTTGCTAACTCACATCTCTGTTGTGGCGCCTTCACTTACTTCTT<br>GAACTCTGTATGCCTTAAGATAATATGCTTTAGATAATAAGGCCTTTACCTTAGTGACT<br>AGCATGGGAACCACTTGCGTAACTACACGAATGTCCAGTCCCAGCTCCAGCCAAAC<br>GGAGCTCACTTTGACCAGTGCCAAAATTGCATCTTCTGGTTACTACTTTGTGCGTGAG<br>TTACTTGAAATCATCTGCTTTGTTTTGTTCTATTTCAGCGAGCTCTTGCTAAAACTGGA<br>GCAGAGTCTATCAGTTTGCTTGAGCTGTGTCGAAACACAAACCGAAAGCAGGCAGCA<br>GCAAAGTTCTACAGCTTTTTGGTTCTTAAGAAGCAGCAAGCCATCGAGCTCACACAG<br>GAAGAGCCGTACAGTGACATCATTGCAACGCCCGGCCCACGCTTTCACATCATCACC<br>GGTGGAGTGCAGGTGGAAACCATCTCCCCAGGAGACGGGCGCACCTTCCCCAAGC<br>GCGGCCAGACCTGCGTGGTGCACTACACCGGGATGCTTGAAGATGGAAAGAAAGTT<br>GATTCCTCCCGCGACAGAAACAAGCCCTTTAAGTTTATGCTAGGCAAGCAGGAGGTG<br>ATCCGAGGCTGGGAAGAAGGGGTTGCCCAGATGAGTGTGGGTCAGAGAGCCAAAC<br>TGACTATATCTCCAGATTATGCCTATGGTGCCACTGGGCACCCAGGCATCATCCCAC<br>CACATGCCACTCTCGTCTTCGATGTGGAGCTTCTAAAACTGGAACAATTGGGCGGAT<br>CCGGGAGCGCCTGGAGCCACCCCCAGTTCGAGAAGGGCGGCGGCAGCGGCGGCG<br>GCAGCGGCGGCAGCGCCTGGAGCCACCCCCAGTTCGAGAAGGTCGACAGATCTTG<br>ATTGATTAAAGATCTTCTAGAGGAGCTAGATGTGTTCGAGCTAGTGATAACTCACTAG<br>TACATACAAATTGCCCCCGTGTGCAGGGCACCAAAACCCTTTAAGAAAGTTTTTAGAT<br>TTCTGTTTGTACAAAAATCTTTGCCTTTTCTTTCTTCTTTTTCCCCCCAGTGTTTCTAAT<br>TTTGTCAACCATATTTTTAAGGGAAACTGCTTATTTGGGTTGGGTTTGTATTCCTGGA<br>GAAAACAGTAGCCCAAGAACCCAGAAGACTTTTAACAGTTCA

Supplementary Table 3: Donor sequences for CTCF reporter assay

Name	Sequence (5’ to 3’)
neutral control sequence	AATGTAATGAAAATATGGCTAATTAATCTTTACATTCTGTCTTGGCCAAGATGCAGAGAAGCGACAGT
mouse alpha locus HS-94 CTCF site (forward)	CCCAGGAGGCTGCATTACCACAGATGGACAGTAGAGGGAGACAGAGATCGTGAGTTGAAAATTAAAAG
mouse alpha locus HS-94 CTCF site (reverse)	CTTTTAATTTTCAACTCACGATCTCTGTCTCCCTCTACTGTCCATCTGTGGTAATGCAGCCTCCTGGG
mouse alpha locus HS-71 CTCF site (forward)	CACGCGAGTGGCAACGGCAGGCCCCAGGCAGCAGGAGGCGCCCGCCTCCCAGCGTCACCTTGTTGGGT
mouse alpha locus HS-71 CTCF site (reverse)	ACCCAACAAGGTGACGCTGGGAGGCGGGCGCCTCCTGCTGCCTGGGGCCTGCCGTTGCCACTCGCGTG
mouse alpha locus HS-59 CTCF site (forward)	CCTTGGAGCAGAAGCCACAATAAATGACCACGAGGTGGCGCCAACTGCCAAAAAAGGCATCGAGGCAG
mouse alpha locus HS-59 CTCF site (reverse)	CTGCCTCGATGCCTTTTTTGGCAGTTGGCGCCACCTCGTGGTCATTTATTGTGGCTTCTGCTCCAAGG
mouse alpha locus HS-39 CTCF site (forward)	TCATGGATTCAAAGCCACTGAGGCCTGGCCACTGGGGGCGCCATTCGCCATTAAAAGGTCCTGCTGGG
mouse alpha locus HS-39 CTCF site (reverse)	CCCAGCAGGACCTTTTAATGGCGAATGGCGCCCCCAGTGGCCAGGCCTCAGTGGCTTTGAATCCATGA
mouse alpha locus HS-38 CTCF site (forward)	CACCTGGTAGTGCTATGCACCCTGAGGCCACCAGAGGGTAGCAGAGGCCTACCCAGGACCTCAGTTTG
mouse alpha locus HS-38 CTCF site (reverse)	CAAACTGAGGTCCTGGGTAGGCCTCTGCTACCCTCTGGTGGCCTCAGGGTGCATAGCACTACCAGGTG
mouse alpha locus HS-29 CTCF site (forward)	CAAATTCCTGTGTCCCTCCAAATTGGTCCACTGGGTGGCACTTGGAGGCCTCAAGCCCTGAGCTGTGC
mouse alpha locus HS-29 CTCF site (reverse)	GCACAGCTCAGGGCTTGAGGCCTCCAAGTGCCACCCAGTGGACCAATTTGGAGGGACACAGGAATTTG
mouse alpha locus Theta1 CTCF site (forward)	CTCAAAGACGTCCTGAAACACAAGAGGCCGCCAGGGGGCGCTGCATCGTTCCAGGATGCCTAGGTGTT
mouse alpha locus Theta1 CTCF site (reverse)	AACACCTAGGCATCCTGGAACGATGCAGCGCCCCCTGGCGGCCTCTTGTGTTTCAGGACGTCTTTGAG
mouse alpha locus Theta2 CTCF site (forward)	CTCAAAGATGTCCTGAAACACAAGAGGCCGCCAGGGGGCGCTGCATCGTTCCAGGATGCCTAGGTGTT
mouse alpha locus Theta2 CTCF site (reverse)	AACACCTAGGCATCCTGGAACGATGCAGCGCCCCCTGGCGGCCTCTTGTGTTTCAGGACATCTTTGAG
mouse alpha locus HS+44 CTCF site (forward)	GAAAAGCCTTGCACCTACTTATAGTGGCCTGCAGGGGGCGCCCCAGAGACCTCCTGCCCGGTAGTCAT
mouse alpha locus HS+44 CTCF site (reverse)	ATGACTACCGGGCAGGAGGTCTCTGGGGCGCCCCCTGCAGGCCACTATAAGTAGGTGCAAGGCTTTTC

Supplementary Table 3: Donor sequences for CTCF reporter assay [continued]

Name	Sequence (5' to 3')
mouse alpha locus HS+48 CTCF site (forward)	CTTGGAGCTGCGAAGTTCCGAGTCCCGCCACACGGGGGTGCTCGTCGCCTTCTTATATCTCCGGTAGC
mouse alpha locus HS+48 CTCF site (reverse)	GCTACCGGAGATATAAGAAGGCGACGAGCACCCCCGTGTGGCGGGACTCGGAACTTCGCAGCTCCAAG
mouse alpha locus HS+65 CTCF site (forward)	CCAGGTTGGAGCACTACATCAATCTCTCCTGCAGGTGGCGCCCTTCCGCGCGACTCACTCTTATGGAC
mouse alpha locus HS+65 CTCF site (reverse)	GTCCATAAGAGTGAGTCGCGCGGAAGGGCGCCACCTGCAGGAGAGATTGATGTAGTGCTCCAACCTGG
mouse alpha locus HS+66 CTCF site (forward)	GTGTGTGAATGCAGGTGCCTACAGTGGACAGAAGAGGGCGCCAGATCCTCTGGAGCTGGAGTTTCAGG
mouse alpha locus HS+66 CTCF site (reverse)	CCTGAAACTCCAGCTCCAGAGGATCTGGCGCCCTCTTCTGTCCACTGTAGGCACCTGCATTCACACAC
mouse beta locus 3'HS1 CTCF site (forward)	AGAGCTCTGAGGCATGTTCTCAGTCAACCTCAAGGGGGCAGTATTGAGCTTGGAATTCAGTATCAACT
mouse beta locus 3'HS1 CTCF site (reverse)	AGTTGATACTGAATTCCAAGCTCAATACTGCCCCCTTGAGGTTGACTGAGAACATGCCTCAGAGCTCT
mouse HoxA5I6 CTCF site (forward)	TCGGAAGCGAAGCGATGCGCCCAGTCTCCAGCGGGTGGCGCTCGAGTCCGACTGAACGGCGGCAACGG
mouse HoxA5I6 CTCF site (reverse)	CCGTTGCCGCCGTTCAGTCGGACTCGAGCGCCACCCGCTGGAGACTGGGCGCATCGCTTCGCTTCCGA
mouse H19-Igf2 m3 CTCF site (forward)	TTATGTGCAACAAGGGAACGGATGCTACCGCGCGGTGGCAGCATACTCCTATATATCGTGGCCCAAAT
mouse H19-Igf2 m3 CTCF site (reverse)	ATTTGGGCCACGATATATAGGAGTATGCTGCCACCGCGCGGTAGCATCCGTTCCCTTGTTGCACATAA
mouse Sox9 CBS1 CTCF site (forward)	GAAAATATTGTGCAAGGCAACATGTTACCAGCAGGTGGCAGTCCAGTAGACTTCTACAGAACTGTGGC
mouse Sox9 CBS1 CTCF site (reverse)	GCCACAGTTCTGTAGAAGTCTACTGGACTGCCACCTGCTGGTAACATGTTGCCTTGCACAATATTTTC
mouse Sox9 CBS2 CTCF site (forward)	AAGAAAAGGTGTACTCTCCGTTGTCTACCGCCAGATGGCAGCATGCACACACCAATTAAAATTTGTTC
mouse Sox9 CBS2 CTCF site (reverse)	GAACAAATTTTAATTGGTGTGTGCATGCTGCCATCTGGCGGTAGACAACGGAGAGTACACCTTTTCTT
mouse Epha4-Pax3 R4 CTCF site (forward)	TACGACAGAGGCTGCGTCTCACTATGTCCATGCGGGGGCGCTCTTTTGCAGTCTCTCTGCAGACCTCA
mouse Epha4-Pax3 R4 CTCF site (reverse)	TGAGGTCTGCAGAGAGACTGCAAAAGAGCGCCCCCGCATGGACATAGTGAGACGCAGCCTCTGTCGTA

Supplementary Table 3: Donor sequences for CTCF reporter assay [continued]

Name	Sequence (5' to 3')
CTCF reporter assay insertion fragment – HS-38-Fx2	CACCTGGTAGTGCTATGCACCCTGAGGCCACCAGAGGGTAGCAGAGGCCTA<br>CCCAGGACCTCAGTTTGCTTTTCTAGCTCCAGATTCCAGATTTTTGGCAGCCA<br>CTGGTACTTACAGACACACACTGGCATTGGGAGACAGGACATAGACGTTGTT<br>CTCACACAGTGGGTAGATGATGACAGCCTTGCCCCAGTATACCAGGTGAGCT<br>GCCAACTGGAAGACCTGCAGACCAAGGAGAGATCGAAGGACTAAACTTGGA<br>GTCCACGGTTTTGCTAGGCAGTAGGCACATGAACACAGGGACACTGGAGTG<br>CTTGGAGCATGGGCTGGACGACTCCAGCAGAAGCACAGCATTCTCTTGTCCA<br>CAGACACCAGCACCATACTCCAAAGTCATAAAATATGGAGTCTCATCCTACCT<br>GACCCTGCATACTCAAAGGCCCCTATGCACCCTGCTTTCCCCAGAGTCCATC<br>CTCCCATTCTGTTCTCCTGAGGACTCAGATACCAGGAAATACACAAGATACCA<br>TAGCAAGTTCCAGGACAACCAGGGCTACAGAGCAATCCTGTCTCAAACCACC<br>TGGTAGTGCTATGCACCCTGAGGCCACCAGAGGGTAGCAGAGGCCTACCCA<br>GGACCTCAGTTTG
CTCF reporter assay insertion fragment – HS-38-Rx2	CAAACTGAGGTCCTGGGTAGGCCTCTGCTACCCTCTGGTGGCCTCAGGGTG<br>CATAGCACTACCAGGTGCTTTTCTAGCTCCAGATTCCAGATTTTTGGCAGCCA<br>CTGGTACTTACAGACACACACTGGCATTGGGAGACAGGACATAGACGTTGTT<br>CTCACACAGTGGGTAGATGATGACAGCCTTGCCCCAGTATACCAGGTGAGCT<br>GCCAACTGGAAGACCTGCAGACCAAGGAGAGATCGAAGGACTAAACTTGGA<br>GTCCACGGTTTTGCTAGGCAGTAGGCACATGAACACAGGGACACTGGAGTG<br>CTTGGAGCATGGGCTGGACGACTCCAGCAGAAGCACAGCATTCTCTTGTCCA<br>CAGACACCAGCACCATACTCCAAAGTCATAAAATATGGAGTCTCATCCTACCT<br>GACCCTGCATACTCAAAGGCCCCTATGCACCCTGCTTTCCCCAGAGTCCATC<br>CTCCCATTCTGTTCTCCTGAGGACTCAGATACCAGGAAATACACAAGATACCA<br>TAGCAAGTTCCAGGACAACCAGGGCTACAGAGCAATCCTGTCTCAAACCAAA<br>CTGAGGTCCTGGGTAGGCCTCTGCTACCCTCTGGTGGCCTCAGGGTGCATA<br>GCACTACCAGGTG
CTCF reporter assay insertion fragment – HS-38-x2-divergent	CAAACTGAGGTCCTGGGTAGGCCTCTGCTACCCTCTGGTGGCCTCAGGGTG<br>CATAGCACTACCAGGTGCTTTTCTAGCTCCAGATTCCAGATTTTTGGCAGCCA<br>CTGGTACTTACAGACACACACTGGCATTGGGAGACAGGACATAGACGTTGTT<br>CTCACACAGTGGGTAGATGATGACAGCCTTGCCCCAGTATACCAGGTGAGCT<br>GCCAACTGGAAGACCTGCAGACCAAGGAGAGATCGAAGGACTAAACTTGGA<br>GTCCACGGTTTTGCTAGGCAGTAGGCACATGAACACAGGGACACTGGAGTG<br>CTTGGAGCATGGGCTGGACGACTCCAGCAGAAGCACAGCATTCTCTTGTCCA<br>CAGACACCAGCACCATACTCCAAAGTCATAAAATATGGAGTCTCATCCTACCT<br>GACCCTGCATACTCAAAGGCCCCTATGCACCCTGCTTTCCCCAGAGTCCATC<br>CTCCCATTCTGTTCTCCTGAGGACTCAGATACCAGGAAATACACAAGATACCA<br>TAGCAAGTTCCAGGACAACCAGGGCTACAGAGCAATCCTGTCTCAAACCACC<br>TGGTAGTGCTATGCACCCTGAGGCCACCAGAGGGTAGCAGAGGCCTACCCA<br>GGACCTCAGTTTG

Supplementary Table 3: Donor sequences for CTCF reporter assay [continued]

Name	Sequence (5’ to 3’)
CTCF reporter assay insertion fragment – HS-38-Rx4	CAAACTGAGGTCCTGGGTAGGCCTCTGCTACCCTCTGGTGGCCTCAGGGTG<br>CATAGCACTACCAGGTGCTTTTCTAGCTCCAGATTCCAGATTTTTGGCAGCCA<br>CTGGTACTTACAGACACACACTGGCATTGGGAGACAGGACATAGACGTTGTT<br>CTCACACAGTGGGTAGATGATGACAGCCTTGCCCCAGTATACCAGGTGAGCT<br>GCCAACTGGAAGACCTGCAGACCAAGGAGAGATCGAAGGACTAAACTTGGA<br>GTCCACGGTTTTGCTAGGCAGTAGGCACATGAACACAGGGACACTGGAGTG<br>CTTGGAGCATGGGCTGGACGACTCCAGCAGAAGCACAGCATTCTCTTGTCCA<br>CAGACACCAGCACCATACTCCAAAGTCATAAAATATGGAGTCTCATCCTACCT<br>GACCCTGCATACTCAAAGGCCCCTATGCACCCTGCTTTCCCCAGAGTCCATC<br>CTCCCATTCTGTTCTCCTGAGGACTCAGATACCAGGAAATACACAAGATACCA<br>TAGCAAGTTCCAGGACAACCAGGGCTACAGAGCAATCCTGTCTCAAACCAAA<br>CTGAGGTCCTGGGTAGGCCTCTGCTACCCTCTGGTGGCCTCAGGGTGCATA<br>GCACTACCAGGTGAAACAAACAAACAAAAAAATCTTTCTCCATTAGCATCTGA<br>GGGCTTAGATCACACAAGTGGGCCACAAGGCCCATCAAAGTGGCCTTATATC<br>ATGTGTGACATTCACTCCTGAGGCAGGTGTTCCCCAAAGGTTCAGCCCCACC<br>CTAGGATCACTGCTCCCCAGAGACATCTACAGGAAACAGCAGTCACAGAAAA<br>TAGTGAGGTGAGTTGCAGAGAATGGCCAGGGTGAGTCCTCACAGGGTTTCA<br>CACGGGCATGCCAGCAGTAAACTCTGATTTAAATCAGGTTCAGCAGGGAGTT<br>AGTTCGGAAGGAAACACCCTCCTCCCTGATCCCAGTCAAGACCAAGACAAGG<br>AAATGGAAGAGACATCAAAGCTTTATCAAAGGACTGTGACAGTGAAGTCAGG<br>CACAGTCCTAGCACAGATTCCTTCAAACAGCCTCTTAGTAAAGCCTTTCCAGA<br>CGTGGGGTAGGAAGTGAAAATAGCAGCCCAGACTCCTCAGAACACAAACTG<br>AGGTCCTGGGTAGGCCTCTGCTACCCTCTGGTGGCCTCAGGGTGCATAGCA<br>CTACCAGGTGTGACTGACAGCCCACTGCAAATTCATCCCCTACACCTTGAGA<br>AGCACCCCCACCACAACCAGCCTCTAATTGCCACCAGCCTTTGTCCTGACTC<br>AGGGCCCCCATCCAAGACTCAACCCTAGCCTGACACTGTAGGTCTTCTTAGG<br>AAAAGGACTCTAGGCCCAGCAACATAGGACTCCACACAAAGATCCCATGGTT<br>CCTAAGCCCCCAGGGGAAGGGATACAGGAACTGAACTGCCTTGGAAAACAG<br>GGCCCAGGCTTAGTGAGAGCAAAGCTGTCTGTACCTCCACCTGTGAGAAGC<br>CCCTGCCCCAGAGGCAGCACAGGGAATCCTGCCAGCCCAGGAAAAGCTAAT<br>GCCCTTCTGGCAAACATAAACCAGTCCCAGTCCTAGTCCTAAGGATGCCCAT<br>GGTGAGGAAGCCTGCAGTGCTTGCTAGTAAATCCCCCAAACCATGGCACAAA<br>GGTAAACAATGCTAAGAGCCTTTGAATCAGGGGCCACTAGGCAGGCAAACTG<br>AGGTCCTGGGTAGGCCTCTGCTACCCTCTGGTGGCCTCAGGGTGCATAGCA<br>CTACCAGGTG

Supplementary Table 4: smFISH probes used in this study

Name	Sequence (5’ to 3’)
Hba-a1/2 smFISH 1	CCATGGTTTCTTCCTGAG
Hba-a1/2 smFISH 2	TTTGTCTTCCCCAGAGAG
Hba-a1/2 smFISH 3	CAGGCAGCCTTGATGTTG
Hba-a1/2 smFISH 4	ACCATGGCCACCAATCTT
Hba-a1/2 smFISH 5	GCTTCAGCTCCATATTCA
Hba-a1/2 smFISH 6	TGGTGGGGAAGCTAGCAA
Hba-a1/2 smFISH 7	GTGAGGGAAGTAGGTCTT
Hba-a1/2 smFISH 8	AGAGCCGTGGCTTACATC
Hba-a1/2 smFISH 9	CGACCTTCTTGCCGTGAC
Hba-a1/2 smFISH 10	AGCATTGGCCAGAGCATC
Hba-a1/2 smFISH 11	GGCAGGTCATCGAGGTGG
Hba-a1/2 smFISH 12	TCAGAGCAGACAGGGCAC
Hba-a1/2 smFISH 13	CTTGTGGGCATGCAGGTC
Hba-a1/2 smFISH 14	TTGACGGGATCCACACGC
Hba-a1/2 smFISH 15	TCCCAGCGCATACCTTGA
Hba-a1/2 smFISH 16	GTGGCTAGCCAAGGTCAC
Hba-a1/2 smFISH 17	CGGGGGTGAAATCGGCAG
Hba-a1/2 smFISH 18	TGTCCAGAGAGGCATGCA
Hba-a1/2 smFISH 19	GCTCACAGAGGCAAGGAA
Hba-a1/2 smFISH 20	TACTTGGAGGTCAGCACG
Hba-a1/2 smFISH 21	CGCAGAAGGCAGCTTAAC
Hba-a1/2 smFISH 22	CATGGCCAGAAGGCAAGC
Hba-a1/2 smFISH 23	ACCAAGAGGTACAGGTGC
Hba-a1/2 smFISH 24	TCTTCCTACTCAGGCTTT
Hba-a1/2 smFISH 25	CAAGGTCCTGTTCTCACC
Hba-a1/2 smFISH 26	TCCTGTGATCCTTACAGA
Hba-a1/2 smFISH 27	AGTGCCAGGTCCATATTG
Hba-a1/2 smFISH 28	TTAGAAGCTGCCCACTGA
Hba-a1/2 smFISH 29	TCCTGGGAGAAGGAGAGA
Hba-a1/2 smFISH 30	TCCTAGGGGTCCCAGATG
Hba-a1/2 smFISH 31	TGCCTTGGGCACGAGGAC
Hba-a1/2 smFISH 32	CTTCCTGGGACCACTATG
Hba-a1/2 smFISH 33	GACACCCTGATGCCTCTG
Hba-a1/2 smFISH 34	GAGCTGCGGAGACAAAGT

Supplementary Table 5: Biotinylated capture probes sequences used in this study

Name	Sequence (5' to 3')
Biotinylated capture probe HS44 F	GATCTGCACCAGACATTCCTTCACAAGCACGGGTTAGAAGCCTAAGCTGGGAACAGCCAGTCCTCCCCTACCCACACTGCCCTGCCCTGTGGGAGAGGGCACTCGTCTTGGCTTAACTCT
Biotinylated capture probe HS44 R	GATCCAGGACTGCTCATGGCTTCAGTTCAGCTTTGTCACATCTTTGTCCTTACGTGGTATAAGAATCTCCCCTGGCACTCAAGAAGCTACCCAAGGGCAGTGAGGTGCCTTCTAGTCCCA
Biotinylated capture probe *Hba-1/Hba-2* promoter oligo1	GATCCATGGTAGCACAGGGCAGCTAAGATGCAAGTCTGAAGGAGGAGTCTGGCGAGCTGCTCCTGCAGTTCCCTGGACCCAGAAGGATGAGCTAGCAGATTCACTTGAGCCAAAGGATTC
Biotinylated capture probe *Hba-1/Hba-2* promoter oligo2	AAACCATGGTGCTCTCTGGGGAAGACAAAAGCAACATCAAGGCTGCCTGGGGGAAGATTGGTGGCCATGGTGCTGAATATGGAGCTGAAGCCCTGGAAAGGTGAGAACAGGACCTTGATC
Biotinylated capture probe of alpha-globin enhancer R2 oligo1	GATCTATGGAGATGCTTGAACGAGCAGATAACTAAGCCAAGCATGACTCAGAGTTTCTAGAGGCCACTAGGACTGCTGAGTAATACTTGGGGGTACAGAGTCAGAAAGGAAAGGACAAAT
Biotinylated capture probe of alpha-globin enhancer R2 oligo2	CTGAGAAAGCTGCCACCTAGAATGAGGCAGAGTTTAATGGGAATGCTAACAAAAAGTGTATTTACAGCTAGGTATGGTAGCTTACCCCTATAATGCCATCACTTGGGAGGTAGAAGGATC
