# Supplementary material for: Loop extrusion by cohesin plays a role in enhancer-activated gene expression early in differentiation
Source: Nat Commun. 2026 May 26;17:7297. doi: 10.1038/s41467-026-73049-5 (PMC13402345; doi:10.1038/s41467-026-73049-5)
Supplement: Supplementary file 3 — Reporting Summary [file 41467_2026_73049_MOESM3_ESM.pdf]

nature portfolio

Corresponding author(s): Doug Higgs and Mira Kassouf

Last updated by author(s): 14/03/2026

Reporting Summary

Nature Portfolio wishes to improve the reproducibility of the work that we publish. This form provides structure for consistency and transparency in reporting. For further information on Nature Portfolio policies, see our Editorial Policies and the Editorial Policy Checklist.

Statistics

For all statistical analyses, confirm that the following items are present in the figure legend, table legend, main text, or Methods section.

n/a	Confirmed	
☐	☒	The exact sample size (*n*) for each experimental group/condition, given as a discrete number and unit of measurement
☐	☒	A statement on whether measurements were taken from distinct samples or whether the same sample was measured repeatedly
☐	☒	The statistical test(s) used AND whether they are one- or two-sided<br>*Only common tests should be described solely by name; describe more complex techniques in the Methods section.*
☒	☐	A description of all covariates tested
☐	☒	A description of any assumptions or corrections, such as tests of normality and adjustment for multiple comparisons
☐	☒	A full description of the statistical parameters including central tendency (e.g. means) or other basic estimates (e.g. regression coefficient) AND variation (e.g. standard deviation) or associated estimates of uncertainty (e.g. confidence intervals)
☐	☒	For null hypothesis testing, the test statistic (e.g. *F*, *t*, *r*) with confidence intervals, effect sizes, degrees of freedom and *P* value noted<br>*Give P values as exact values whenever suitable.*
☒	☐	For Bayesian analysis, information on the choice of priors and Markov chain Monte Carlo settings
☒	☐	For hierarchical and complex designs, identification of the appropriate level for tests and full reporting of outcomes
☒	☐	Estimates of effect sizes (e.g. Cohen's *d*, Pearson's *r*), indicating how they were calculated

Our web collection on statistics for biologists contains articles on many of the points above.

Software and code

Policy information about availability of computer code

Data collection

Commercially available software was used for data collection:
- Sequencing: Sanger Sequencing (ABI-3730 DNA analyser), and NextSeq Platforms using the NextSeq System Suite (v2)
- FACS: Attune NxT software (v4.2.0)
- RT-qPCR: StepONEPlus Real-Time PCR system
- Digital droplet PCR: QuantaSoft AnalysisPro software
- RNA (prior to any expression analysis) and DNA quality (prior to ATAC, ChIP, Capture-C library preparations) assessment using TapeStation machine and software (Agilent).
- Western blot: iBright FL1000 scanner and software
- smFISH: Zeiss Efficient Navigation (blue edition) software (v2.6)

Data analysis

All tools used and mentioned here are referenced in the text and in the Methods section.
- ChIP-seq and ATAC-seq data was processed using trimgalore (v0.3.1), bowtie2 (v2.1.0), Samtools (version1.3), deepTools (v2.2.2), bedtools (v2.25.0).
- Chromosome Conformation Capture data was processed using a Capture-C pipeline within an in-house copy of the UpStreamPipeline suite (Riva et al., 2023), which incorporates the original Capture-C analyser scripts (Davies et al., 2016).
- FACS was analysed in FlowJo (v10.7).
- Western blot quantification was analyzed using ImageJ (v2.14.0)
- smFISH was analyzed using ImageJ (v2.14.0)

For manuscripts utilizing custom algorithms or software that are central to the research but not yet described in published literature, software must be made available to editors and reviewers. We strongly encourage code deposition in a community repository (e.g. GitHub). See the Nature Portfolio guidelines for submitting code & software for further information.

Data

Policy information about availability of data

All manuscripts must include a data availability statement. This statement should provide the following information, where applicable:

- Accession codes, unique identifiers, or web links for publicly available datasets
- A description of any restrictions on data availability
- For clinical datasets or third party data, please ensure that the statement adheres to our policy

All data generated for this study are included in this published article and its supplementary information. ChIP-seq, ATAC-seq and Capture-C data (raw and processed data) is available in the Gene Expression Omnibus (GEO) under accession numbers GSE163008, GSE163010, GSE163011, and GSE163012 or referenced if used from published data.

Human research participants

Policy information about studies involving human research participants and Sex and Gender in Research.

Reporting on sex and gender	*Use the terms sex (biological attribute) and gender (shaped by social and cultural circumstances) carefully in order to avoid confusing both terms. Indicate if findings apply to only one sex or gender; describe whether sex and gender were considered in study design whether sex and/or gender was determined based on self-reporting or assigned and methods used. Provide in the source data disaggregated sex and gender data where this information has been collected, and consent has been obtained for sharing of individual-level data; provide overall numbers in this Reporting Summary. Please state if this information has not been collected. Report sex- and gender-based analyses where performed, justify reasons for lack of sex- and gender-based analysis.*
Population characteristics	*Describe the covariate-relevant population characteristics of the human research participants (e.g. age, genotypic information, past and current diagnosis and treatment categories). If you filled out the behavioural & social sciences study design questions and have nothing to add here, write "See above."*
Recruitment	*Describe how participants were recruited. Outline any potential self-selection bias or other biases that may be present and how these are likely to impact results.*
Ethics oversight	*Identify the organization(s) that approved the study protocol.*

Note that full information on the approval of the study protocol must also be provided in the manuscript.

Field-specific reporting

Please select the one below that is the best fit for your research. If you are not sure, read the appropriate sections before making your selection.

☒ Life sciences ☐ Behavioural & social sciences ☐ Ecological, evolutionary & environmental sciences

For a reference copy of the document with all sections, see nature.com/documents/nr-reporting-summary-flat.pdf

Life sciences study design

All studies must disclose on these points even when the disclosure is negative.

Sample size	- For ChIP-seq, ATAC-seq, and NG Capture-C data, at least 2 independent replicates of each genotype were analysed.<br>- For RNA expression using RT-PCR and ddPCR, at least three independent replicates of each treatment group or genotype were analysed to allow statistical analysis (Student's t-test).<br>- For measuring nascent transcription using smFISH, at least three independent replicates of each treatment group were analysed to allow statistical analysis (Student's t-test).
Data exclusions	No data was excluded.
Replication	Data from primary cells derived from adult mouse tissues were reproducible in each genotype (blood parameters, spleen-derived data).<br>Data from engineered mouse Embryonic Stem Cell (ESC) lines were reproducible across experimental and biological replicas. Three single forward CTCF insertion and three single reverse CTCF insertion cell lines were originally generated and analysed to eliminate clonal variation and confirm that the phenotype is caused specifically by the engineered inversion. These produced identical phenotypes. The subsequently derived genetic models (multiple CTCF insertion models) were assessed similarly (independent clones when possible as well as experimental replicas).
Randomization	Randomisation was not applicable to this study as comparison was between distinct genotypes or treatment groups.
Blinding	Blinding was applied to quantify the number of active foci in the smFISH analysis. Image file name was masked prior to analysis, counts were made by an assessor blinded to treatment group allocation.

Reporting for specific materials, systems and methods

We require information from authors about some types of materials, experimental systems and methods used in many studies. Here, indicate whether each material, system or method listed is relevant to your study. If you are not sure if a list item applies to your research, read the appropriate section before selecting a response.

Materials & experimental systems

n/a	Involved in the study
☐	☒ Antibodies
☐	☒ Eukaryotic cell lines
☒	☐ Palaeontology and archaeology
☐	☒ Animals and other organisms
☒	☐ Clinical data
☒	☐ Dual use research of concern

Methods

n/a	Involved in the study
☐	☒ ChIP-seq
☐	☒ Flow cytometry
☒	☐ MRI-based neuroimaging

Antibodies

Antibodies used

ChIP:
CTCF Merck (Millipore 07-729, 5 μg/ml)
Rad21 Abcam (ab154769, 5 μg/ml)
Stag2 Bethyl (A302-580A, 5ug/ml)
Western Blot:
Mouse Anti-Flag (Sigma F1804)
Rabbit Anti-Beta actin (Abcam, ab115777)
IRDye 800CW goat anti-rabbit IgG (Abcam, ab216773)
IRDye 680RD goat anti-mouse igG (Abcam, ab216776)

Flow cytometry:
FITC Rat Anti-Mouse CD71 (eBioscience 11-0711-85 2.5 μg/ml)
PE Rat Anti-Mouse Ter119 (BD Pharmingen 553673 2 μg/ml)
Hoechst (Invitrogen H3569 1 μg/ml)

Validation

Data generated from all antibodies used in this study have been validated in various ways: at the basic level; by the manufacturers, and more thoroughly on our specific cell types as data has been produced (reproduced) in our laboratory by other scientists for other projects and by published literature by us and others on similar cell types. In some instances, the differences in phenotypes based on the genotype confirm specificity; when inserting CTCF sites for example, result in the addition of the relevant ChIP peak (CTCF, Rad21, Stag2 ChIP).

Eukaryotic cell lines

Policy information about cell lines and Sex and Gender in Research

Cell line source(s)

The wildtype E14TG2a mouse Embryonic Stem Cell (mESC) line was a kind gift from Prof Andrew Smith.

Authentication

In addition to the conventional tests (mESC colony morphology and alkaline phosphatase staining), the pluripotency of mESCs was verified in the in vitro hematopoietic differentiation assay which only produces embryoid bodies harbouring the three germ layers including the mesoderm that then produces erythroid cells if they were pluripotent. All the mESCs used passed this test. The CTCF-ins-F and CTCF-ins-R mESCs generated mouse models, the ultimate test of pluripotency. The Genetic engineering outcome was assessed using various methods include PCR genotyping, Sanger sequencing, and various genomics assays confirm the engineered modifications (ATAC-seq, ChIP-seq, Capture-C).

Mycoplasma contamination

Mycoplasma testing is regularly done within the tissue culture facility where these lines were produced, handled and analysed (MRC-MHU Weatherall Institute of Molecular Medicine)

Commonly misidentified lines
(See ICLAC register)

No commonly misidentified lines were used.

Animals and other research organisms

Policy information about studies involving animals; ARRIVE guidelines recommended for reporting animal research, and Sex and Gender in Research

Laboratory animals

C57BL/6J mice were sourced from MRC Harwell/Charles River Laboratories. All mouse work was performed in accordance with UK Home office regulations, under the appropriate animal licenses. Mouse model generation and animal husbandry was conducted by

	the Mouse Transgenics Core Facility at the Weatherall Institute of Molecular Medicine.
Wild animals	No wild animals were used in this study.
Reporting on sex	Sex was not relevant to this study however when adult mice were used we mixed the sex of the animals.
Field-collected samples	We had no field-collected samples.
Ethics oversight	No ethical approvals were needed for this study. We abide by the Home Office Regulations for the mouse work under the appropriate license.

Note that full information on the approval of the study protocol must also be provided in the manuscript.

ChIP-seq

Data deposition

☒ Confirm that both raw and final processed data have been deposited in a public database such as GEO.

☐ Confirm that you have deposited or provided access to graph files (e.g. BED files) for the called peaks.

Data access links
May remain private before publication.

Raw and processed files are available on the Gene Expression Omnibus (GEO) accession numbers GSE163012

Files in database submission

i-2_F_FL_CTCF_ChIP_chr11_RPKM_normalised.bw
FL_i-2_CTCF_ChIP_Fwd_R1.fastq.gz
FL_i-2_CTCF_ChIP_Fwd_R2.fastq.gz
5-1_F_FL_CTCF_ChIP_chr11_RPKM_normalised.bw
FL_5-1_CTCF_ChIP_R1.fastq.gz
FL_5-1_CTCF_ChIP_R2.fastq.gz
5-4_FL_F_CTCF_ChIP_chr11_RPKM_normalised.bw
FL_5-4_CTCF_ChIP_R1.fastq.gz
FL_5-4_CTCF_ChIP_R2.fastq.gz
f-3_FL_R_CTCF_ChIP_chr11_RPKM_normalised.bw
FL_f-3_CTCF_ChIP_Rev_R1.fastq.gz
FL_f-3_CTCF_ChIP_Rev_R2.fastq.gz
g-1_R_FL_CTCF_ChIP_chr11_RPKM_normalised.bw
FL_g-1_CTCF_ChIP_Rev_R1.fastq.gz
FL_g-1_CTCF_ChIP_Rev_R2.fastq.gz
g-4_R_FL_CTCF_ChIP_chr11_RPKM_normalised.bw
FL_g-4_CTCF_ChIP_Rev_R1.fastq.gz
FL_g-4_CTCF_ChIP_Rev_R2.fastq.gz
wtA_CTCF_ChIP_EB_chr11_RPKM_normalised.bw
wtA_CTCF_ChIP_EB_R1.fastq.gz
wtA_CTCF_ChIP_EB_R2.fastq.gz
wtB_CTCF_ChIP_EB_chr11_RPKM_normalised.bw
wtB_CTCF_ChIP_EB_R1.fastq.gz
wtB_CTCF_ChIP_EB_R2.fastq.gz
D2H3_EB_F_CTCF_ChIP_chr11_RPKM_normalised.bw
CTCF_ChIP_EB_D2H3_R1.fastq.gz
CTCF_ChIP_EB_D2H3_R2.fastq.gz
F2D3_Fwd_CTCF_ChIP_chr11_RPKM_normalised.bw
F2D3_Fwd_CTCF_ChIP_R1.fastq.gz
F2D3_Fwd_CTCF_ChIP_R2.fastq.gz
D2D6_EB_R_CTCF_ChIP_chr11_RPKM_normalised.bw
EB_CTCF_ChIP_Rev_D2D6_R1.fastq.gz
EB_CTCF_ChIP_Rev_D2D6_R2.fastq.gz
D3H2_EB_R_CTCF_ChIP_chr11_RPKM_normalised.bw
EB_CTCF_ChIP_D3H2_Rev_R1.fastq.gz
EB_CTCF_ChIP_D3H2_Rev_R2.fastq.gz
Rad21_ChIP_EB_Fwd_rep1_D2H3.bw
Rad21_ChIP_EB_Fwd_rep1_D2H3-i6_S3_L001_R1_001.fastq.gz
Rad21_ChIP_EB_Fwd_rep1_D2H3-i6_S3_L001_R2_001.fastq.gz
Rad21_ChIP_EB_Fwd_rep1_D2H3-i6_S3_L002_R1_001.fastq.gz
Rad21_ChIP_EB_Fwd_rep1_D2H3-i6_S3_L002_R2_001.fastq.gz
Rad21_ChIP_EB_Fwd_rep1_D2H3-i6_S3_L003_R1_001.fastq.gz
Rad21_ChIP_EB_Fwd_rep1_D2H3-i6_S3_L003_R2_001.fastq.gz
Rad21_ChIP_EB_Fwd_rep1_D2H3-i6_S3_L004_R1_001.fastq.gz
Rad21_ChIP_EB_Fwd_rep1_D2H3-i6_S3_L004_R2_001.fastq.gz
Rad21_ChIP_EB_Fwd_rep2_D3F3.bw
Rad21_ChIP_EB_Fwd_rep2_D3F3-i8_S4_L001_R1_001.fastq.gz

Rad21_ChIP_EB_Fwd_rep2_D3F3-i8_S4_L001_R2_001.fastq.gz
Rad21_ChIP_EB_Fwd_rep2_D3F3-i8_S4_L002_R1_001.fastq.gz
Rad21_ChIP_EB_Fwd_rep2_D3F3-i8_S4_L002_R2_001.fastq.gz
Rad21_ChIP_EB_Fwd_rep2_D3F3-i8_S4_L003_R1_001.fastq.gz
Rad21_ChIP_EB_Fwd_rep2_D3F3-i8_S4_L003_R2_001.fastq.gz
Rad21_ChIP_EB_Fwd_rep2_D3F3-i8_S4_L004_R1_001.fastq.gz
Rad21_ChIP_EB_Fwd_rep2_D3F3-i8_S4_L004_R2_001.fastq.gz
Rad21_ChIP_EB_Fwd_rep3_F2D3.bw
Rad21_ChIP_EB_Fwd_rep3_F2D3-i4_S2_L001_R1_001.fastq.gz
Rad21_ChIP_EB_Fwd_rep3_F2D3-i4_S2_L001_R2_001.fastq.gz
Rad21_ChIP_EB_Fwd_rep3_F2D3-i4_S2_L002_R1_001.fastq.gz
Rad21_ChIP_EB_Fwd_rep3_F2D3-i4_S2_L002_R2_001.fastq.gz
Rad21_ChIP_EB_Fwd_rep3_F2D3-i4_S2_L003_R1_001.fastq.gz
Rad21_ChIP_EB_Fwd_rep3_F2D3-i4_S2_L003_R2_001.fastq.gz
Rad21_ChIP_EB_Fwd_rep3_F2D3-i4_S2_L004_R1_001.fastq.gz
Rad21_ChIP_EB_Fwd_rep3_F2D3-i4_S2_L004_R2_001.fastq.gz
Rad21_ChIP_EB_Rev_rep1_D2D6.bw
Rad21_ChIP_EB_Rev_rep1_D2D6-i1_S1_L001_R1_001.fastq.gz
Rad21_ChIP_EB_Rev_rep1_D2D6-i1_S1_L001_R2_001.fastq.gz
Rad21_ChIP_EB_Rev_rep1_D2D6-i1_S1_L002_R1_001.fastq.gz
Rad21_ChIP_EB_Rev_rep1_D2D6-i1_S1_L002_R2_001.fastq.gz
Rad21_ChIP_EB_Rev_rep1_D2D6-i1_S1_L003_R1_001.fastq.gz
Rad21_ChIP_EB_Rev_rep1_D2D6-i1_S1_L003_R2_001.fastq.gz
Rad21_ChIP_EB_Rev_rep1_D2D6-i1_S1_L004_R1_001.fastq.gz
Rad21_ChIP_EB_Rev_rep1_D2D6-i1_S1_L004_R2_001.fastq.gz
Rad21_ChIP_EB_Rev_rep2_D3H2.bw
Rad21_ChIP_EB_Rev_rep2_D3H2-i2_S13_L001_R1_001.fastq.gz
Rad21_ChIP_EB_Rev_rep2_D3H2-i2_S13_L001_R2_001.fastq.gz
Rad21_ChIP_EB_Rev_rep2_D3H2-i2_S13_L002_R1_001.fastq.gz
Rad21_ChIP_EB_Rev_rep2_D3H2-i2_S13_L002_R2_001.fastq.gz
Rad21_ChIP_EB_Rev_rep2_D3H2-i2_S13_L003_R1_001.fastq.gz
Rad21_ChIP_EB_Rev_rep2_D3H2-i2_S13_L003_R2_001.fastq.gz
Rad21_ChIP_EB_Rev_rep2_D3H2-i2_S13_L004_R1_001.fastq.gz
Rad21_ChIP_EB_Rev_rep2_D3H2-i2_S13_L004_R2_001.fastq.gz
Rad21_ChIP_EB_Rev_rep3_F2G6.bw
Rad21_ChIP_EB_Rev_rep3_F2G6-i7_S15_L001_R1_001.fastq.gz
Rad21_ChIP_EB_Rev_rep3_F2G6-i7_S15_L001_R2_001.fastq.gz
Rad21_ChIP_EB_Rev_rep3_F2G6-i7_S15_L002_R1_001.fastq.gz
Rad21_ChIP_EB_Rev_rep3_F2G6-i7_S15_L002_R2_001.fastq.gz
Rad21_ChIP_EB_Rev_rep3_F2G6-i7_S15_L003_R1_001.fastq.gz
Rad21_ChIP_EB_Rev_rep3_F2G6-i7_S15_L003_R2_001.fastq.gz
Rad21_ChIP_EB_Rev_rep3_F2G6-i7_S15_L004_R1_001.fastq.gz
Rad21_ChIP_EB_Rev_rep3_F2G6-i7_S15_L004_R2_001.fastq.gz
Rad21_ChIP_Spleen_Fwd_rep1.bw
Rad21_ChIP_Spleen_Fwd_rep1-i20_S7_L001_R1_001.fastq.gz
Rad21_ChIP_Spleen_Fwd_rep1-i20_S7_L001_R2_001.fastq.gz
Rad21_ChIP_Spleen_Fwd_rep1-i20_S7_L002_R1_001.fastq.gz
Rad21_ChIP_Spleen_Fwd_rep1-i20_S7_L002_R2_001.fastq.gz
Rad21_ChIP_Spleen_Fwd_rep1-i20_S7_L003_R1_001.fastq.gz
Rad21_ChIP_Spleen_Fwd_rep1-i20_S7_L003_R2_001.fastq.gz
Rad21_ChIP_Spleen_Fwd_rep1-i20_S7_L004_R1_001.fastq.gz
Rad21_ChIP_Spleen_Fwd_rep1-i20_S7_L004_R2_001.fastq.gz
Rad21_ChIP_Spleen_Fwd_rep2.bw
Rad21_ChIP_Spleen_Fwd_rep2-i21_S8_L001_R1_001.fastq.gz
Rad21_ChIP_Spleen_Fwd_rep2-i21_S8_L001_R2_001.fastq.gz
Rad21_ChIP_Spleen_Fwd_rep2-i21_S8_L002_R1_001.fastq.gz
Rad21_ChIP_Spleen_Fwd_rep2-i21_S8_L002_R2_001.fastq.gz
Rad21_ChIP_Spleen_Fwd_rep2-i21_S8_L003_R1_001.fastq.gz
Rad21_ChIP_Spleen_Fwd_rep2-i21_S8_L003_R2_001.fastq.gz
Rad21_ChIP_Spleen_Fwd_rep2-i21_S8_L004_R1_001.fastq.gz
Rad21_ChIP_Spleen_Fwd_rep2-i21_S8_L004_R2_001.fastq.gz
Rad21_ChIP_Spleen_Fwd_rep3.bw
Rad21_ChIP_Spleen_Fwd_rep3-i22_S9_L001_R1_001.fastq.gz
Rad21_ChIP_Spleen_Fwd_rep3-i22_S9_L001_R2_001.fastq.gz
Rad21_ChIP_Spleen_Fwd_rep3-i22_S9_L002_R1_001.fastq.gz
Rad21_ChIP_Spleen_Fwd_rep3-i22_S9_L002_R2_001.fastq.gz
Rad21_ChIP_Spleen_Fwd_rep3-i22_S9_L003_R1_001.fastq.gz
Rad21_ChIP_Spleen_Fwd_rep3-i22_S9_L003_R2_001.fastq.gz
Rad21_ChIP_Spleen_Fwd_rep3-i22_S9_L004_R1_001.fastq.gz

Rad21_ChIP_Spleen_Fwd_rep3-i22_S9_L004_R2_001.fastq.gz
Rad21_ChIP_Spleen_Rev_rep1.bw
Rad21_ChIP_Spleen_Rev_rep1-i23_S10_L001_R1_001.fastq.gz
Rad21_ChIP_Spleen_Rev_rep1-i23_S10_L001_R2_001.fastq.gz
Rad21_ChIP_Spleen_Rev_rep1-i23_S10_L002_R1_001.fastq.gz
Rad21_ChIP_Spleen_Rev_rep1-i23_S10_L002_R2_001.fastq.gz
Rad21_ChIP_Spleen_Rev_rep1-i23_S10_L003_R1_001.fastq.gz
Rad21_ChIP_Spleen_Rev_rep1-i23_S10_L003_R2_001.fastq.gz
Rad21_ChIP_Spleen_Rev_rep1-i23_S10_L004_R1_001.fastq.gz
Rad21_ChIP_Spleen_Rev_rep1-i23_S10_L004_R2_001.fastq.gz
Rad21_ChIP_Spleen_Rev_rep2.bw
Rad21_ChIP_Spleen_Rev_rep2-i25_S11_L001_R1_001.fastq.gz
Rad21_ChIP_Spleen_Rev_rep2-i25_S11_L001_R2_001.fastq.gz
Rad21_ChIP_Spleen_Rev_rep2-i25_S11_L002_R1_001.fastq.gz
Rad21_ChIP_Spleen_Rev_rep2-i25_S11_L002_R2_001.fastq.gz
Rad21_ChIP_Spleen_Rev_rep2-i25_S11_L003_R1_001.fastq.gz
Rad21_ChIP_Spleen_Rev_rep2-i25_S11_L003_R2_001.fastq.gz
Rad21_ChIP_Spleen_Rev_rep2-i25_S11_L004_R1_001.fastq.gz
Rad21_ChIP_Spleen_Rev_rep2-i25_S11_L004_R2_001.fastq.gz
Rad21_ChIP_Spleen_Rev_rep3.bw
Rad21_ChIP_Spleen_Rev_rep3-i20_S5_L001_R1_001.fastq.gz
Rad21_ChIP_Spleen_Rev_rep3-i20_S5_L001_R2_001.fastq.gz
Rad21_ChIP_Spleen_Rev_rep3-i20_S5_L002_R1_001.fastq.gz
Rad21_ChIP_Spleen_Rev_rep3-i20_S5_L002_R2_001.fastq.gz
Rad21_ChIP_Spleen_Rev_rep3-i20_S5_L003_R1_001.fastq.gz
Rad21_ChIP_Spleen_Rev_rep3-i20_S5_L003_R2_001.fastq.gz
Rad21_ChIP_Spleen_Rev_rep3-i20_S5_L004_R1_001.fastq.gz
Rad21_ChIP_Spleen_Rev_rep3-i20_S5_L004_R2_001.fastq.gz
Stag2_ChIP_Spleen_Fwd_rep1.bw
Stag2_ChIP_Spleen_Fwd_rep1-i11_S2_L001_R1_001.fastq.gz
Stag2_ChIP_Spleen_Fwd_rep1-i11_S2_L001_R2_001.fastq.gz
Stag2_ChIP_Spleen_Fwd_rep1-i11_S2_L002_R1_001.fastq.gz
Stag2_ChIP_Spleen_Fwd_rep1-i11_S2_L002_R2_001.fastq.gz
Stag2_ChIP_Spleen_Fwd_rep1-i11_S2_L003_R1_001.fastq.gz
Stag2_ChIP_Spleen_Fwd_rep1-i11_S2_L003_R2_001.fastq.gz
Stag2_ChIP_Spleen_Fwd_rep1-i11_S2_L004_R1_001.fastq.gz
Stag2_ChIP_Spleen_Fwd_rep1-i11_S2_L004_R2_001.fastq.gz
Stag2_ChIP_Spleen_Fwd_rep2.bw
Stag2_ChIP_Spleen_Fwd_rep2-i12_S5_L001_R1_001.fastq.gz
Stag2_ChIP_Spleen_Fwd_rep2-i12_S5_L001_R2_001.fastq.gz
Stag2_ChIP_Spleen_Fwd_rep2-i12_S5_L002_R1_001.fastq.gz
Stag2_ChIP_Spleen_Fwd_rep2-i12_S5_L002_R2_001.fastq.gz
Stag2_ChIP_Spleen_Fwd_rep2-i12_S5_L003_R1_001.fastq.gz
Stag2_ChIP_Spleen_Fwd_rep2-i12_S5_L003_R2_001.fastq.gz
Stag2_ChIP_Spleen_Fwd_rep2-i12_S5_L004_R1_001.fastq.gz
Stag2_ChIP_Spleen_Fwd_rep2-i12_S5_L004_R2_001.fastq.gz
Stag2_ChIP_Spleen_Fwd_rep3.bw
Stag2_ChIP_Spleen_Fwd_rep3-i13_S6_L001_R1_001.fastq.gz
Stag2_ChIP_Spleen_Fwd_rep3-i13_S6_L001_R2_001.fastq.gz
Stag2_ChIP_Spleen_Fwd_rep3-i13_S6_L002_R1_001.fastq.gz
Stag2_ChIP_Spleen_Fwd_rep3-i13_S6_L002_R2_001.fastq.gz
Stag2_ChIP_Spleen_Fwd_rep3-i13_S6_L003_R1_001.fastq.gz
Stag2_ChIP_Spleen_Fwd_rep3-i13_S6_L003_R2_001.fastq.gz
Stag2_ChIP_Spleen_Fwd_rep3-i13_S6_L004_R1_001.fastq.gz
Stag2_ChIP_Spleen_Fwd_rep3-i13_S6_L004_R2_001.fastq.gz
Stag2_ChIP_Spleen_Rev_rep1.bw
Stag2_ChIP_Spleen_Rev_rep1-i8_S1_L001_R1_001.fastq.gz
Stag2_ChIP_Spleen_Rev_rep1-i8_S1_L001_R2_001.fastq.gz
Stag2_ChIP_Spleen_Rev_rep1-i8_S1_L002_R1_001.fastq.gz
Stag2_ChIP_Spleen_Rev_rep1-i8_S1_L002_R2_001.fastq.gz
Stag2_ChIP_Spleen_Rev_rep1-i8_S1_L003_R1_001.fastq.gz
Stag2_ChIP_Spleen_Rev_rep1-i8_S1_L003_R2_001.fastq.gz
Stag2_ChIP_Spleen_Rev_rep1-i8_S1_L004_R1_001.fastq.gz
Stag2_ChIP_Spleen_Rev_rep1-i8_S1_L004_R2_001.fastq.gz
Stag2_ChIP_Spleen_Rev_rep2.bw
Stag2_ChIP_Spleen_Rev_rep2-i9_S3_L001_R1_001.fastq.gz
Stag2_ChIP_Spleen_Rev_rep2-i9_S3_L001_R2_001.fastq.gz
Stag2_ChIP_Spleen_Rev_rep2-i9_S3_L002_R1_001.fastq.gz
Stag2_ChIP_Spleen_Rev_rep2-i9_S3_L002_R2_001.fastq.gz

Stag2_ChIP_Spleen_Rev_rep2-i9_S3_L003_R1_001.fastq.gz
Stag2_ChIP_Spleen_Rev_rep2-i9_S3_L003_R2_001.fastq.gz
Stag2_ChIP_Spleen_Rev_rep2-i9_S3_L004_R1_001.fastq.gz
Stag2_ChIP_Spleen_Rev_rep2-i9_S3_L004_R2_001.fastq.gz
Stag2_ChIP_Spleen_Rev_rep3.bw
Stag2_ChIP_Spleen_Rev_rep3-i10_S4_L001_R1_001.fastq.gz
Stag2_ChIP_Spleen_Rev_rep3-i10_S4_L001_R2_001.fastq.gz
Stag2_ChIP_Spleen_Rev_rep3-i10_S4_L002_R1_001.fastq.gz
Stag2_ChIP_Spleen_Rev_rep3-i10_S4_L002_R2_001.fastq.gz
Stag2_ChIP_Spleen_Rev_rep3-i10_S4_L003_R1_001.fastq.gz
Stag2_ChIP_Spleen_Rev_rep3-i10_S4_L003_R2_001.fastq.gz
Stag2_ChIP_Spleen_Rev_rep3-i10_S4_L004_R1_001.fastq.gz
Stag2_ChIP_Spleen_Rev_rep3-i10_S4_L004_R2_001.fastq.gz

Genome browser session
(e.g. UCSC)

Methodology

Replicates

At least two replicates were performed per ChIP as indicated in the text.

Sequencing depth

CTCF_ChIP_FL_Fwd_rep1_i2: 63666862 total reads; 57962300 mapped reads
CTCF_ChIP_FL_Fwd_rep2_5-1: 156045347 total reads; 143502253 mapped reads
CTCF_ChIP_FL_Fwd_rep3_5-4: 48467872 total reads; 42894467 mapped reads
CTCF_ChIP_FL_Rev_rep1_f3: 27781895 total reads; 25562277 mapped reads
CTCF_ChIP_FL_Rev_rep2_g-1: 155573567 total reads; 140968147 mapped reads
CTCF_ChIP_FL_Rev_rep3_g-4: 30837070 total reads; 28215797 mapped reads
CTCF_ChIP_EB_WT_rep1: 36237835 total reads; 33228528 mapped reads
CTCF_ChIP_EB_WT_rep2: 31741068 total reads; 28740977 mapped reads
CTCF_ChIP_EB_Fwd_rep1_D2H3: 20673252 total reads; 19397506 mapped reads
CTCF_ChIP_EB_Fwd_rep2_F2D3: 20093604 total reads; 15695369 mapped reads
CTCF_ChIP_EB_Rev_rep1_D2D6: 22032198 total reads; 20582180 mapped reads
CTCF_ChIP_EB_Rev_rep2_D3H2: 73107096 total reads; 48958559 mapped reads

Rad21_ChIP_EB_Fwd_rep1_D2H3: 53592844 total reads; 49381711 mapped reads
Rad21_ChIP_EB_Fwd_rep2_D3F3: 52824104 total reads; 50944873 mapped reads
Rad21_ChIP_EB_Fwd_rep3_F2D3: 56892994 total reads; 54591658 mapped reads
Rad21_ChIP_EB_Rev_rep1_D2D6: 33294076 total reads; 31687266 mapped reads
Rad21_ChIP_EB_Rev_rep2_D3H2: 34826824 total reads; 33806025 mapped reads
Rad21_ChIP_EB_Rev_rep3_F2G6: 22615295 total reads; 21904768 mapped reads

Rad21_ChIP_Spleen_Fwd_rep1: 44645441 total reads; 34214471 mapped reads
Rad21_ChIP_Spleen_Fwd_rep2: 48803213 total reads; 40435940 mapped reads
Rad21_ChIP_Spleen_Fwd_rep3: 48652736 total reads; 45574172 mapped reads
Rad21_ChIP_Spleen_Rev_rep1: 45475364 total reads; 43183459 mapped reads
Rad21_ChIP_Spleen_Rev_rep2: 50417166 total reads; 46325379 mapped reads
Rad21_ChIP_Spleen_Rev_rep3: 32870523 total reads; 30629094 mapped reads

Stag2_ChIP_Spleen_Fwd_rep1: 6835179 total reads; 5672127 mapped reads
Stag2_ChIP_Spleen_Fwd_rep2: 16521527 total reads; 15532536 mapped reads
Stag2_ChIP_Spleen_Fwd_rep3: 17459964 total reads; 16159480 mapped reads
Stag2_ChIP_Spleen_Rev_rep1: 11215756 total reads; 10065171 mapped reads
Stag2_ChIP_Spleen_Rev_rep2: 16306038 total reads; 15129505 mapped reads
Stag2_ChIP_Spleen_Rev_rep3: 16784048 total reads; 15920950 mapped reads

Antibodies

Antibodies and concentrations used as specified in the antibody field above.

Peak calling parameters

Bigwig coverage tracks for CTCF and Rad21 ChIP-seq were used as inputs for LanceOtron calling peaks using default settings. The resulting peak regions were filtered with scores between 0.8 to 1. Bedtools multicov was used to count reads over the peak call list for every sample and the average number of reads per peak was used to normalise read counts over the ectopic CTCF site.

Data quality

ChIP quality was assessed in two ways; ChIP material was tested by qPCR prior to sequencing to ensure a good fold-enrichment at a relevant (expected positive) sequence over a negative region. Replicates were also checked for consistency. For the erythroid populations, we also checked that WT and engineered cells tested were yielding the expected ChIP profile over well-characterised unaffected regions (the beta globin locus).

Software

Sequencing data was collected using NextSeq Illumina platforms and mapped using bowtie2.

Flow Cytometry

Plots

Confirm that:

☒ The axis labels state the marker and fluorochrome used (e.g. CD4-FITC).

☒ The axis scales are clearly visible. Include numbers along axes only for bottom left plot of group (a 'group' is an analysis of identical markers).

☒ All plots are contour plots with outliers or pseudocolor plots.

☒ A numerical value for number of cells or percentage (with statistics) is provided.

Methodology

Sample preparation	Minimum of 10^5 cells were collected per sample. Cells will spun down and resuspended in PBS with 10%FBS (200 µl) and antibodies added at concentrations set above in antibody section, incubated at 4 oC for 20 minutes then washed once and resuspended in 500 µl of PBS with 10%FBS with Hoechst.
Instrument	Attune NxT Flow Cytometer
Software	Data collection: Attune NxT software (v3.0)<br>Data analysis: FlowJo v10.7
Cell population abundance	Analysis of erythroid cell populations was done based on CD71/ter119 conventional analysis with the expected population frequency from the various cell populations analysed (consitent with published data by us and others, referenced in the text).
Gating strategy	Voltages and compensations were set using unstained and single fluorophore-stained samples. Negative and positive populations were established following the software's criteria and the following plots: Forward and Side Scatter (to gate the live and right size populations), doublet exclusion was done by gating single cells using FCS-area and FCS-height, and live cells were selected using the Hoechst negative gate. The main fluorophore compensation was done between Ter119-PE and CD71-FITC.

☒ Tick this box to confirm that a figure exemplifying the gating strategy is provided in the Supplementary Information.
