# Supplementary material for: Loop extrusion by cohesin plays a role in enhancer-activated gene expression early in differentiation
Source: Nat Commun. 2026 May 26;17:7297. doi: 10.1038/s41467-026-73049-5 (PMC13402345; doi:10.1038/s41467-026-73049-5)
Supplement: Supplementary file 2 — Transparent Peer Review file [file 41467_2026_73049_MOESM2_ESM.pdf]

nature portfolio

Peer Review File

Loop extrusion by cohesin plays a role in enhancer-activated gene expression early in differentiation

Corresponding Author: Professor Douglas Higgs

This manuscript has been previously reviewed at another journal. This document only contains information relating to versions considered at Nature Communications.

This file contains all reviewer reports in order by version, followed by all author rebuttals in order by version.

Version 1:

Reviewer comments:

Reviewer #2

(Remarks to the Author)
The authors have worked hard to address all the comments, and the manuscript is improved. I have a few suggestions that require no experimentation but should be considered in the text.

1. I suggest that the title and main conclusions should be modified to represent what is actually happening. Cohesin extrusion plays a small (not “key”) role in undifferentiated cells, and no role at later stages when alpha transcription is high. This is consistent with capture-c showing no statistically significant architectural effects upon Rad21 degradation. Alpha expression is not “disrupted” but subtly decreased and only early on. Taking a step back, a title more representative of the observations might read: “Enhancer-activated gene expression is largely independent of cohesin during differentiation”.
2. Regarding my previous point 2, I am afraid I was misunderstood. I was not suggesting to deplete cohesin for 3 days. As stated previously, "maintenance" of loops through later stages does not make sense as an explanation here. To the uninitiated reader, “maintenance” could be misinterpreted to mean that loops are stable for long periods of time. Cells have divided and have not "maintained" any loops. Loops are of short duration and are dissolved and established continuously. Plus, alpha expression increases throughout differentiation presumably accompanied by further increases in contacts. Together, this means that loops are formed de novo by factors other than cohesin.
3. There are no significant architectural changes in Rad21 k.o. cells. The authors state that there are trends, but they are minor and quite similar in early and late cells when there is no effect on gene expression. The inevitable noise in the data and lack of information regarding replicate concordance precludes any firm conclusions here. Again, this suggests that cohesin may mildly influence something else (i.e. TF occupancy) which should be considered.
4. Previous point 3: please show the Rad21 ChIP tracks at the alpha locus -/+ dTag ligand at day 5 vs day 7. This is essential for any reader trying to interpret cohesin’s role.
5. Please reconcile in the Discussion why in day 7 EBs Rad21 degradation has no effect on alpha expression but insertion of a CTCF site does. Does CTCF function as an insulator independently of Rad21 here?
6. Related to point 5 (Line 521):” …insertion of multiple CTCF binding sites further reduced the alpha globin gene expression by imposing further resistance to the extruding cohesin” Is there a correlation between the number of inserted CTCF sites and Rad21 accumulation? The Rad 12 signal seems already very high with a single site.

Minor:
Supplemental Fig.11 in foetal cells: are the gene expression changes meaningful? Looking at the y-axis the signal seems very low, similar to suppl Fig. 10c where low signals were non-significant. This could just be noise around the baseline.

Reviewer #3

(Remarks to the Author)
This paper focuses on the role the cohesin/loop extrusion on the alpha-globin gene regulation during erythroid differentiation. The alpha-globin locus was used as a model locus to dissect principles of gene expression, with a strong

CTCF binding site used as a tool to block loop extrusion and to evaluate its role on gene expression.

Major findings of this paper are the demonstration of a role for loop extrusion at relative short distances between enhancers and promoters, in the range of 30-50 kb, together with the fact that cohesin may play a role in establishing enhancer-promoter interactions rather than maintaining them during differentiation. These features may reconcile many previously published data reporting weak functional role of cohesin/Rad21 depletion, most of the time in steady-state conditions.

Of note, this is a second round of review (I was not included in the first round), and I have been asking to further comments on the author responses to initial reviewers. In general, I found experiments well carried out and results convincing. Additionally, I found their responses to initial reviews rather convincing.

I agree with reviewer one that a more direct system could have been used, such as the one used by Hansen et al (Science 2025), i.e. acute depletion of NIPBL, which disrupts long-range cohesin-dependent interactions without compromising cell division.
However, I still think that the authors used here an elegant strategy to dissect the mechanism of loop extrusion dependency at the alpha-globin genes, i.e. by using a strong CTCF binding site to efficiently block loop extrusion between enhancers and promoters at the alpha-globin locus. Their new Rad21 ChIP-seq analysis convincingly indicates a stalled cohesin at the ectopic CTCF site between the enhancer and the promoter sites. Interestingly, the transcriptional effect seems to correlate with the degree of cohesin stalling.

Additional points to consider:
- The authors should definitely refer to the recent Hansen et al paper in their main text. Not sure it is the case at the moment.

- Since the enhancer-promoter distance is an important criterion in defining the loop extrusion dependency (over just 20 kb to require cohesin function, see Hansen et al.), the authors could also discuss this aspect. They should also indicate more precisely distances (for instance in legend Figure 2) between the enhancers, the ectopic CTCF site and the Hba-a promoters.
Actually, no functional effect is detected for Hba-x, which could be due to a too short distance from the enhancer (< to 20 kb) to be sensitive to cohesin depletion. It is also possible that this distance threshold could explain why the functional effect is more robust on Hbb compare to Hba, specially at the latest stage of differentiation in the EB model. I recommend the authors to include this in their discussion.

- For the ChIP-seq quantification of Fig.4, I found a bit weird their choice of tissues, i.e. mouse spleen derived erythroid cells for the Rad21 ChIP-seq and fetal liver for the CTCF ChIP-seq. It would be nice to homogenize the data in the same tissue.

- In Fig.6 and for the sake of clarity, the authors should indicate which additional peaks they are referring to in the text.

Open Access This Peer Review File is licensed under a Creative Commons Attribution 4.0 International License, which permits use, sharing, adaptation, distribution and reproduction in any medium or format, as long as you give appropriate credit to the original author(s) and the source, provide a link to the Creative Commons license, and indicate if changes were made.
In cases where reviewers are anonymous, credit should be given to 'Anonymous Referee' and the source.

The images or other third party material in this Peer Review File are included in the article's Creative Commons license, unless indicated otherwise in a credit line to the material. If material is not included in the article's Creative Commons license and your intended use is not permitted by statutory regulation or exceeds the permitted use, you will need to obtain permission directly from the copyright holder.
To view a copy of this license, visit https://creativecommons.org/licenses/by/4.0/

RESPONSE TO REVIEWERS' COMMENTS

Reviewer #1 (Remarks to the Author):

This study by Stolper and colleagues addresses the role of cohesin loop-extrusion in activation of transcription by distal enhancers. The authors show that during both in vivo and in vitro differentiation, the expression of alpha-globin genes is sensitive to cohesin depletion. Interestingly, loss of Cohesin has a stronger impact during the earliest stages of differentiation compared to the later ones. The authors then go on to show that insertion of CTCF motifs between the alpha-globin genes and their enhancers can recruit cohesin, reduce enhancer-promoter contacts, and affect gene activation. Importantly, CTCF motifs pointing towards the enhancer did all these processes more efficiently than motifs pointing towards the alpha-globin genes, suggesting that cohesin retention, which may be higher for complexes extruding from the enhancer side, blocks the ability of enhancers to activate alpha-globin. **The data presented are of high-quality. Many conclusions are well supported by evidence.** But the most important messages in this study are either not novel, or only indirect evidence support the interpretations that the authors favour.

While Figure 1 directly addresses the importance of Cohesin in gene activation by depleting cohesin, the rest of the study uses cohesin retention by CTCF between an enhancer and a promoter to demonstrate that cohesin loop extrusion is required for enhancer-promoter contacts. I find this approach rather indirect. Given the importance the authors give to the role of Cohesin in contributing to enhancer-promoter contacts, it is a little strange that the approach and observations made in Figure 1 are quickly abandoned.

We thank the reviewer for concluding that "The data presented are of high-quality" and that "Many conclusions are well supported by evidence". The reviewer raises several important and interesting points. First, we understand that it is important not to bias our interpretation of the data and to address other possible explanations of our new findings. In response, we have revised the manuscript to comment more extensively on the current models addressing how enhancers may come into close proximity to promoters.

The possible mechanisms have been very well set out in the review from Anders Hansen[1]. These involve thermal diffusion, protein-protein interactions, formation of transcriptional hubs and loop extrusion: the importance of each mechanism is still uncertain and being currently explored by many groups. It seems likely that several mechanisms may act together or sequentially to increase the *probability* of an interaction. Furthermore, the contribution of each of these factors may change as cells go through development and differentiation. Here we have focussed only on the potential importance of loop extrusion at a very well characterised mammalian locus during the process of differentiation which, from previous experience, has enabled us to establish principles of gene regulation that apply to many other genes.

We have performed an acute (6hours) depletion of Rad21 to test its primary function with least secondary effects and observed that cohesin, and potentially loop extrusion, play a role in enhancer-driven gene expression at the alpha-globin locus. In terms of novelty, we have shown, we think for the first time, that cohesin mediated loop extrusion plays a role in establishing rather than maintaining enhancer-promoter contact during differentiation. Others including the lab of Matthias Merkenschlager have discussed this before[2] but have nor directly tested this. Having shown this, it next seemed logical to ask what would happen to gene expression if we interrupted loop extrusion using intervening CTCF binding sites, a well-documented property for CTCF. We found that gene expression was reduced consistent with loop extrusion playing a role in enhancer-promoter interaction at this locus. In addition, we found that loop extrusion occurred in a manner consistent with cohesin predominantly translocating from the enhancer side to the promoter side of the locus. It should be stressed that, these experiments were undertaken in a well-characterised and tractable model using small (83bp) inserts that contain one CTCF site as opposed to many previous studies that have used a similar approach with larger (1 to 4 Kb) fragments containing many potentially functional motifs (e.g.Huang et al, 2021). We also show that cohesin-mediated loop extrusion is required for enhancer-promoter interaction over relatively short distances, in contrast to previous assertions[3].

Finally, it would be of interest to know which experiments the reviewer would consider to be a more “direct approach” so that this could be discussed in a revised text.

The experiments in figures 2-6 show that CTCF insertions retained more cohesin when pointing towards the enhancer. This makes sense assuming there is more deposition on that side–this is not shown however.

Evidence of the deposition of cohesin on enhancers has been recently reported by Rinzema N et al.[3]. In our own data, we have included a quantitative evaluation of the Rad21 and CTCF ChIP reads over the CTCF insertion site in both orientations and shown that cohesin accumulates more when CTCF is pointing towards the enhancers in definitive erythroid cells (mouse spleen data) (Fig4b and c). Interestingly, when examined closely, Rad21 ChIP-seq shows that reads/peaks are skewed to the right (promoter side) or left (enhancer side) depending on the inserted CTCF site orientation (forward or reverse respectively) (Supp. Fig 9. These observations agree with reported findings (Li et al., Nature 2020 and Pugacheva et al., PNAS 2020) showing that blocking of cohesin is mediated by the N-terminal domain of the CTCF protein[4,5].

Supplementary Fig. 9

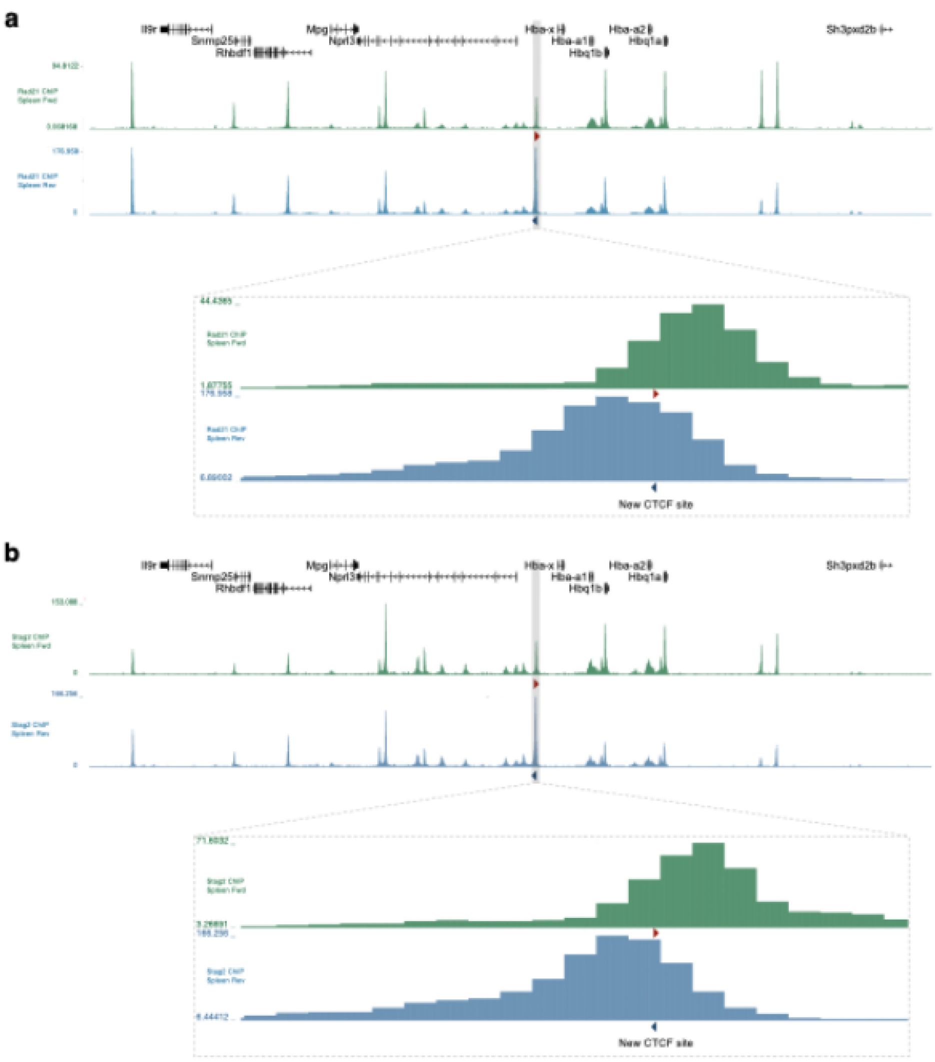

More cohesin retention at the CTCF insertion also led to less frequent enhancer-promoter contacts. The authors directly interpret the fact that cohesin retention at CTCF, along with lower expression, means that cohesin is required to activate those promoters. The data do show that retention of cohesin at CTCF reduces enhancer-promoter frequency but cohesin-independent mechanisms could still be responsible for the enhancer-promoter contacts.

Ocam’s razor would suggest that if a reduction in cohesin leads to reduced expression and the translocation of cohesin is partially blocked at intervening CTCF sites, and that this is associated with a reduction in gene expression that these phenomena are causally related. Of course, other interpretations could explain this. Furthermore, if a tracking mechanism such as loop extrusion is not involved in the observed reduction of gene expression, it is difficult to understand why the orientation of the inserted CTCF site is important.

For example, it is possible that proteins could bind to both enhancers and promoters and induce contacts/transcriptional activation (numerous examples of these mechanisms have been proposed: Mediator complex, Ldb1, YY1,etc). Or maybe enhancers and promoters attract each other given that they share chromatin modifications and may be bound by similar types of chromatin regulators. If this is true, cohesin retention at CTCF would form loops that could reduce the ability of these processes to promote contacts and transcription by placing enhancers and promoters more frequently in different loops.

We completely agree with the idea that enhancer/promoter contacts are multifactorial. We have stated in our manuscript that loop extrusion is one of potentially several other mechanisms at play in enhancer-driven expression of alpha-globin. As the reviewer stated, there is now good evidence for other structural proteins mediating enhancer-promoter interactions and subsequent gene expression and these are discussed in the text. We have shown that loop extrusion is potentially involved in the establishment of enhancer-promoter interactions and gene expression (early in differentiation) and not later. As part of a separate project, we are indeed investigating other mechanisms. In the field, we acknowledge that the question of enhancer-promoter interactions is complex as we have stated in the answer to the first question. It is difficult to explain why cis-elements in independent loops could not interact since they would still be free to do so within a three-dimensional structure. However, if their interaction depends, to some extent, on a tracking mechanism, this would explain why cis-elements such as CTCF insulators, active promoters and elements bound by MCM proteins etc can block enhancer-promoter interactions.

This could happen independently of a potential role of Cohesin in inducing enhancer-promoter contacts. CTCF/cohesin would be able to reduce contacts and reduce activation but this molecular bridge could still force contacts across CTCF-anchored loops. Even if cohesin is indeed important to promote enhancer contacts–which very likely is true–the experiments this paper focuses on, are not a direct way to assess this.

CTCF Insulators are known to prevent translocation of cohesin. The molecular mechanism is well understood and it is unclear what a more direct experiment would look like. It would be helpful to get some feedback on exactly what experiments the reviewer has in mind so that these could be discussed in the text.

Therefore, in its essence this study focuses on the ability of CTCF loops to block enhancer-promoter contacts, a topic that has been well explored recently.

There are interesting observations in figure 1 that aren't really explored. For example, are other genes affected by cohesin loss? If that's the case which genes are affected and which ones are not. Do we always see a reduction in contacts? Are there genes that rely more on cohesin at later stages versus early stages? What characterizes them?

The varied effect of cohesin depletion on the transcriptome has been documented , such as Rao et al. (Cell 2017)[6], Wutz et al. (The EMBO journal 2017)[7], Cuartero et al. (Nature Immunology 2018)[8] and more recently with the NIPBL depletion that showed the difference between two loci (Sox2 and Car2) that responded differently to the loop-extrusion disruption (Hansen et al. Science 2025) [9]. In our study, apart from the alpha-globin genes, we have also investigated the effect of cohesin depletion on the beta-globin which we have now included in the manuscript (Fig 1c, Supp Fig. 2 and 3).

A genome-wide evaluation of the effect of cohesin depletion in our cell culture (early and late in differentiation) might be an interesting future extension to our study. However, we might end up with yet another list of genes that are affected versus genes unaffected. Our locus-specific approach allowed us to tackle the question in a more targeted and controlled manner by which mechanisms could be better examined. Examining other affected or unaffected genes will require these loci to be studied in similar detail to the alpha globin locus and to Nora's paper (Hansen et al. Science 2025) [9]. As always in our work, we aim to establish principles underpinning gene regulation at a model locus. There may be many exceptions but based on past history, principles established at the globin loci have been generally true for a large proportion of mammalian genes.

What happens to the structure of this locus upon cohesin degradation? Do we see reduced enhancer promoter contacts?

This is an important question which we did not prioritise since, given the size of effect of RNA expression observed, we did not anticipate a significant easily detectable effect on enhancer-promoter contact. Nevertheless, we have now generated data to address the reviewer's question. We performed a Capture-C experiment to interrogate the interaction between promoters and enhancers at the alpha-globin locus (from three viewpoints, enhancer R2 and promoters of Hba-a1 and Hba-a2) upon Rad21 depletion in the EB-derived erythroid cells both early (day 5) and late (day 7) in erythroid differentiation. We observed a consistent reduction in interactions between promoters and enhancers upon cohesin depletion that seems more prominent in early differentiation when alpha-globin gene expression is most affected. However, as predicted, the reduction in contact based on DESeq, is not statistically significant. This is not surprising given that the enhancer-promoter interaction may be transient and/or infrequent and therefore may not easily correlate with transcription and RNA expression (e.g. Zuin et al Nature 2022 [10]); furthermore cohesin was only removed for 6 hours, and the level of RNA expression was only decreased by 30% . This full set of data is now included in Supp. Fig. 6.

Supplementary Fig. 6

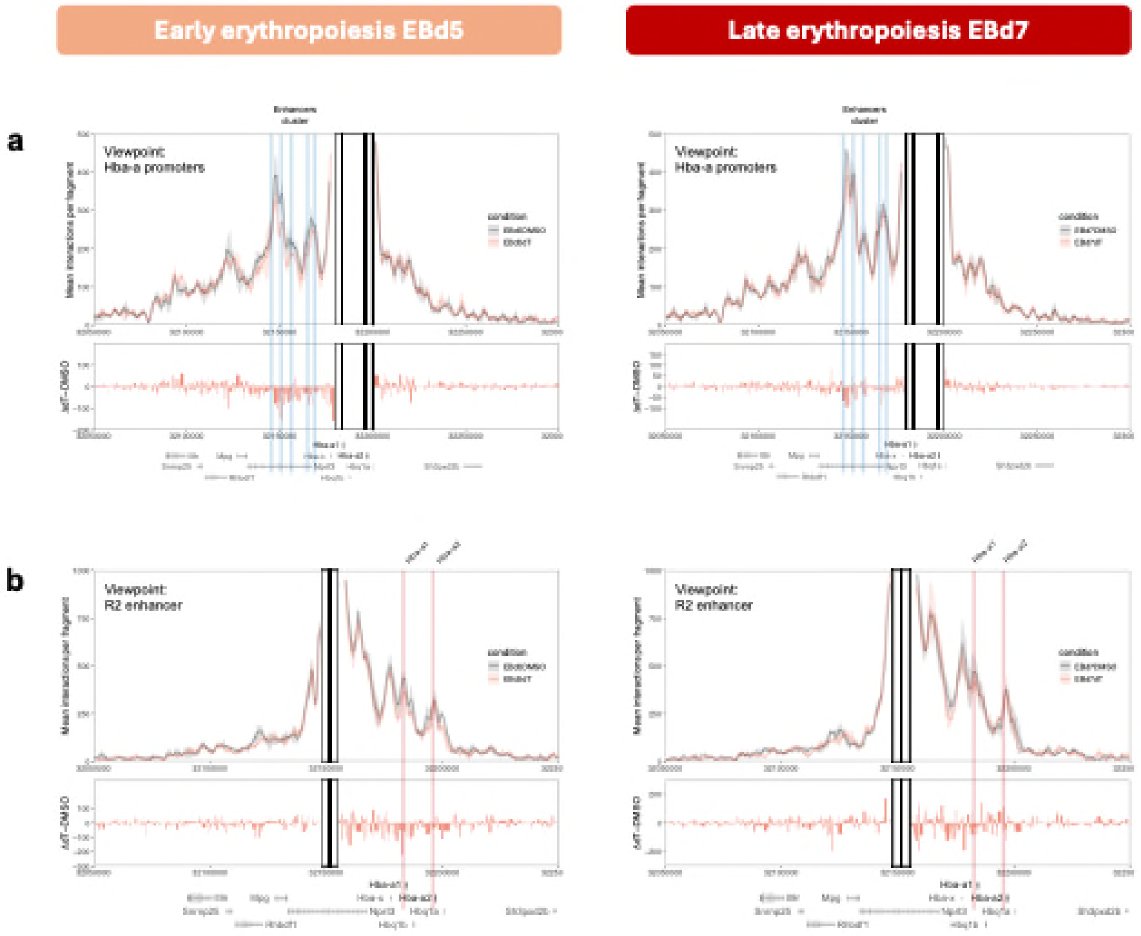

It would also be interesting to better characterize Cohesin recruitment and show that indeed there is more loading from the enhancer side than from the gene side. Showing H3K27ac tracks that the authors surely already have together with maybe NIPBL could be helpful.

Our Rad-21 ChIP-seq data shows that the accumulation of cohesin (Rad21) on both the enhancers (highlighted in grey) and the promoters (highlighted in pink) are at comparable level (Fig 4). CTCF sites are shown in blue. Our previously published ChIP-seq data shows that NIPBL is highly enriched at the alpha-globin enhancers (especially on R1 and R2) when compared to the promoters (Hua et al. 2021 Nature, extended data figure 9). The report also includes H3K27ac ChIP-seq, showing equal distribution across the locus (Hua et al. 2021 Nature)[11].

Cohesin recruitment evidence:

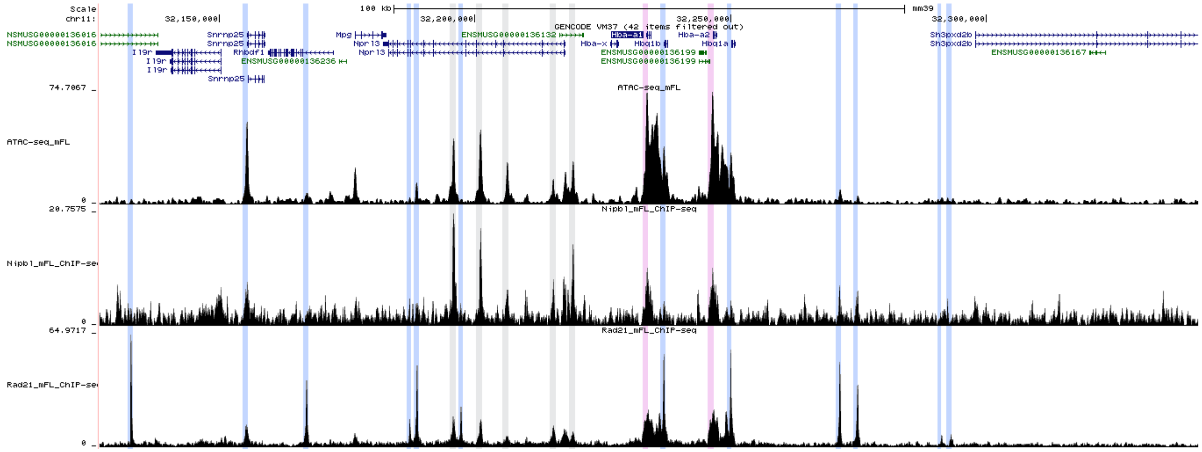

Hua et al. 2021 Nature, extended data figure 9a:

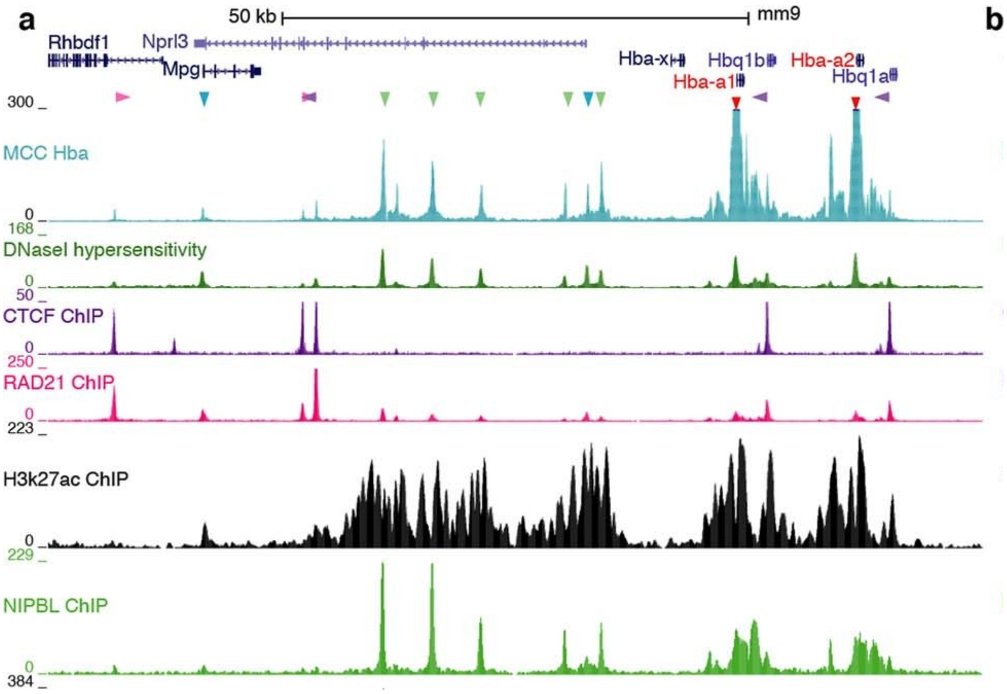

Considering that this paper in its essence is characterizing the ability of CTCF motifs to block enhancer-promoter communication it would be important to cite the numerous papers that have also studied this phenomenon directly. I believe none of these papers are cited (33229569, 31759823, 35710138, 36717694, 38084924).

These papers focus more on the role of CTCF at TAD boundaries which immediately evokes a different scale (100s-1000s kb) and different architectural considerations involving many uncharacterised elements but including CTCF sites. Moreover, some of these papers examine the dispensability of CTCF sites in creating and maintaining a transcriptional program. This is not the scientific question nor the function for which we have deployed the CTCF sites here. These references give examples of how E-P interactions can be established with varying levels of CTCF influence, which supports the hypothesis of loop extrusion (or other mechanisms) driving E-P contacts rather than a CTCF-anchored loop-structure. In our work, we use CTCF as a tool to block loop extrusion and demonstrate its effect on gene expression. We have now included a review of these previous papers in the discussion (line 648).

Other small points:

How are the cells' cell cycle affected by cohesin depletion?

This is an interesting question for another project and another experimental design. We have restricted the duration of cohesin depletion to the minimum (6 hours) that allows total depletion of the protein and enough time for alpha globin expression read-out. Only a small proportion of cells would undergo cell division in the 6-hour experimental period and consequently we have not addressed this point.

The resolution of plots in Figure 1 is very bad and text almost impossible to read.

This issue has been addressed.

It would be nice to see the HiC structure of this locus in the WT configuration before CTCF insertion.

Our response: We have previously published the Tiled-C contact matrices to illustrate the genomic architecture of the alpha-globin locus in wild type erythroid cells (Oudelaar et al. 2020 Nature Comm, figure 4) [12].

Reviewer #2 (Remarks to the Author):

To examine the effects of cohesin-driven loop extrusion on enhancer-promoter connectivity, the authors deplete the cohesin subunit RAD21 or insert a CTCF site between the alpha-globin enhancer and promoter. Alpha-globin transcription is diminished upon cohesin depletion and also upon CTCF site insertion. The later effect is stronger when the CTCF site blocks extrusion initiating from the enhancer and less so when facing the promoter. The authors conclude that in this system chromatid extrusion can facilitate enhancer-promoter contacts. A notable finding here is that cohesin may be required in a case of short(er) range (30-50kb) interactions. The experiments were carried out in biologically relevant systems and results are clear.

I have the following comments/questions.

1. Effects of cohesin depletion are not as strong as one might expect if the powerful enhancer were no longer able to loop to the promoters. This seems especially true at later stages of expression (day 7) when cohesin loss has no effect in spite of high HBA transcription. There may be other explanations for the moderate effect of cohesin depletion.

The observation that cohesin depletion only modestly reduces alpha globin gene expression and that Capture-C analysis does not show a complete loss of enhancer-promoter interaction, is not unexpected. Technically, in our experimental setting, cohesin was only depleted for 6 hours, which may not be sufficient to reveal the full extent of cohesin loss. Longer depletion would compromise cell division and the cell cycle. Moreover, we do not propose loop extrusion as the sole mechanism responsible for bringing enhancers and promoters together. Other mechanisms may act alongside. Our findings that cohesin depletion has a stronger effect on alpha globin expression during early erythroid differentiation suggest that cohesin may play an important role in establishing the initial enhancer–promoter contacts. At later stages, these contacts may be maintained by additional mechanisms, making the role of cohesin less prominent.

2. Related to point #1, the authors state that “It is possible that the lack of effects of cohesin depletion on gene expression seen in previous experiments reflects the fact that loop extrusion is particularly important for establishing enhancer–promoter communication during differentiation or in response to stimuli”. I am not sure I understand this logic. In the time between day 5 and day 7, the cells must have undergone several divisions and therefore had to establish enhancer-promoter contacts de novo in the progeny. Moreover, according to the text, between day 5 and day 7 alpha-globin expression continues to rise.

In our experimental set-up, the Rad21 depletion is happening in two separate cell populations for a short period of time (6 hours); in erythroid progenitors (CD71+ cells) purified from EB day 5 cultures and, separately, more differentiated erythroid cells (CD71+ cells) purified from EB day 7 cultures. The cells have not been continuously cohesin depleted as they transition from day 5 to day 7; this would be impossible to do in the absence of cohesin which is required for cell division, concomitant with differentiation. What we have reported here is a snapshot at an early and late stages of differentiation upon acute depletion of cohesin. While poorly understood at present, the observation is clear and reproducible. Our interpretation is that cohesin may play an important role in establishing initial enhancer–promoter contacts during early differentiation. At later stages, these contacts may be maintained by additional mechanisms such as architectural proteins and histone modifications established at enhancers and promoters earlier in differentiation and preserved through mitosis (Lam et al. 2023 Nat Genet. [13]; Wenger et al. 2023 Nat Genet. [14]) which can support subsequent enhancer–promoter interactions independently of loop extrusion.

3. The authors try to attribute the lack of effects of cohesin depletion in prior studies to incomplete degradation (line 422). What are the cohesin ChIP-profiles -/+ dTag ligand at day 5 vs day 7?

Our response: By Western blot, we show that the degradation of cohesin by dTAG is complete, both in day 5 and day 7 EBs. Here we have shown the Rad21 ChIP meta-analysis performed on day 7 showing genome-wide reduction in Rad21 recruitment upon cohesin depletion.

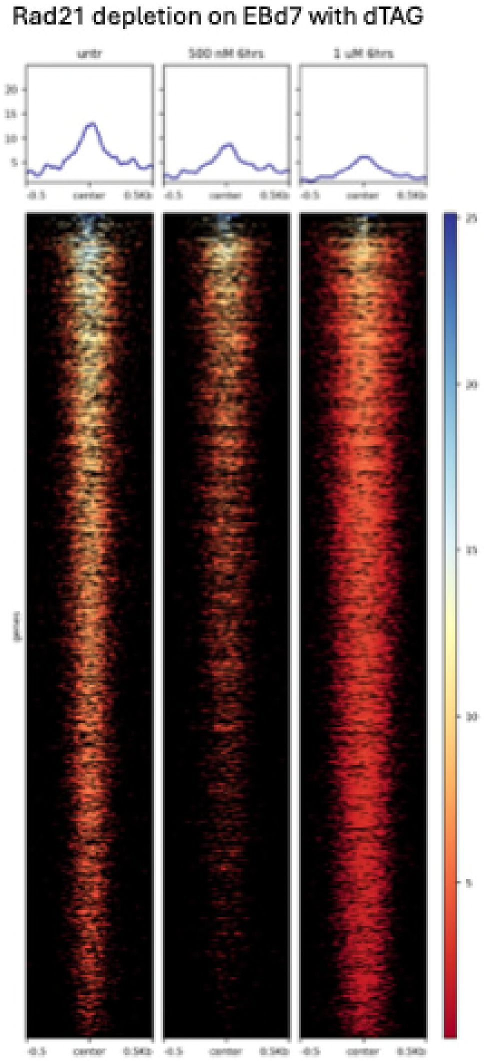

4. There is mounting evidence that cohesin can affect transcription factor (TF) occupancy. Have the authors considered measuring a few key erythroid TFs upon cohesin depletion? This might be an alternative explanation if cohesin is degraded or cannot reach TF binding sites.

This is another interesting angle that has not been explored here because it was not part of the original question that specifically set out to examine the effect of compromising loop extrusion on transcription. We could certainly address this aspect in future studies and will add this to the text. However, reporting simply a low TF binding in ChIP data will not tell us whether we are reading a cause or a consequence of a compromised enhancer-promoter interaction and weaker expression.

5. Fig.1A: please show absolute HBA expression levels not set to “one” to evaluate changes in HBA expression and include a control/point-of-reference that allows the reader to judge expression levels.

In response to the reviewer’s comment, we have now included a new analysis of qPCR results (2^delta ct) that allows assessment of raw expression of the genes (Supp Fig. 2). We have also generated new, more quantitative digital droplet PCR (ddPCR) data to confirm expression changes observed in the qPCR experiment (Supp Fig. 3). It should be noted that the manuscript also included smFISH data to help evaluate the nascent transcription levels (Fig 1d, 1e).

EBd5 qPCR data presented in 2^delta ct:

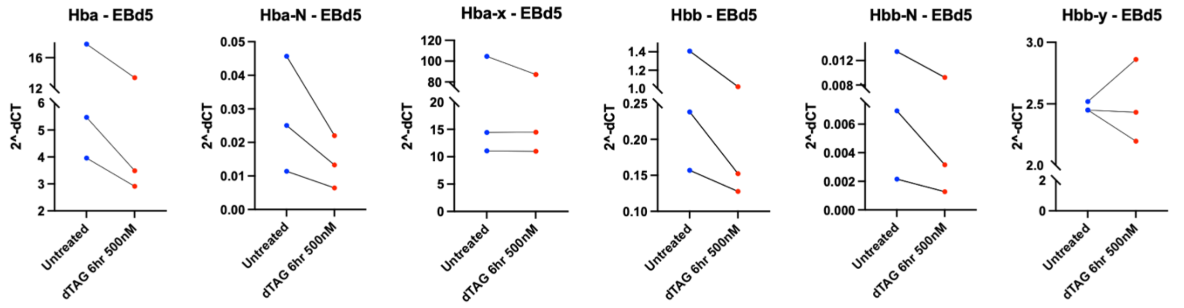

EBd6 qPCR data presented in 2^delta ct:

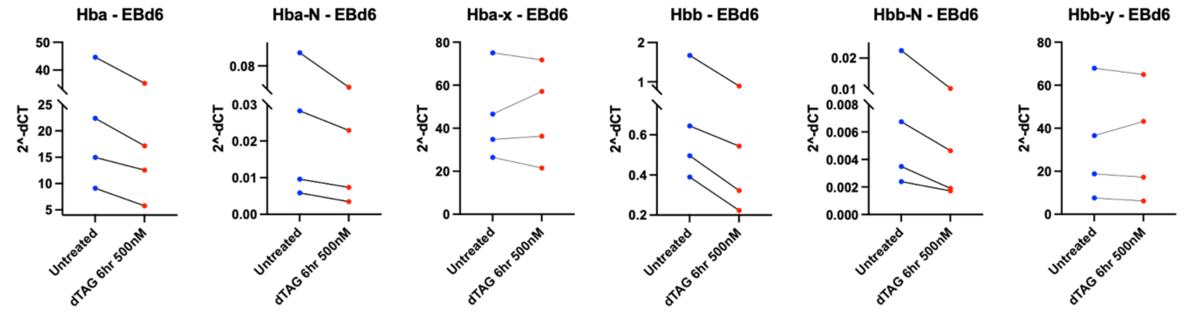

EBd7 qPCR data presented in 2^delta ct:

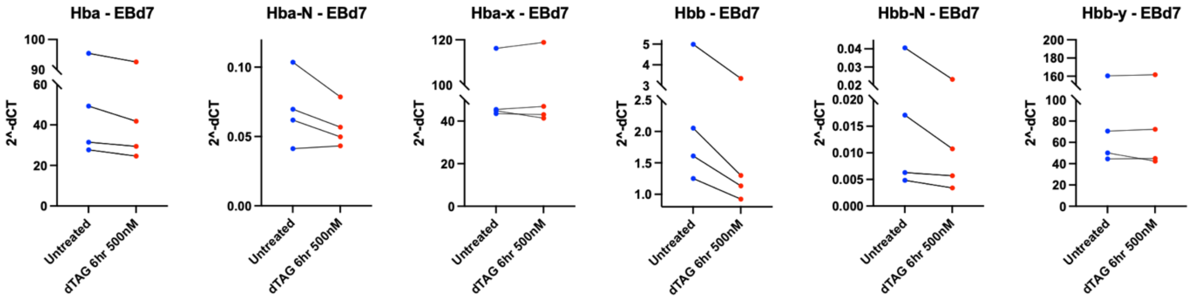

EBd5 ddPCR data:

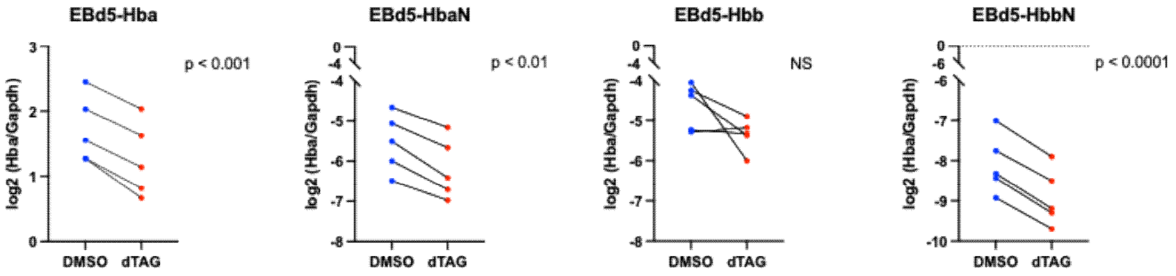

EBd6 ddPCR data:

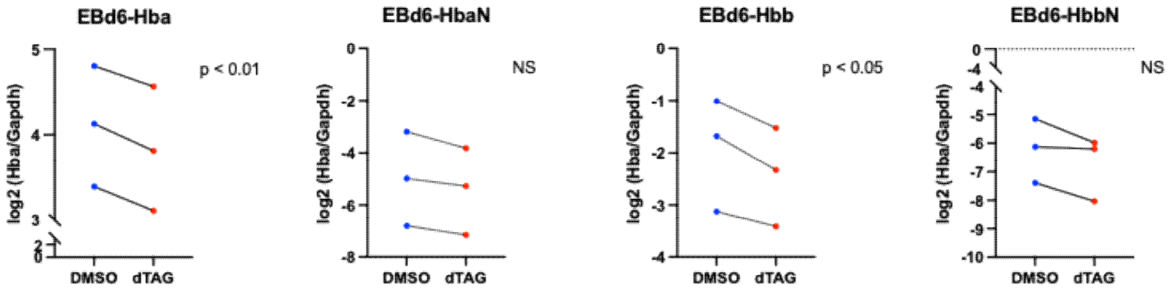

EBd7 ddPCR data:

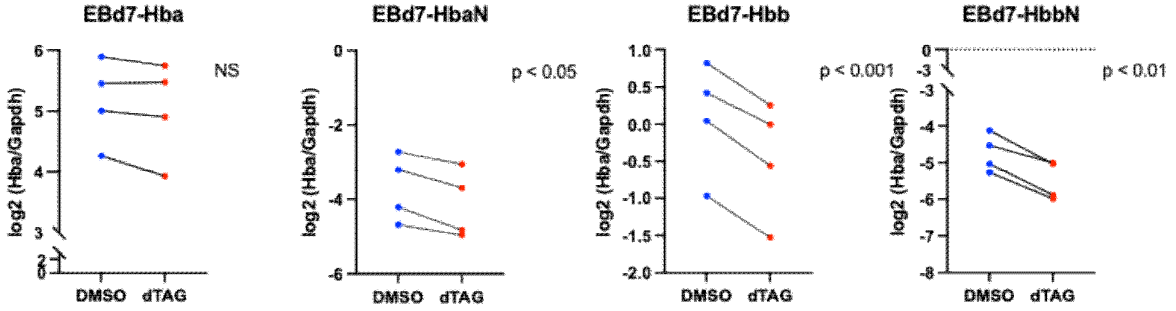

6. As to their EB system, the cells don't seem to traverse distinct stages of differentiation culminating in CD71+ Ter119- cells, but rather display this somewhat odd diagonal.

The cells transition from predominantly CD71+/Ter119- to predominantly CD71+/Ter119+. We have revisited the compensation and gating on these FACS profiles and now included more accurate representative plots clearly showing these populations (Fig. 1a). We have also performed cytospins stained with MGG to further show different stages of differentiation (Fig. 1b). We have previously analysed this differentiation system in detail (Francis et al. 2022 PLoS One) [15].

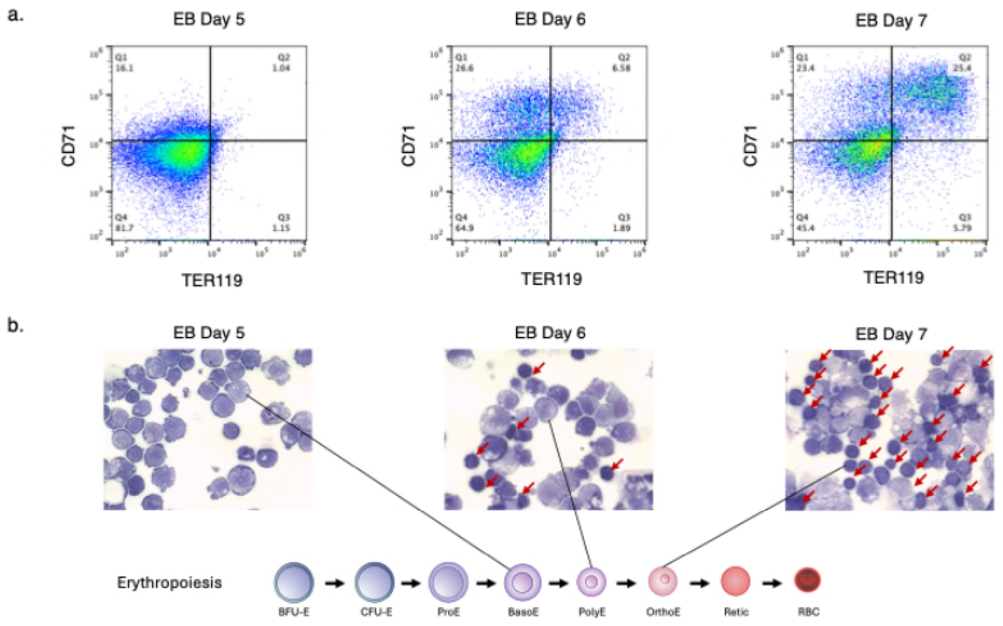

Sorting CD71+ cell via beads will result in a mixture of maturation stages. Please comments. Also, is there any leakiness of their degradation system? Is it possible that the Rad21 tagged cells are not undergoing normal erythropoiesis?

Our response: By all tested criteria, the Rad21 tagged cells appear to undergo normal erythropoiesis. The CD71+ cells isolated at day 5 of EB differentiation are of more immature in nature compared to those purified at day 7, as shown in the FACS plots and cytospins above. The dTAG-mediated degradation is done for only 6 hours and on cells that have already been purified at the days indicated. We analyse the cells after 6 hours, with and without dTAG and do not observe changes in survival or differentiation under these conditions.

7. Why are there fewer instead of more FISH foci at day 7 compared to day 5 if HBA expression is higher at day 7? Does burst size increase at day 7 vs day 5? The size of the foci as a function of burst intensity needs to be quantified.

This is a well-described phenomenon that as the proportion of alpha globin RNA increases in late differentiation, the primary transcripts are decreasing (Jeziorska, Tunnacliffe et al. 2022 Nat. Comm) [16]. The relative increase in globin mRNA is due to its unusually long half-life.

8. Only one replicate of Rad21 ChIP EB Rev is reported (Fig. 4). Can the authors comment on whether/why additional replicates were excluded? Since these data underpin one of the key conclusions of the paper and are the basis for interpreting subsequent experiments, it is important to show at least two replicates. Another point re Fig. 4a is that it would be easier to interpret the results if the same scales were used for each antibody/system.

As suggested by the reviewer, we have repeated the Rad21 ChIP-seq on three biological replicates of the EB (forward and reverse) and mouse spleen (forward and reverse) models. We have tested different antibodies for the ChIP-seq experiment and included here data from a new Rad21 antibody (ab154769, Abcam), which was more reproducible and robust. With this new antibody, we recapitulate previous observations in the mouse spleen data, where we report that the CTCF-reverse insertion site accumulates significantly more cohesin than the CTCF-forward insertion site. However, this was not observed in the EB-derived dataset.

We reasoned that this may be due to the different nature of the erythroid cells in these two systems. Erythroid cells in the mouse spleen are adult (referred to as definitive) erythroid cells, while those in the EB system are mainly embryonic (or primitive) (Francis et al. 2022 PLoS One) [15]. The key difference between definitive and primitive erythroid cells is that Hba-x is actively transcribing in the primitive erythroid cells. It is possible that cohesin may also be recruited at the active Hba-x promoter, contributing to the accumulation of cohesin at the CTCF-forward insertion site.

a.

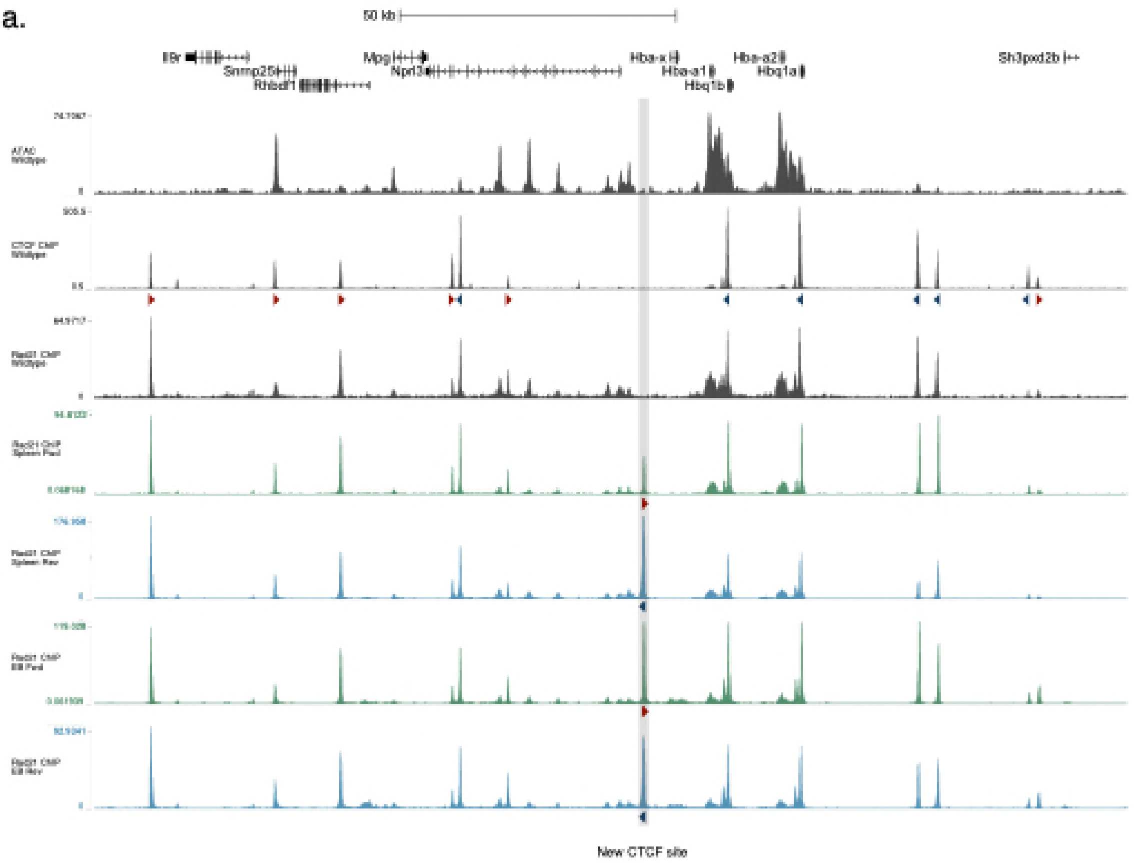
50 kb
Il9r
Snmp25
Rhbdf1
Mpg
Nprl3
Hba-x
Hba-a1
Hbq1b
Hba-a2
Hbq1a
Sh3pxd2b
New CTCF site

b.

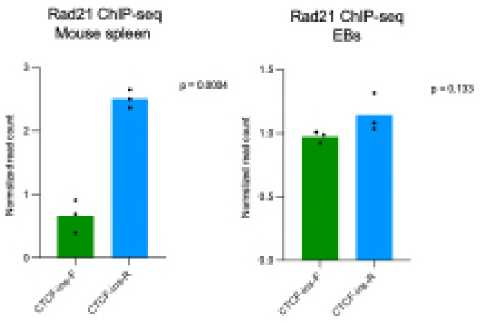
Rad21 ChIP-seq
Mouse spleen
p = 0.0084
Rad21 ChIP-seq
EBs
p = 0.133
Normalized read count
CTCF-ins-F
CTCF-ins-R

c.

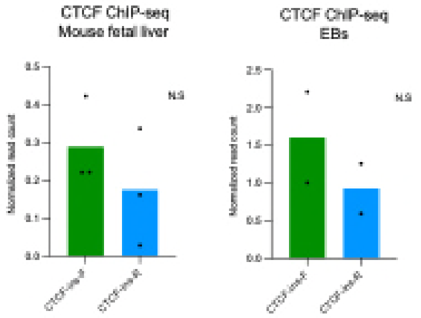
CTCF ChIP-seq
Mouse fetal liver
N.S
CTCF ChIP-seq
EBs
N.S
Normalized read count
CTCF-ins-F
CTCF-ins-R

9. It may be useful to quantify changes of other cohesin peaks within this locus. That may provide additional insights as to whether/which loops are altered after the CTCF insertion (reflected by decreased/increased cohesin at convergent pairs) versus whether the new CTCF site primarily affects loop extrusion (uniform change in cohesin across peaks).

In response to the reviewer's point, we have now quantified the Rad21 ChIP-seq on all CTCF binding sites across the alpha-globin locus in the Forward and Reverse insertion EB models. Rad21 enrichment was significantly increased at the CTCF binding sites upstream of the inserted CTCF binding site in the Reverse model over the Forward model. Alternately, increased Rad21 enrichment on HS+44, the CTCF binding site downstream of the inserted CTCF binding site, was observed in the Forward model over the Reverse model. These observations support the Capture-C data, showing that ectopic insertion of the CTCF binding site altered cohesin-mediated loop extrusion and 3D genome organisation. This data is shown in Supp Fig. 12.

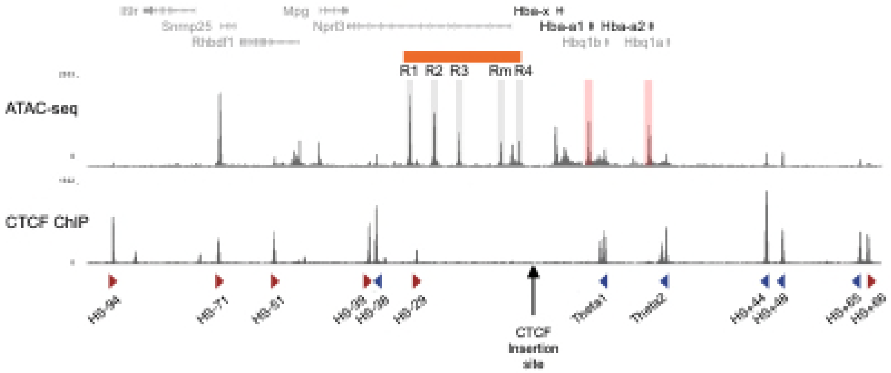

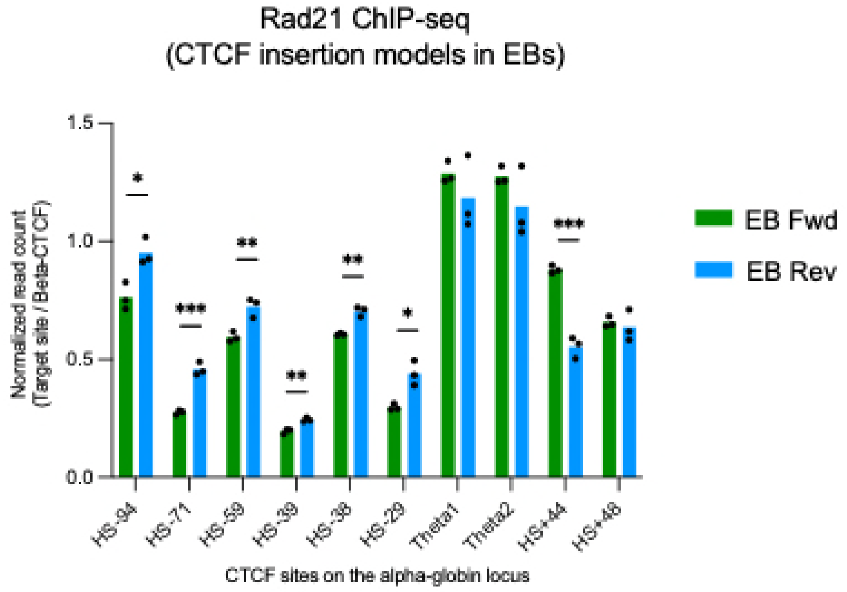

10. Fig.S3: Why are there no cells with 3 or 4 foci? Is HBA only expressed in G1-phase?

There are a few cells with 3 or 4 foci. About 2-13 cells with 3 foci, and about 0-2 cells with 4 foci in the whole analysis. These are highlighted in a bar chart in supplementary figure 5.

11. Fig.5: Would it be helpful to sort the erythroid cells and look at the differentiation stage at which CTCF/cohesin function might have the most pronounced effects? This question relates to the claim in Fig.1 that cohesin has a stronger effect early on.

We are satisfied with the current system that provides enough separation between the erythroid stages of differentiation just by purifying day 5 versus day 7 CD71+ cells without compromising the cell numbers required to perform informative assays including ChIP-seq and 3C-based assays. Also, based on our experience with this in vitro EB differentiation system, an immunophenotypic separation at day 7 based on CD71+/Ter119+ is not as powerful in separating the various erythroid stages of differentiation as in primary cells (S0-S5 populations in fetal liver and bone marrow cell populations, Pop et al, 2010 [17]).

12. The ATAC-seq in Figure 6 (top panel) suggests that the forward and reverse clones have different accessibility landscapes. Can the authors quantify accessibility at relevant peaks and comment on how differences in accessibility may affect the proposed loop extrusion model?

The main difference we observe is in the peaks over the alpha-globin genes in the reverse model, which are reduced. This is associated with the significantly reduced expression levels.

13. Line 271: the upregulation of Rhbdf1 and Mpg is attributed to a "disruption of TAD organization". Is it possible that this is instead due to enhancer competition because the alpha genes are now somewhat insulated from the enhancer?

We agree with the reviewer that this is a possibility and we have discussed this in the manuscript (line 409).

14. It would be interesting to test whether enhancer blockade by CTCF requires a loop by removing the other CTCF loop anchor. This would address whether cohesin-dependence is due to the extrusion process per se, or cohesin-dependent CTCF loops. I think this is important because the title of the manuscript suggests the former but from my reading of the paper does not rule out the latter.

To test the reviewer's hypothesis requires extensive and repeated rounds of genomic engineering to ensure removal of several (~6) potentially interacting CTCF binding sites spread across the locus. In a separate project, we are testing such models. Moreover, a CTCF site can function as a boundary element independently; this has been shown elegantly in a paper from Vos et al. (2021 Mol. Cell) [18]. Moreover, in a different study (Cornell and Harold et al, in press) [19], we have observed a similar effect at the alpha-globin locus; when the CTCF sites at -38/-39 (upstream of the super-enhancer) are intact we see no effect on alpha globin expression when the downstream CTCF sites in the cluster are removed. It is speculated that this is true when a copy of these elements is introduced at the newly designed landing pad.

15. The authors should discuss related papers from Ann Dean's lab (PMCID: PMC519119), 2006 (PMID: 16877759) and especially the 2008 Hou et al. PNAS paper (PMCID: PMC2629272) in which a CTCF site was inserted between the beta-globin enhancer and gene in engineered mice. It is also worth mentioning the 2003 Tanimoto et al paper (PMCID: PMC309639).

We agree with the reviewer that some of these studies should have been mentioned and we have now included them in the discussion of the revised manuscript (line 634). It is worth highlighting the caveats in these papers where one involved insertion of a 2.6kb fragment containing a single CTCF site and the other experiments were carried out in a *human* BAC randomly inserted into a *mouse* background. Although a *tour de force* 20 years ago, we do not think that this would necessarily be accepted by the rigour of today's reviewers.

Minor:

1. Lines 41 and 321: I suggest not equating enhancer-promoter contacts and sub-TADs. They are distinct entities.

We have revised the wording in line 41 to avoid any potential confusion. Regarding line 321, our statement does not equate sub-TADs and enhancer-promoter contacts; rather, it indicates that the enhancer and promoter come in close juxtaposition within the same sub-TAD, which provides the structural context for their interaction.

2. Line 280: The reporter assay should be described a bit more in the text or shown in a cartoon, and made clear that the insertions are still done at the endogenous locus if that's the case.

It is the case and a major advantage of this work. The reporter assay is described in detail in our recent publication (Tsang et al. NAR 2024) [20]. We have added a diagram of the reporter assay in the Supp Fig. 11.

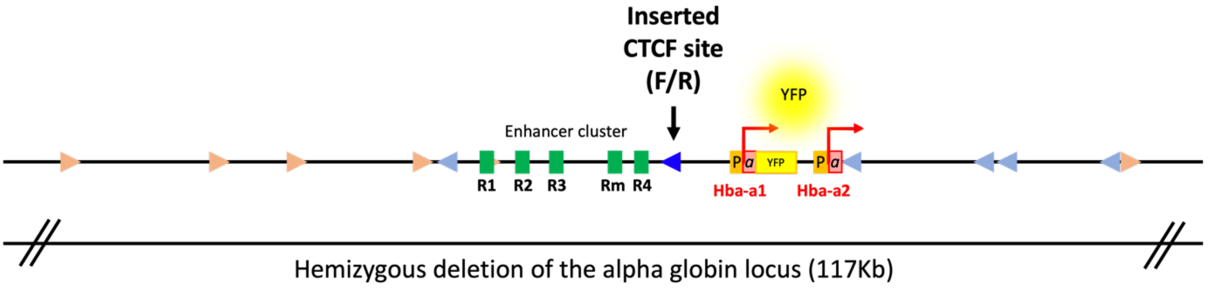

3. In the Results/Discussion, it may be more precise to state that cohesin translocates from the "enhancer side" rather than from the "enhancers" themselves since there was no demonstration where the accumulated cohesin was originally loaded.

We agree with the reviewer and we have revised this in the manuscript.

References:

1. Yang, J.H. & Hansen, A.S. Enhancer selectivity in space and time: from enhancer-promoter interactions to promoter activation. *Nat Rev Mol Cell Biol* **25**, 574-591 (2024).
2. Robles-Rebollo, I. et al. Cohesin couples transcriptional bursting probabilities of inducible enhancers and promoters. *Nat Commun* **13**, 4342 (2022).
3. Rinzema, N.J. et al. Building regulatory landscapes reveals that an enhancer can recruit cohesin to create contact domains, engage CTCF sites and activate distant genes. *Nat Struct Mol Biol* **29**, 563-574 (2022).
4. Li, Y. et al. The structural basis for cohesin–CTCF-anchored loops. *Nature* **578**, 472-476 (2020).

5. Pugacheva, E.M. et al. CTCF mediates chromatin looping via N-terminal domain-dependent cohesin retention. *Proceedings of the National Academy of Sciences* **117**, 2020-2031 (2020).
6. Rao, S.S.P. et al. Cohesin Loss Eliminates All Loop Domains. *Cell* **171**, 305-320.e24 (2017).
7. Wutz, G. et al. Topologically associating domains and chromatin loops depend on cohesin and are regulated by CTCF, WAPL, and PDS5 proteins. *The EMBO journal* **36**, 3573-3599 (2017).
8. Cuartero, S. et al. Control of inducible gene expression links cohesin to hematopoietic progenitor self-renewal and differentiation. *Nature Immunology* **19**, 932-941 (2018).
9. Hansen, K.L. et al. Synergy between regulatory elements can render cohesin dispensable for distal enhancer function. *Science*, eadt4221 (2025).
10. Zuin, J. et al. Nonlinear control of transcription through enhancer-promoter interactions. *Nature* **604**, 571-577 (2022).
11. Hua, P. et al. Defining genome architecture at base-pair resolution. *Nature* **595**, 125-129 (2021).
12. Oudelaar, A.M. et al. Dynamics of the 4D genome during in vivo lineage specification and differentiation. *Nat Commun* **11**, 2722 (2020).
13. Lam, J.C. et al. YY1-controlled regulatory connectivity and transcription are influenced by the cell cycle. *Nat Genet* **56**, 1938-1952 (2024).
14. Wenger, A. et al. Symmetric inheritance of parental histones governs epigenome maintenance and embryonic stem cell identity. *Nat Genet* **55**, 1567-1578 (2023).
15. Francis, H.S. et al. Scalable in vitro production of defined mouse erythroblasts. *PLoS One* **17**, e0261950 (2022).
16. Jeziorska, D.M. et al. On-microscope staging of live cells reveals changes in the dynamics of transcriptional bursting during differentiation. *Nat Commun* **13**, 6641 (2022).
17. Pop, R. et al. A key commitment step in erythropoiesis is synchronized with the cell cycle clock through mutual inhibition between PU.1 and S-phase progression. *PLoS Biol* **8**(2010).
18. Vos, E.S.M. et al. Interplay between CTCF boundaries and a super enhancer controls cohesin extrusion trajectories and gene expression. *Molecular Cell* (2021).
19. Harrold, C.L. et al. A functional overlap between actively transcribed genes and chromatin boundary elements. *bioRxiv*, 2020.07.01.182089 (2020).
20. Tsang, F.H. et al. The characteristics of CTCF binding sequences contribute to enhancer blocking activity. *Nucleic Acids Res* **52**, 10180-10193 (2024).

RESPONSE TO REVIEWERS' COMMENTS

Reviewer #2 (Remarks to the Author):

The authors have worked hard to address all the comments, and the manuscript is improved. I have a few suggestions that require no experimentation but should be considered in the text.

1. I suggest that the title and main conclusions should be modified to represent what is actually happening. Cohesin extrusion plays a small (not "key") role in undifferentiated cells, and no role at later stages when alpha transcription is high. This is consistent with capture-c showing no statistically significant architectural effects upon Rad21 degradation. Alpha expression is not "disrupted" but subtly decreased and only early on. Taking a step back, a title more representative of the observations might read: "Enhancer-activated gene expression is largely independent of cohesin during differentiation".

   We understand the reviewer's opinion. However, we think that we have provided enough evidence to support the revised title:

   Loop extrusion by cohesin plays a role in enhancer-activated gene expression early in differentiation

2. Regarding my previous point 2, I am afraid I was misunderstood. I was not suggesting to deplete cohesin for 3 days. As stated previously, "maintenance" of loops through later stages does not make sense as an explanation here. To the uninitiated reader, "maintenance" could be misinterpreted to mean that loops are stable for long periods of time. Cells have divided and have not "maintained" any loops. Loops are of short duration and are dissolved and established continuously. Plus, alpha expression increases throughout differentiation presumably accompanied by further increases in contacts. Together, this means that loops are formed de novo by factors other than cohesin.

   We thank the reviewer for the suggestion. We have now updated discussion, 5th paragraph (lines 483-512) to include the points raised here.

3. There are no significant architectural changes in Rad21 k.o. cells. The authors state that there are trends, but they are minor and quite similar in early and late cells when there is no effect on gene expression. The inevitable noise in the data and lack of information regarding replicate concordance precludes any firm

conclusions here. Again, this suggests that cohesin may mildly influence something else (i.e. TF occupancy) which should be considered. (can put this in the discussion)

We thank the reviewer for this helpful comment. We agree that the architectural changes observed upon Rad21 depletion are modest and that, given the level of variability and limited replicate concordance, they should be interpreted with caution. We have revised the text to avoid over-interpretation of these trends.
The absence of major architectural changes, particularly in the context of minimal effects on gene expression, suggests that acute cohesin depletion when performed at later stages of differentiation may not be sufficient to disrupt established chromatin interactions or epigenetic mechanisms already set-up at earlier stages at the α-globin locus. Instead, these findings are consistent with a model in which cohesin plays a more prominent role during the establishment rather than the subsequent events required for regulatory architecture or expression.
We also agree with the reviewer that cohesin may exert other subtle effects, for example by modulating transcription factor occupancy or local chromatin accessibility, which are not fully captured by our current study. We have incorporated this point into the Discussion and clarified that alternative mechanisms, beyond large-scale architectural changes, may contribute to the observed phenotype.

We have now updated the discussion 5th paragraph (lines 483-512) to include the points raised here.

During erythropoiesis, many genes are induced through enhancer-mediated activation, with the globin loci providing a well-established example. In our study, cohesin depletion results in downregulation of both α- and β-globin transcription specifically in early erythroid cells (day 5–6 EB-derived cells), while neighbouring widely expressed genes remain unaffected (Supplementary Fig. 4), indicating a selective effect on enhancer-dependent gene activation. Using Capture-C, we observe only modest architectural changes at the α-globin locus, consistent with a partial disruption of enhancer–promoter interactions, particularly at early stages of differentiation.

We agree that these changes are subtle and will revise the text to avoid overinterpretation; however, the stage-specific transcriptional effects suggest that cohesin is particularly important during the establishment of enhancer–promoter communication, rather than its maintenance.

In addition to its predominant role in loop extrusion, we cannot exclude the possibility that cohesin also influences other regulatory mechanisms, including transcription factor occupancy or chromatin accessibility (e.g. PMID: 36376610). This may explain why cohesin depletion in other systems shows limited effects on gene expression. At later stages of differentiation, enhancer–promoter interactions may be stabilised by alternative

mechanisms, such as protein–protein contacts, or transcription may be maintained through re-initiation independently of continuous enhancer input (PMID: 39721581).

4. Previous point 3: please show the Rad21 ChIP tracks at the alpha locus -/+ dTag ligand at day 5 vs day 7. This is essential for any reader trying to interpret cohesin's role.

   We thank the reviewer for pointing this out. We have added meta-analysis of Rad21 ChIP-seq showing the loss of Rad21 binding to chromatin genome-wide upon Rad21 depletion in Supplementary Figure 2.

5. Please reconcile in the Discussion why in day 7 EBs Rad21 degradation has no effect on alpha expression but insertion of a CTCF site does. Does CTCF function as an insulator independently of Rad21 here?

   We thank the reviewer for this important point. The apparent discrepancy likely reflects differences in the timing and duration of cohesin perturbation between the two experimental systems. In the dTAG approach, Rad21 degradation is induced acutely for a relatively short duration in a mixed population of differentiating red cells. By day 7, key aspects of chromatin organisation and regulatory interactions at the α-globin locus may already be established, limiting the impact of transient cohesin loss on gene expression.

   In contrast, insertion of a CTCF site introduces a constitutive architectural barrier from the onset of differentiation, blocking cohesin-mediated loop extrusion throughout lineage progression. This early and sustained disruption may therefore be more effective at altering enhancer–promoter communication and α-globin expression.

   We do not interpret these findings as evidence that CTCF functions independently of cohesin. Rather, the results are consistent with a model in which CTCF acts by positioning and constraining cohesin, and where the timing of perturbation is critical. To further address this, future experiments will employ longer-term cohesin depletion (e.g. NIPBL depletion) from earlier stages of differentiation to directly test the requirement for cohesin in establishing α-globin regulatory architecture.

   Discussion updated to include this point in 7th paragraph (lines 615-630).

6. Related to point 5 (Line 521):" ...insertion of multiple CTCF binding sites further reduced the alpha globin gene expression by imposing further resistance to the extruding cohesin" Is there a correlation between the number of inserted CTCF sites and Rad21 accumulation?

We thank the reviewer for highlighting this point. We have now assessed Rad21 binding in the multiple CTCF insertion model. ChIP-seq analysis reveals clear Rad21 enrichment at each inserted CTCF site, indicating that these sites act as independent cohesin accumulation points. To enable precise assignment of reads, the inserted sequences were designed to include distinguishing SNPs, allowing us to resolve Rad21 peaks at individual insertion sites. These data support a correlation between the number of inserted CTCF sites and local Rad21 accumulation, consistent with the model that additional CTCF sites impose increased resistance to cohesin-mediated loop extrusion.

Revised Results lines 359-366, Discussion line 625-630
Supplementary Figure 14 a and b, and Methods, Cell and genome editing (lines 730-740).

Minor:
Supplemental Fig.11 in foetal cells: are the gene expression changes meaningful? Looking at the y-axis the signal seems very low, similar to suppl Fig. 10c where low signals were non-significant. This could just be noise around the baseline.

These foetal-cell expression changes are near the detection threshold but consistent with the expression trends we have reported in other tissues (now Supplementary Figure 12). Some of these changes are marked as statistically insignificant but the tends seem consistent. These findings do not add much or detract from the main message in the paper. We have reported them as we found them interesting and in line with what we have previously observed in Hanssen et al, 2017 when we deleted the 5' CTCF boundary.

Changes in Lines 295-301 were introduced to address this point.

Reviewer #3 (Remarks to the Author):

This paper focuses on the role the cohesin/loop extrusion on the alpha-globin gene regulation during erythroid differentiation. The alpha-globin locus was used as a model locus to dissect principles of gene expression, with a strong CTCF binding site used as a tool to block loop extrusion and to evaluate its role on gene expression.

Major findings of this paper are the demonstration of a role for loop extrusion at relative short distances between enhancers and promoters, in the range of 30-50 kb, together with the fact that cohesin may play a role in establishing enhancer-promoter interactions rather than maintaining them during differentiation. These features may reconcile many previously published data reporting weak functional role of cohesin/Rad21 depletion, most of the time in steady-state conditions.

Of note, this is a second round of review (I was not included in the first round), and I have been asking to further comments on the author responses to initial reviewers. In general,

I found experiments well carried out and results convincing. Additionally, I found their responses to initial reviews rather convincing.

I agree with reviewer one that a more direct system could have been used, such as the one used by Hansen et al (Science 2025), i.e. acute depletion of NIPBL, which disrupts long-range cohesin-dependent interactions without compromising cell division.
However, I still think that the authors used here an elegant strategy to dissect the mechanism of loop extrusion dependency at the alpha-globin genes, i.e. by using a strong CTCF binding site to efficiently block loop extrusion between enhancers and promoters at the alpha-globin locus. Their new Rad21 ChIP-seq analysis convincingly indicates a stalled cohesin at the ectopic CTCF site between the enhancer and the promoter sites. Interestingly, the transcriptional effect seems to correlate with the degree of cohesin stalling.

We thank the reviewer for taking the time to suggest changes and include information that we believe enhanced the manuscript.

Additional points to consider:
- The authors should definitely refer to the recent Hansen et al paper in their main text. Not sure it is the case at the moment.

Hansen et al 2026 is a very relevant piece of work to this manuscript. We have cited it in the revised version; lines 500-502, 571-574.

- Since the enhancer-promoter distance is an important criterion in defining the loop extrusion dependency (over just 20 kb to require cohesin function, see Hansen et al.), the authors could also discuss this aspect. They should also indicate more precisely distances (for instance in legend Figure 2) between the enhancers, the ectopic CTCF site and the Hba-a promoters.

We have updated the discussion to include the points raised by the reviewer (lines 580-585).

We have now addressed this and specified the distances between elements in Figure 2 legend.

Actually, no functional effect is detected for Hba-x, which could be due to a too short distance from the enhancer (< to 20 kb) to be sensitive to cohesin depletion. It is also possible that this distance threshold could explain why the functional effect is more robust on Hbb compare to Hba, specially at the latest stage of differentiation in the EB model. I recommend the authors to include this in their discussion.

We have included it this point in the discussion (lines 581-586).

- For the ChIP-seq quantification of Fig.4, I found a bit weird their choice of tissues, i.e. mouse spleen derived erythroid cells for the Rad21 ChIP-seq and fetal liver for the CTCF ChIP-seq. It would be nice to homogenize the data in the same tissue.

Both FL and mSpleen are sources of an equivalent type of red blood cells: definitive (adult) erythroid cells.

- In Fig.6 and for the sake of clarity, the authors should indicate which additional peaks they are referring to in the text.

We have now pointed to the additional peak that appears specifically at the insertion site in panel b, using a red arrow.
